# Supplementary material for: Environment and taxonomy shape the genomic signature of prokaryotic extremophiles
Source: Sci Rep. 2023 Sep 26;13:16105. doi: 10.1038/s41598-023-42518-y (PMC10522608; doi:10.1038/s41598-023-42518-y)

Supplementary Data S1
Distribution of Genera–Temperature Dataset

Distribution of Bacterial Genera in Psychrophiles

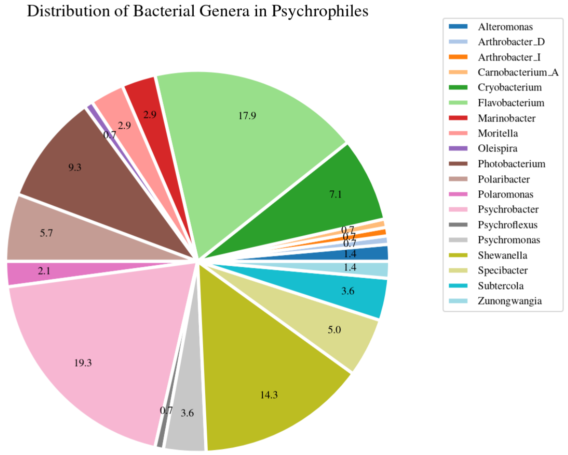

Distribution of Archaeal Genera in Psychrophiles

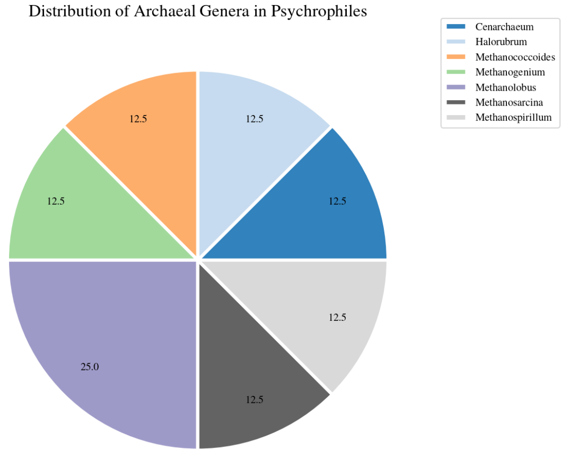

Distribution of Bacterial Genera in Mesophiles

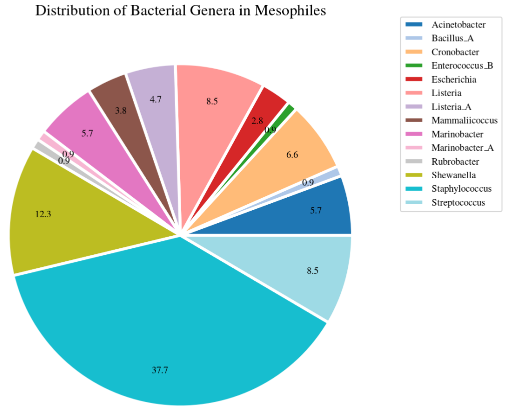

Distribution of Archaeal Genera in Mesophiles

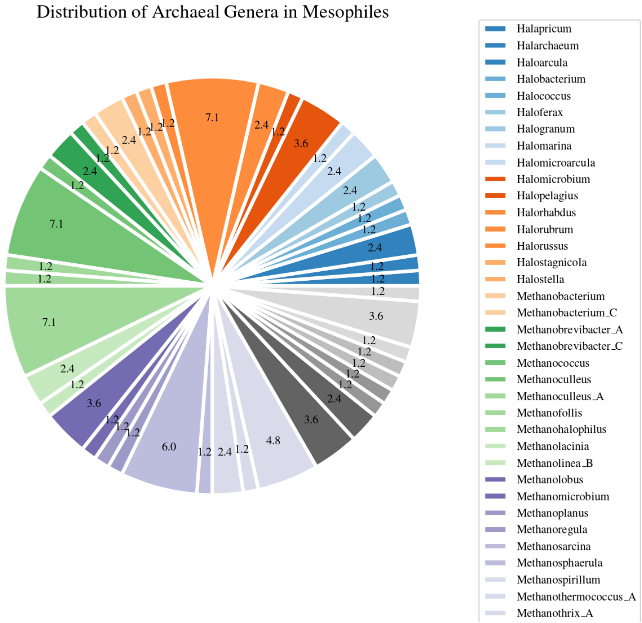

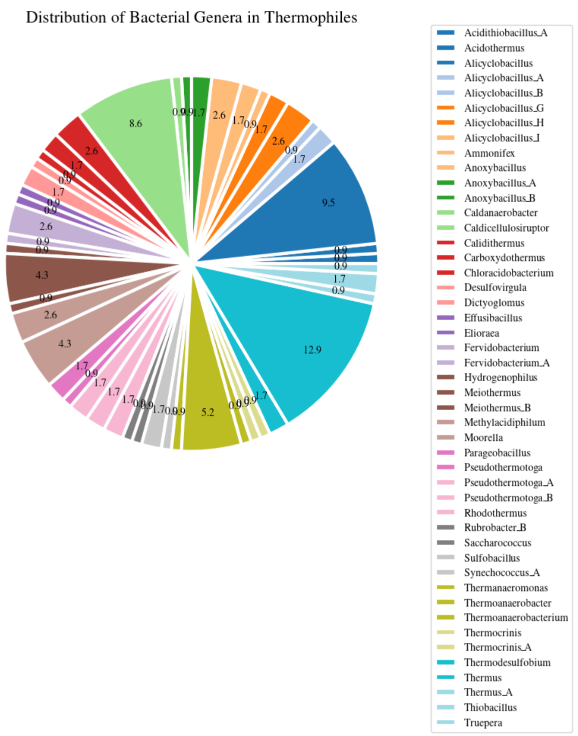
Distribution of Bacterial Genera in Thermophiles
Acidithiobacillus_A
Acidothermus
Alicyclobacillus
Alicyclobacillus_A
Alicyclobacillus_B
Alicyclobacillus_G
Alicyclobacillus_H
Alicyclobacillus_I
Ammonifex
Anoxybacillus
Anoxybacillus_A
Anoxybacillus_B
Caldanaerobacter
Caldicellulosiruptor
Calidithermus
Carboxydothermus
Chloracidobacterium
Desulfovirgula
Dictyoglomus
Effusibacillus
Elioraea
Fervidobacterium
Fervidobacterium_A
Hydrogenophilus
Meiothermus
Meiothermus_B
Methylacidiphilum
Moorella
Parageobacillus
Pseudothermotoga
Pseudothermotoga_A
Pseudothermotoga_B
Rhodothermus
Rubrobacter_B
Saccharococcus
Sulfobacillus
Synechococcus_A
Thermanaeromonas
Thermoanaerobacter
Thermoanaerobacterium
Thermocrinis
Thermocrinis_A
Thermodesulfobium
Thermus
Thermus_A
Thiobacillus
Truepera

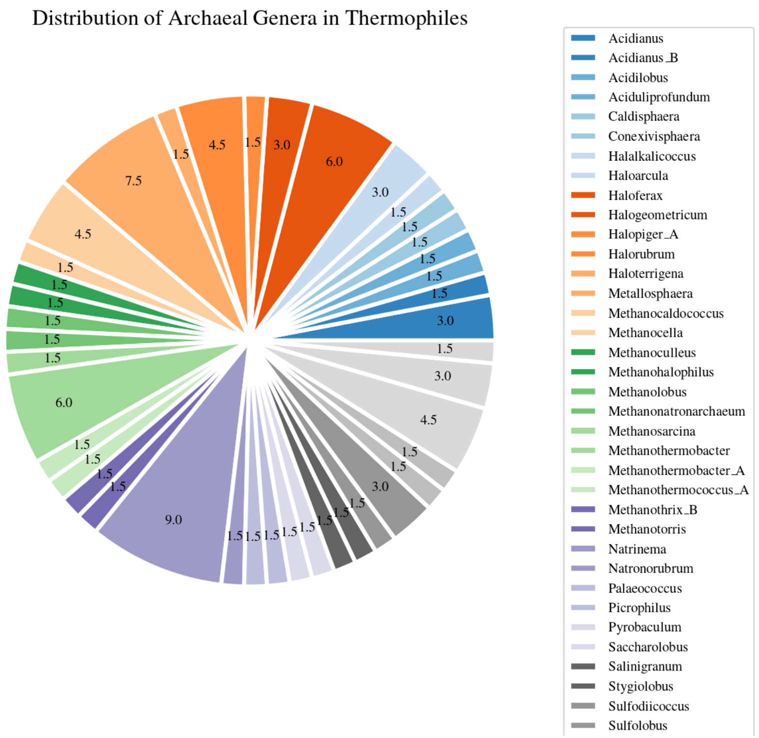
Distribution of Archaeal Genera in Thermophiles
Acidianus
Acidianus_B
Acidilobus
Aciduliprofundum
Caldisphaera
Conexivisphaera
Halalkalicoccus
Haloarcula
Haloferax
Halogeometricum
Halopiger_A
Halorubrum
Haloterrigena
Metallosphaera
Methanocaldococcus
Methanocella
Methanoculleus
Methanohalophilus
Methanolobus
Methanonatronarchaeum
Methanosarcina
Methanothermobacter
Methanothermobacter_A
Methanothermococcus_A
Methanothrix_B
Methanotorris
Natrinema
Natronorubrum
Palaeococcus
Picrophilus
Pyrobaculum
Saccharolobus
Salinigranum
Stygiolobus
Sulfodiicoccus
Sulfolobus

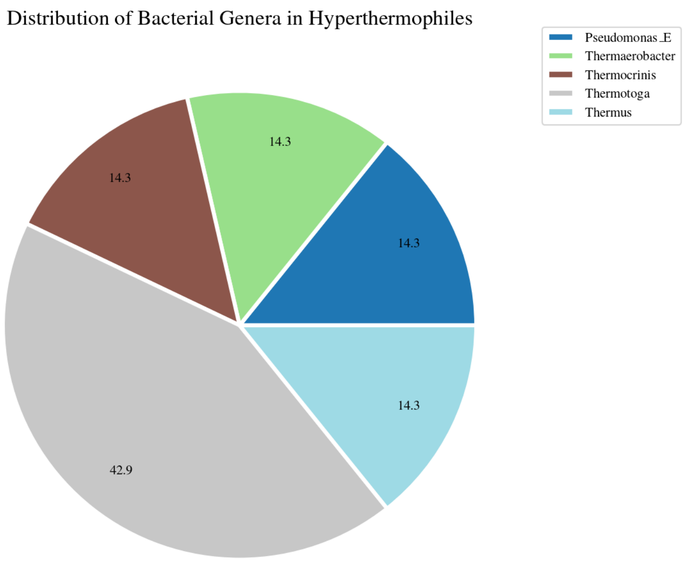
Distribution of Bacterial Genera in Hyperthermophiles
Pseudomonas_E
Thermaerobacter
Thermocrinis
Thermotoga
Thermus
14.3
14.3
14.3
14.3
42.9

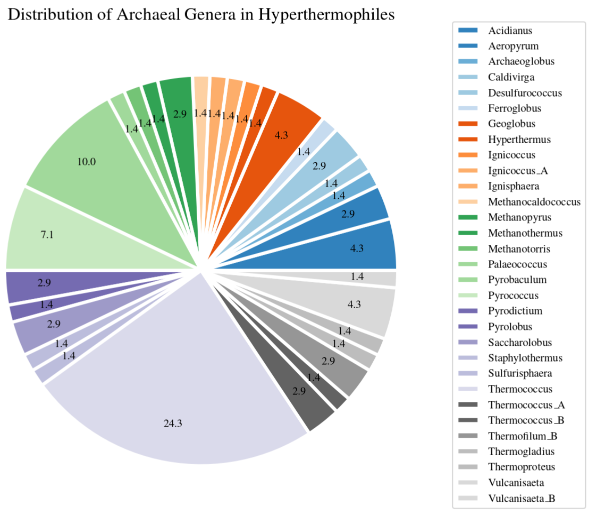
Distribution of Archaeal Genera in Hyperthermophiles
Acidianus
Aeropyrum
Archaeoglobus
Caldivirga
Desulfurococcus
Ferroglobus
Geoglobus
Hyperthermus
Ignicoccus
Ignicoccus_A
Ignisphaera
Methanocaldococcus
Methanopyrus
Methanothermus
Methanotorris
Palaeococcus
Pyrobaculum
Pyrococcus
Pyrodictium
Pyrolobus
Saccharolobus
Staphylothermus
Sulfurisphaera
Thermococcus
Thermococcus_A
Thermococcus_B
Thermofilum_B
Thermogladius
Thermoproteus
Vulcanisaeta
Vulcanisaeta_B

Distribution of Genera–pH Dataset

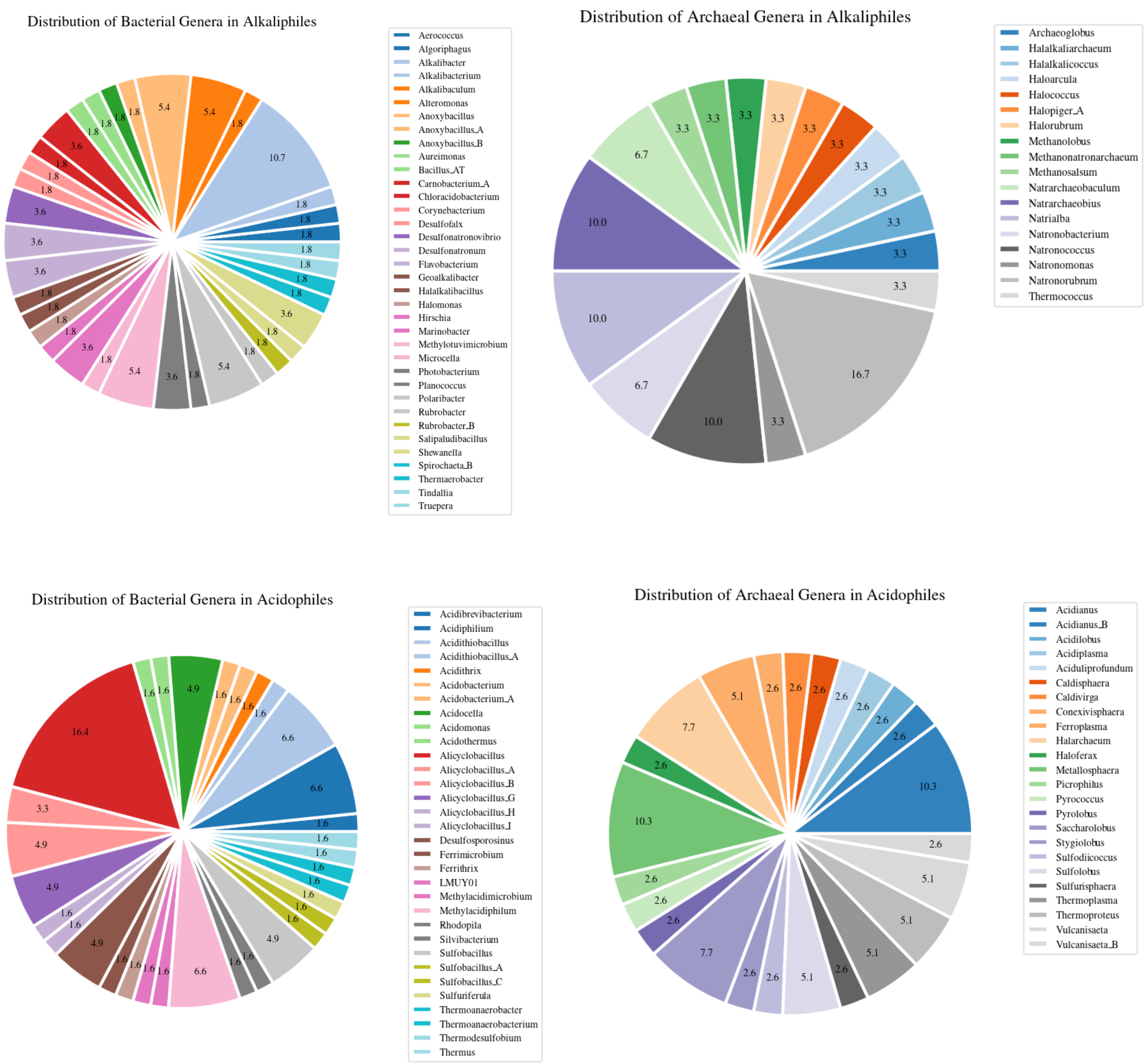

Supplement: Supplementary file 2 — Supplementary Information 2. [file 41598_2023_42518_MOESM2_ESM.pdf]
