# Supplementary material for: Environment and taxonomy shape the genomic signature of prokaryotic extremophiles
Source: Sci Rep. 2023 Sep 26;13:16105. doi: 10.1038/s41598-023-42518-y (PMC10522608; doi:10.1038/s41598-023-42518-y)
Supplement: Supplementary file 3 — Supplementary Information 3. [file 41598_2023_42518_MOESM3_ESM.pdf]

Unrooted Phylogenetic Tree Reconstructed from GTDB
Bacteria - Temperature Dataset

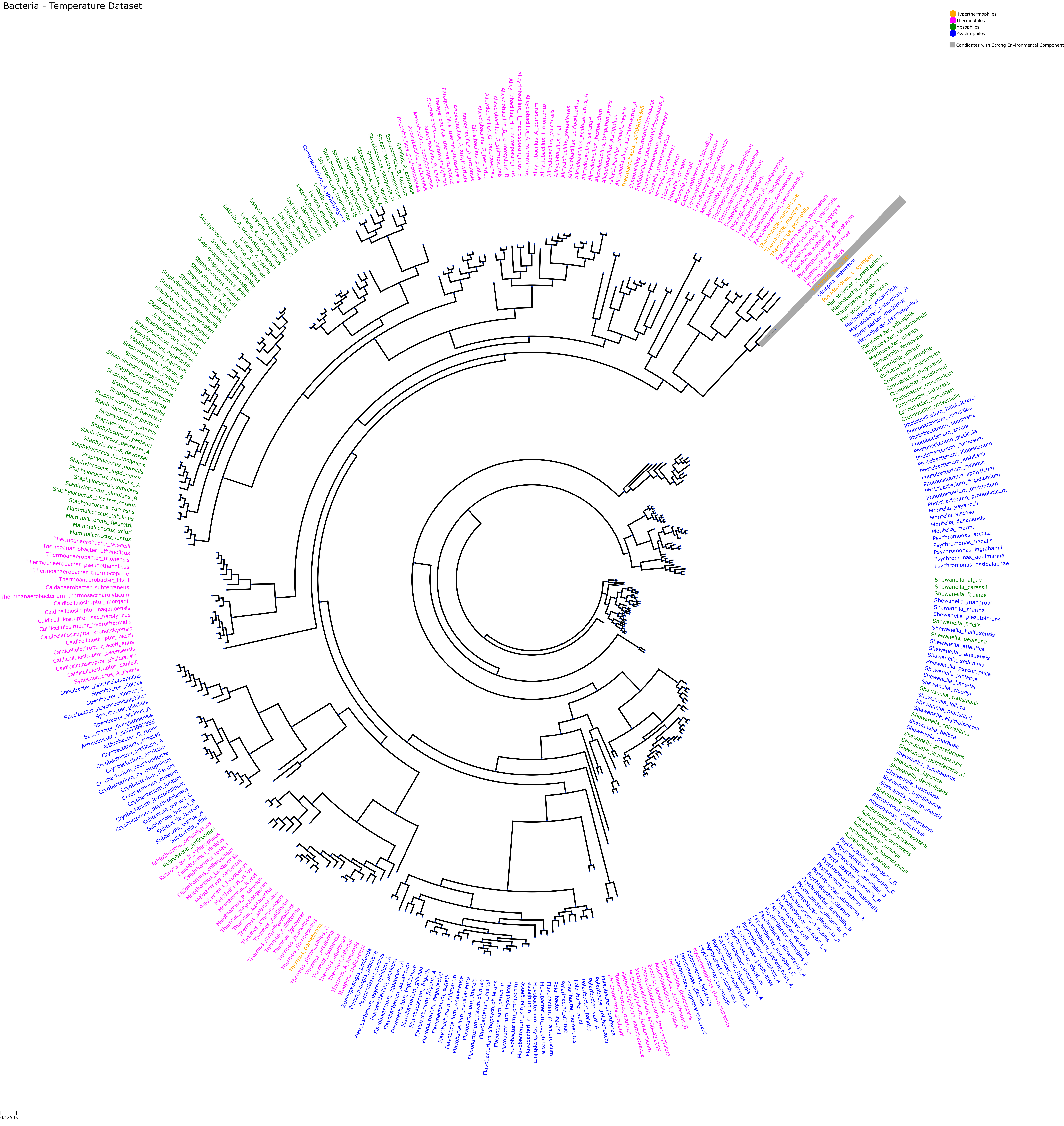

Unrooted Phylogenetic Tree Reconstructed from GTDB
Archaea - Temperature Dataset

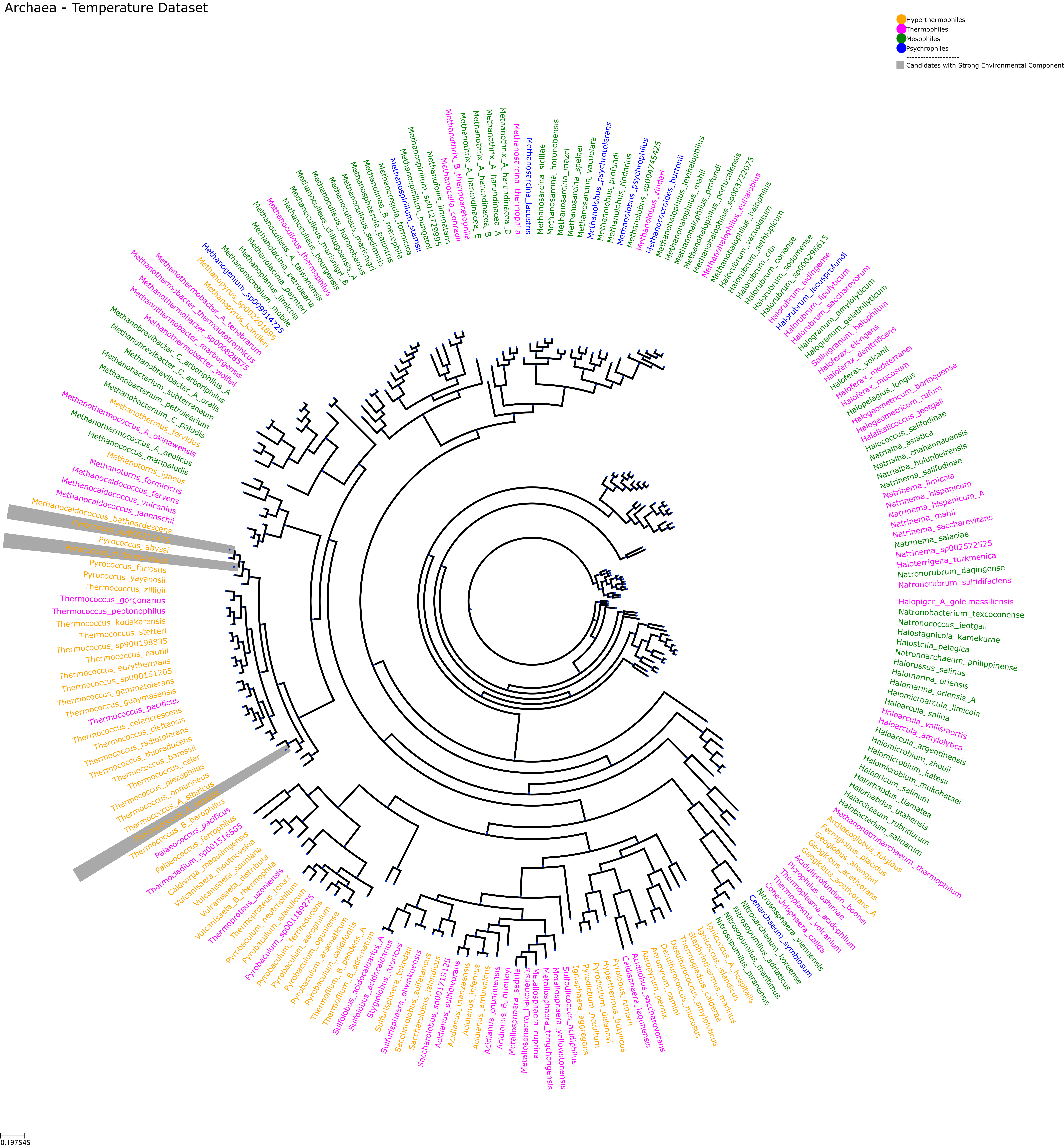

Unrooted Phylogenetic Tree Reconstructed from GTDB
Bacteria - pH Dataset

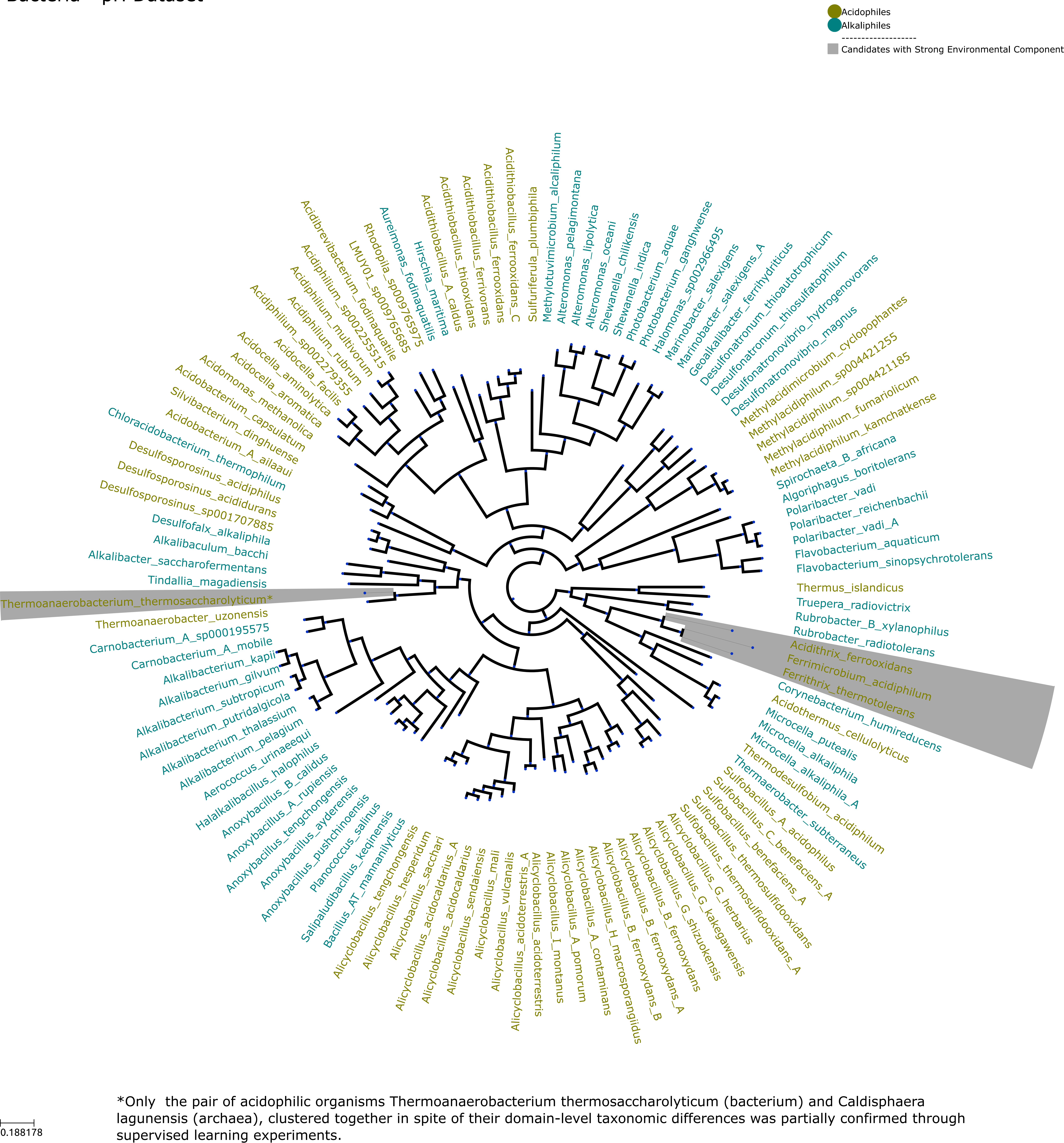

*Only the pair of acidophilic organisms Thermoanaerobacterium thermosaccharolyticum (bacterium) and Caldisphaera lagunensis (archaea), clustered together in spite of their domain-level taxonomic differences was partially confirmed through supervised learning experiments.

Unrooted Phylogenetic Tree Reconstructed from GTDB Archaea - pH Dataset

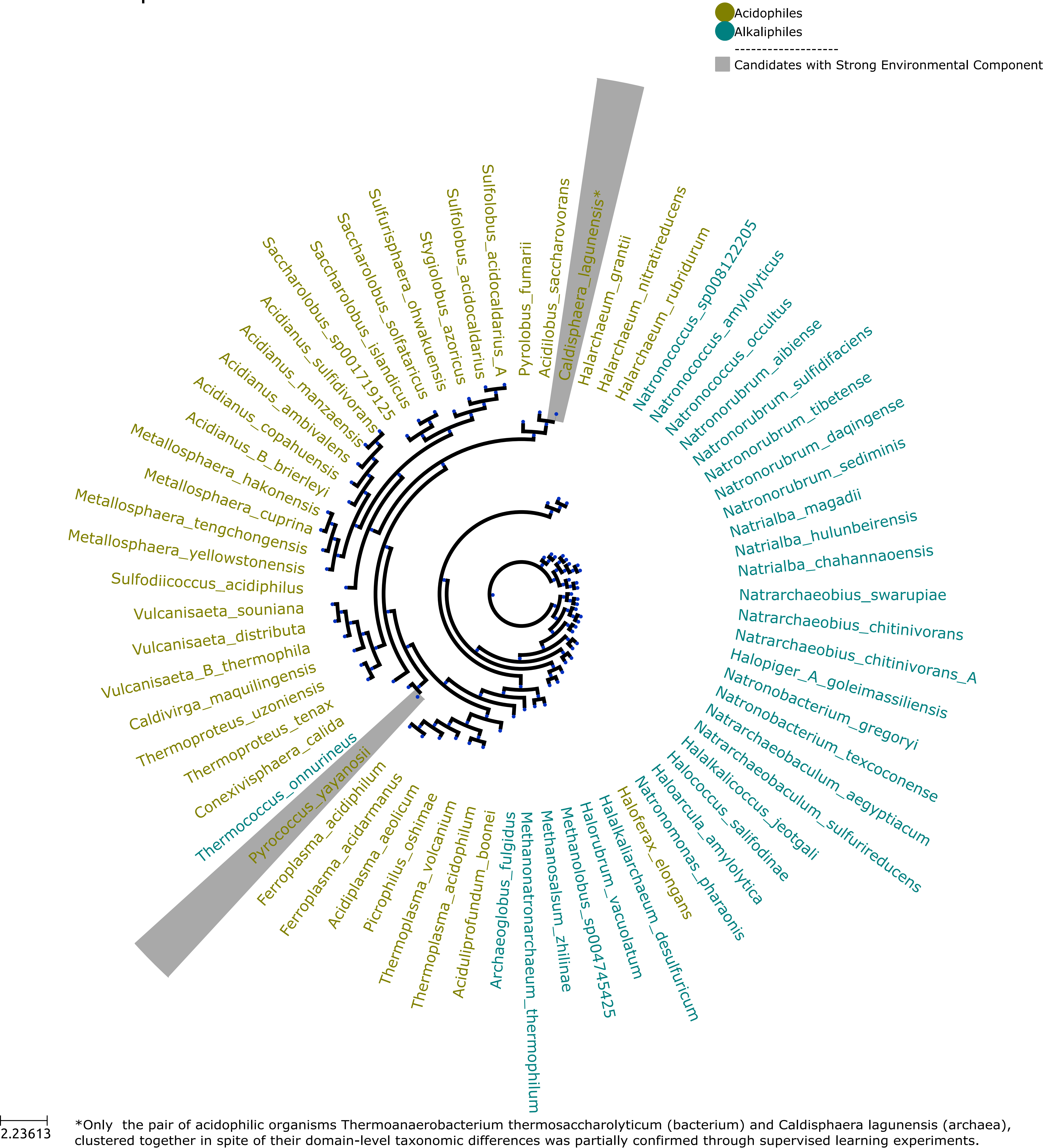

*Only the pair of acidophilic organisms Thermoanaerobacterium thermosaccharolyticum (bacterium) and Caldisphaera lagunensis (archaea), clustered together in spite of their domain-level taxonomic differences was partially confirmed through supervised learning experiments.
