# Supplementary material for: Environment and taxonomy shape the genomic signature of prokaryotic extremophiles
Source: Sci Rep. 2023 Sep 26;13:16105. doi: 10.1038/s41598-023-42518-y (PMC10522608; doi:10.1038/s41598-023-42518-y)

Supplementary Data S3
Single Nucleotide Composition - Temperature Dataset

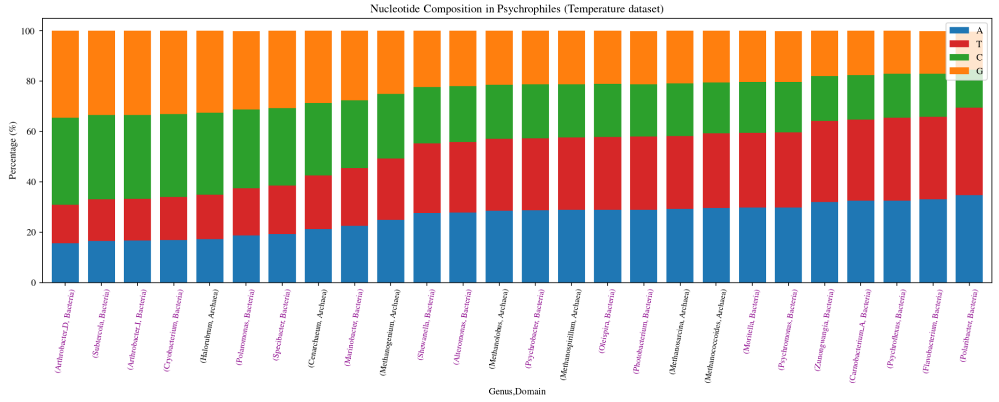

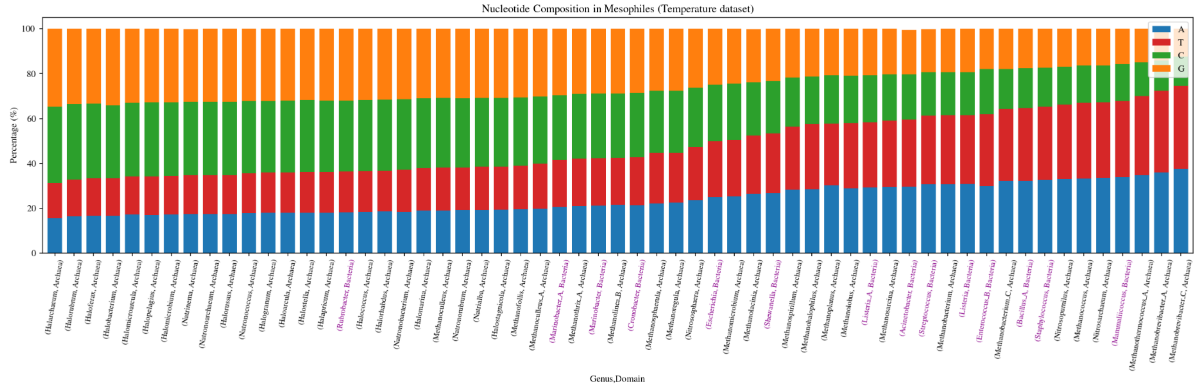

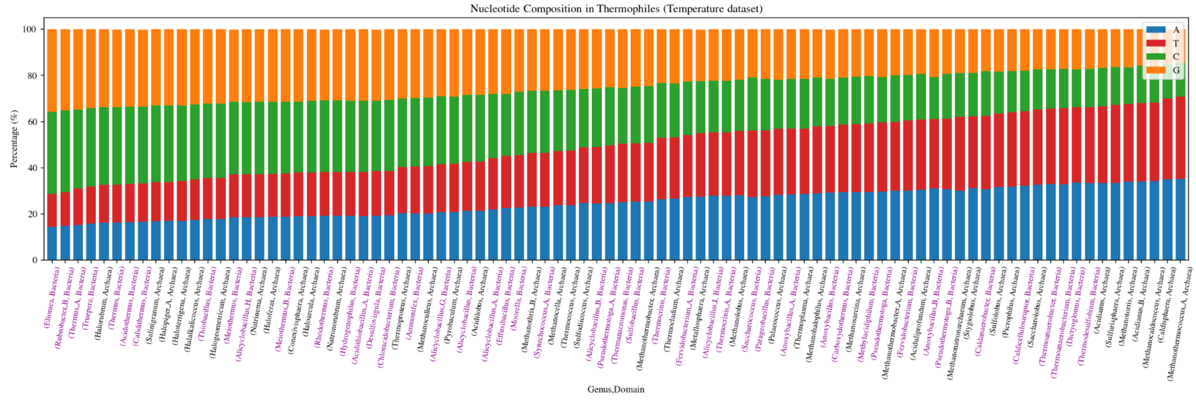

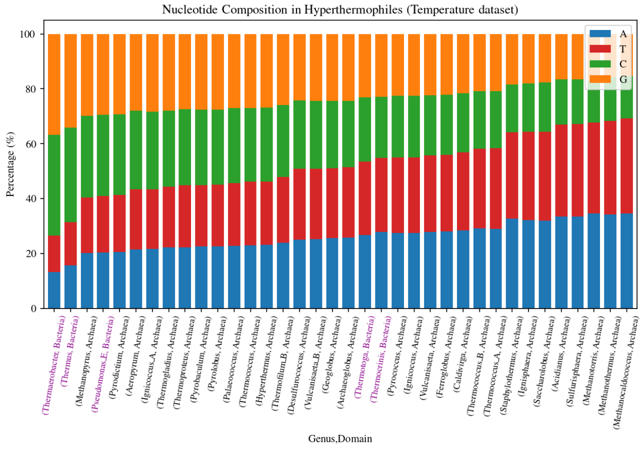

Single Nucleotide Composition - pH Dataset

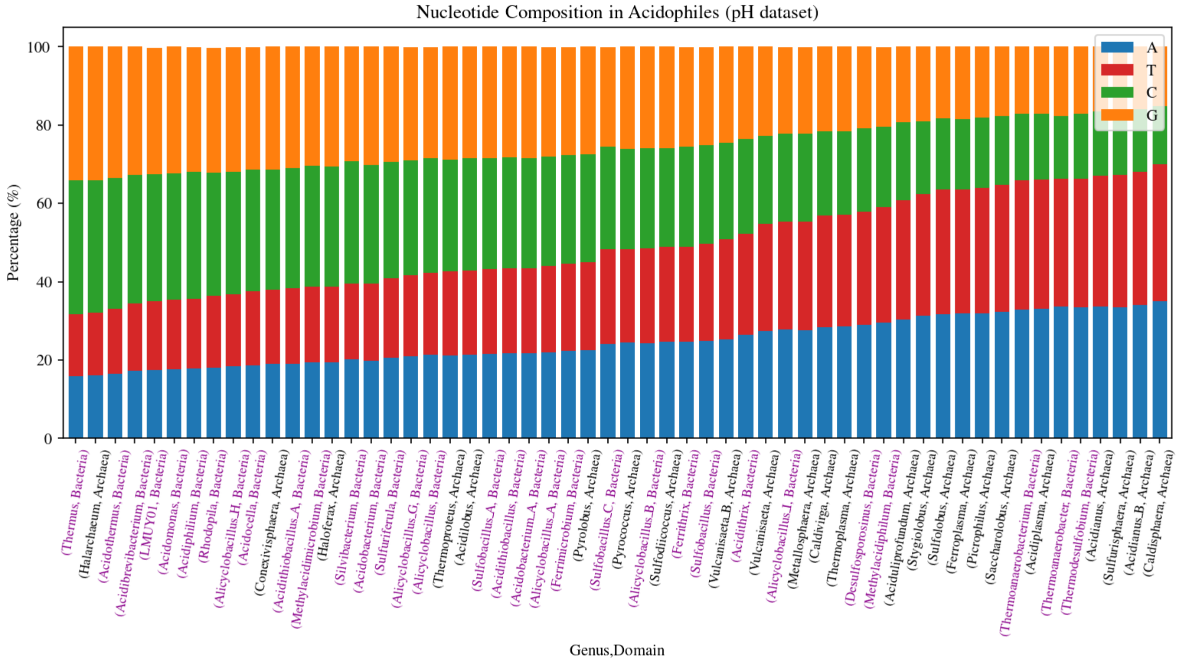

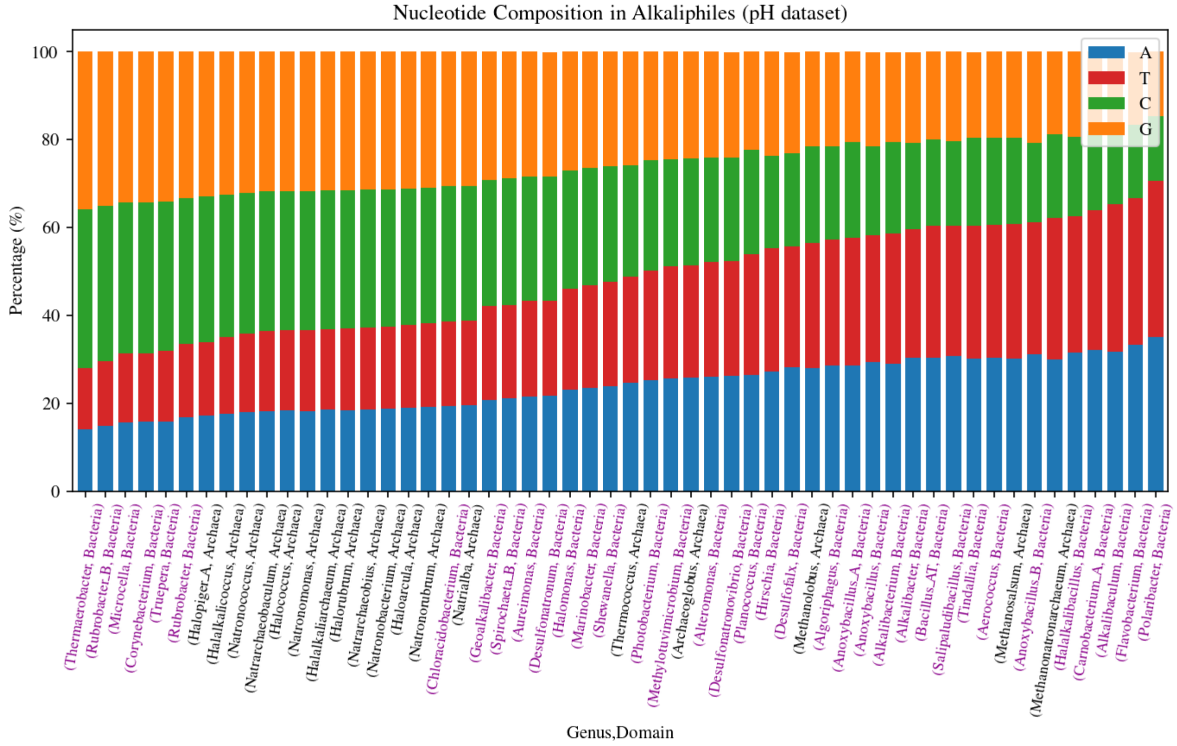

Supplement: Supplementary file 4 — Supplementary Information 4. [file 41598_2023_42518_MOESM4_ESM.pdf]
