# Supplementary material for: Environment and taxonomy shape the genomic signature of prokaryotic extremophiles
Source: Sci Rep. 2023 Sep 26;13:16105. doi: 10.1038/s41598-023-42518-y (PMC10522608; doi:10.1038/s41598-023-42518-y)
Supplement: Supplementary file 5 — Supplementary Information 5. [file 41598_2023_42518_MOESM5_ESM.pdf]

Supplementary Table S1 - Dataset Composition

Assembly	Temperature	pH	Genome Size	Domain	Phylum	Class	Order	Family	Genus	Species	NCBI tax ID	*NCBI Species*	Reference Link
GCA_009729015.1	Hyperthermophiles	Acidophiles	2252027	Archaea	Thermoproteota	Thermoproteia	Sulfolobales	Sulfolobaceae	Acidianus	*Acidianus ambivalens*	2283	*Acidianus ambivalens*	https://doi.org/10.1016/j.bbabio.2003.08.011 /
GCA_000632495.1	Thermophiles	Acidophiles	2454023	Archaea	Thermoproteota	Thermoproteia	Sulfolobales	Sulfolobaceae	Acidianus	*Acidianus copahuensis*	1160895	*Candidatus Acidianus copahuensis*	https://doi.org/10.1007/s00248-012-0129-4
GCA_009729545.1	Hyperthermophiles		2222414	Archaea	Thermoproteota	Thermoproteia	Sulfolobales	Sulfolobaceae	Acidianus	*Acidianus infernus*	12915	*Acidianus infernus So4a*	https://bacdive.dsmz.de/strain/16642
GCA_002116695.1	Hyperthermophiles	Acidophiles	2687463	Archaea	Thermoproteota	Thermoproteia	Sulfolobales	Sulfolobaceae	Acidianus	*Acidianus manzaensis*	282676	*Acidianus manzaensis*	https://doi.org/10.1007/s00284-006-0151-1
GCA_003201765.2	Thermophiles	Acidophiles	2265309	Archaea	Thermoproteota	Thermoproteia	Sulfolobales	Sulfolobaceae	Acidianus	*Acidianus sulfidivorans*	619593	*Acidianus sulfidivorans JP7*	https://doi.org/10.1099/ijs.0.64846-0
GCA_003201835.2	Thermophiles	Acidophiles	2947244	Archaea	Thermoproteota	Thermoproteia	Sulfolobales	Sulfolobaceae	Acidianus_B	*Acidianus_B brierleyi*	41673	*Acidianus brierleyi*	https://www.sciencedirect.com/science/article/pii/B9780123739445002819
GCA_003352165.1		Acidophiles	4037611	Bacteria	Proteobacteria	Alphaproteobacteria	Acetobacterales	Acetobacteraceae	Acidibrevibacterium	*Acidibrevibacterium fodinaquatile*	1969806	*Acidibrevibacterium fodinaquatile*	https://doi.org/10.1099/ijsem.0.003618
GCA_000144915.1	Thermophiles	Acidophiles	1496453	Archaea	Thermoproteota	Thermoproteia	Sulfolobales	Acidilobaceae	Acidilobus	*Acidilobus saccharovorans*	666510	*Acidilobus saccharovorans 345-15*	https://doi.org/10.1128%2FAEM.00599-10
GCA_000202835.1		Acidophiles	4214744	Bacteria	Proteobacteria	Alphaproteobacteria	Acetobacterales	Acetobacteraceae	Acidiphilium	*Acidiphilium multivorum*	349163	*Acidiphilium cryptum JF-5*	https://www.nite.go.jp/en/nbrc/genome/project/annotation/am1.html
GCA_900156265.1		Acidophiles	3976993	Bacteria	Proteobacteria	Alphaproteobacteria	Acetobacterales	Acetobacteraceae	Acidiphilium	*Acidiphilium rubrum*	1408418	*Acidiphilium angustum ATCC 35903*	https://doi.org/10.1128/mSystems.00867-20
GCA_902712915.1		Acidophiles	3851483	Bacteria	Proteobacteria	Alphaproteobacteria	Acetobacterales	Acetobacteraceae	Acidiphilium	*Acidiphilium sp002255515*	1970291	*Acidiphilium sp. 20-67-58*	https://doi.org/10.1128%2FmSystems.00867-20
GCA_002279355.1		Acidophiles	3069660	Bacteria	Proteobacteria	Alphaproteobacteria	Acetobacterales	Acetobacteraceae	Acidiphilium	*Acidiphilium sp002279355*	1970292	*Acidiphilium sp. 21-60-14*	https://doi.org/10.1128%2FmSystems.00867-20
GCA_001402945.1		Acidophiles	1778901	Archaea	Thermoplasmatota	Thermoplasmata	Thermoplasmatales	Thermoplasmataceae	Acidiplasma	*Acidiplasma aeolicum*	507754	*Acidiplasma aeolicum*	https://doi.org/10.1099/ijs.0.009639-0
GCA_018853935.1		Acidophiles	4623392	Bacteria	Proteobacteria	Gammaproteobacteria	Acidithiobacillales	Acidithiobacillaceae	Acidithiobacillus	*Acidithiobacillus ferrivorans*	160808	*Acidithiobacillus ferrivorans*	https://doi.org/10.1007/s00792-009-0282-y
GCA_000021485.1		Acidophiles	2982397	Bacteria	Proteobacteria	Gammaproteobacteria	Acidithiobacillales	Acidithiobacillaceae	Acidithiobacillus	*Acidithiobacillus ferrooxidans*	920	*Acidithiobacillus ferrooxidans*	https://doi.org/10.1074%2Fmcp.M700042-MCP200
GCA_018854495.1		Acidophiles	3011393	Bacteria	Proteobacteria	Gammaproteobacteria	Acidithiobacillales	Acidithiobacillaceae	Acidithiobacillus	*Acidithiobacillus ferrooxidans_C*	920	*Acidithiobacillus ferrooxidans*	https://doi.org/10.1074%2Fmcp.M700042-MCP200
GCA_009662475.1		Acidophiles	3415726	Bacteria	Proteobacteria	Gammaproteobacteria	Acidithiobacillales	Acidithiobacillaceae	Acidithiobacillus	*Acidithiobacillus thiooxidans*	930	*Acidithiobacillus thiooxidans*	https://doi.org/10.1128/jb.06281-11
GCA_000175575.2	Thermophiles	Acidophiles	2987045	Bacteria	Proteobacteria	Gammaproteobacteria	Acidithiobacillales	Acidithiobacillaceae	Acidithiobacillus_A	*Acidithiobacillus_A caldus*	637389	*Acidithiobacillus caldus ATCC 51756*	https://bacdive.dsmz.de/strain/126
GCA_000949295.1		Acidophiles	4019867	Bacteria	Actinobacteriota	Acidimicrobiia	Acidimicrobiales	Acidimicrobiaceae	Acidithrix	*Acidithrix ferrooxidans*	1280514	*Acidithrix ferrooxidans*	https://doi.org/10.1016/j.resmic.2015.01.003
GCA_000022565.1		Acidophiles	4127356	Bacteria	Acidobacteriota	Acidobacteriae	Acidobacteriales	Acidobacteriaceae	Acidobacterium	*Acidobacterium capsulatum*	240015	*Acidobacterium capsulatum ATCC 51196*	https://doi.org/10.1128/AEM.02294-08
GCA_000688455.1		Acidophiles	3686523	Bacteria	Acidobacteriota	Acidobacteriae	Acidobacteriales	Acidobacteriaceae	Acidobacterium_A	*Acidobacterium_A ailaaui*	1382359	*Pseudacidobacterium ailaaui*	https://doi.org/10.1099/ijsem.0.005415
GCA_900129125.1		Acidophiles	3964820	Bacteria	Proteobacteria	Alphaproteobacteria	Acetobacterales	Acetobacteraceae	Acidocella	*Acidocella aminolytica*	1120923	*Acidocella aminolytica 101 = DSM 11237*	https://doi.org/10.1016/S0723-2020(11)80453-4
GCA_014201825.1		Acidophiles	2877351	Bacteria	Proteobacteria	Alphaproteobacteria	Acetobacterales	Acetobacteraceae	Acidocella	*Acidocella aromatica*	1303579	*Acidocella aromatica*	https://doi.org/10.1007/s00792-013-0566-0
GCA_000687875.1		Acidophiles	3403931	Bacteria	Proteobacteria	Alphaproteobacteria	Acetobacterales	Acetobacteraceae	Acidocella	*Acidocella facilis*	1214225	*Acidocella sp. MX-AZ02*	http://dx.doi.org/10.1264/jsme2.2002.98
GCA_004346035.1		Acidophiles	3681251	Bacteria	Proteobacteria	Alphaproteobacteria	Acetobacterales	Acetobacteraceae	Acidomonas	*Acidomonas methanolica*	437	*Acidomonas methanolica*	https://doi.org/10.1099/00207713-39-1-50
GCA_000015025.1	Thermophiles	Acidophiles	2443540	Bacteria	Actinobacteriota	Actinomycetia	Acidothermales	Acidothermaceae	Acidothermus	*Acidothermus cellulolyticus*	351607	*Acidothermus cellulolyticus 11B*	https://www.atcc.org/products/43068
GCA_000025665.1	Thermophiles	Acidophiles	1486778	Archaea	Thermoplasmatota	Thermoplasmata	Aciduliprofundales	Aciduliprofundaceae	Aciduliprofundum	*Aciduliprofundum boonei*	439481	*Aciduliprofundum boonei*	https://doi.org/10.1007%2Fs00792-007-0111-0
GCA_009759685.1	Mesophiles		3990388	Bacteria	Proteobacteria	Gammaproteobacteria	Pseudomonadales	Moraxellaceae	Acinetobacter	*Acinetobacter baumannii*	509170	*Acinetobacter baumannii SDF*	https://doi.org/10.2166/wst.2018.409
GCA_000369065.1	Mesophiles		3513082	Bacteria	Proteobacteria	Gammaproteobacteria	Pseudomonadales	Moraxellaceae	Acinetobacter	*Acinetobacter haemolyticus*	707232	*Acinetobacter haemolyticus ATCC 19194*	https://bacdive.dsmz.de/strain/137522
GCA_000196795.1	Mesophiles		4152543	Bacteria	Proteobacteria	Gammaproteobacteria	Pseudomonadales	Moraxellaceae	Acinetobacter	*Acinetobacter oleivorans*	436717	*Acinetobacter oleivorans DR1*	https://bacdive.dsmz.de/strain/157136
GCA_000368025.1	Mesophiles		2912642	Bacteria	Proteobacteria	Gammaproteobacteria	Pseudomonadales	Moraxellaceae	Acinetobacter	*Acinetobacter parvus*	981333	*Acinetobacter parvus DSM 16617 = CIP 108168*	https://bacdive.dsmz.de/strain/8139
GCA_006757745.1	Mesophiles		3433938	Bacteria	Proteobacteria	Gammaproteobacteria	Pseudomonadales	Moraxellaceae	Acinetobacter	*Acinetobacter radioresistens*	575589	*Acinetobacter radioresistens SH164*	https://bacdive.dsmz.de/strain/155470
GCA_000368825.1	Mesophiles		3535739	Bacteria	Proteobacteria	Gammaproteobacteria	Pseudomonadales	Moraxellaceae	Acinetobacter	*Acinetobacter ursingii*	981336	*Acinetobacter ursingii DSM 16037 = CIP 107286*	https://bacdive.dsmz.de/strain/155369
GCA_001543205.1		Alkaliphiles	2013339	Bacteria	Firmicutes	Bacilli	Lactobacillales	Aerococcaceae	Aerococcus	*Aerococcus urinaeequi*	1120952	*Aerococcus urinaeequi DSM 20341 = CCUG 28094*	https://bacdive.dsmz.de/strain/222
GCA_000591035.1	Hyperthermophiles		1595994	Archaea	Thermoproteota	Thermoproteia	Sulfolobales	Acidilobaceae	Aeropyrum	*Aeropyrum camini*	1198449	*Aeropyrum camini SY1 = JCM 12091*	https://doi.org/10.1099/ijs.0.02826-0
GCA_000011125.1	Hyperthermophiles		1669696	Archaea	Thermoproteota	Thermoproteia	Sulfolobales	Acidilobaceae	Aeropyrum	*Aeropyrum pernix*	56636	*Aeropyrum pernix*	https://doi.org/10.1099/00207713-46-4-1070
GCA_900108085.1		Alkaliphiles	4834448	Bacteria	Bacteroidota	Bacteroidia	Cytophagales	Cyclobacteriaceae	Algoriphagus	*Algoriphagus boritolerans*	1120964	*Algoriphagus boritolerans DSM 17298 = JCM 18970*	https://bacdive.dsmz.de/strain/17702
GCA_001544355.1	Thermophiles		3865262	Bacteria	Firmicutes	Bacilli	Alicyclobacillales	Alicyclobacillaceae	Alicyclobacillus	*Alicyclobacillus acidiphilus*	1255277	*Alicyclobacillus acidiphilus NBRC 100859*	https://doi.org/10.1016/B978-0-12-802230-6.00016-3
GCA_000024285.1	Thermophiles	Acidophiles	3205686	Bacteria	Firmicutes	Bacilli	Alicyclobacillales	Alicyclobacillaceae	Alicyclobacillus	*Alicyclobacillus acidocaldarius*	521098	*Alicyclobacillus acidocaldarius subsp. acidocaldarius DSM 446*	https://doi.org/10.2174/092986608785849209
GCA_000219875.1	Thermophiles	Acidophiles	3124048	Bacteria	Firmicutes	Bacilli	Alicyclobacillales	Alicyclobacillaceae	Alicyclobacillus	*Alicyclobacillus acidocaldarius_A*	1048834	*Alicyclobacillus acidocaldarius subsp. acidocaldarius Tc-4-1*	https://doi.org/10.1016/j.fm.2008.07.008
GCA_000444055.1	Thermophiles	Acidophiles	4063548	Bacteria	Firmicutes	Bacilli	Alicyclobacillales	Alicyclobacillaceae	Alicyclobacillus	*Alicyclobacillus acidoterrestris*	1450	*Alicyclobacillus acidoterrestris*	https://doi.org/10.1016/B978-0-12-384730-0.00404-3
GCA_007991715.1	Thermophiles	Acidophiles	4033884	Bacteria	Firmicutes	Bacilli	Alicyclobacillales	Alicyclobacillaceae	Alicyclobacillus	*Alicyclobacillus acidoterrestris_A*	1450	*Alicyclobacillus acidoterrestris*	https://doi.org/10.1016/B978-0-12-384730-0.00404-3
GCA_900107035.1	Thermophiles	Acidophiles	2859702	Bacteria	Firmicutes	Bacilli	Alicyclobacillales	Alicyclobacillaceae	Alicyclobacillus	*Alicyclobacillus hesperidum*	89784	*Alicyclobacillus hesperidum*	https://doi.org/10.1128%2FJB.01612-12
GCA_001570745.1	Thermophiles	Acidophiles	2786970	Bacteria	Firmicutes	Bacilli	Alicyclobacillales	Alicyclobacillaceae	Alicyclobacillus	*Alicyclobacillus mali*	1314748	*Alicyclobacillus mali NBRC 102425*	https://doi.org/10.3389/fmicb.2021.639697
GCA_004366795.1	Thermophiles	Acidophiles	2946319	Bacteria	Firmicutes	Bacilli	Alicyclobacillales	Alicyclobacillaceae	Alicyclobacillus	*Alicyclobacillus sacchari*	392010	*Alicyclobacillus sacchari*	https://doi.org/10.1099/ijs.0.64692-0
GCA_001552675.1	Thermophiles	Acidophiles	2793850	Bacteria	Firmicutes	Bacilli	Alicyclobacillales	Alicyclobacillaceae	Alicyclobacillus	*Alicyclobacillus sendaiensis*	1220572	*Alicyclobacillus sendaiensis NBRC 100866*	http://dx.doi.org/10.1099/ijs.0.02409-0
GCA_001447355.1	Thermophiles	Acidophiles	2809442	Bacteria	Firmicutes	Bacilli	Alicyclobacillales	Alicyclobacillaceae	Alicyclobacillus	*Alicyclobacillus tengchongensis*	368812	*Alicyclobacillus tengchongensis*	https://doi.org/10.1007/s12275-014-3625-z
GCA_900156755.1	Thermophiles	Acidophiles	2994480	Bacteria	Firmicutes	Bacilli	Alicyclobacillales	Alicyclobacillaceae	Alicyclobacillus	*Alicyclobacillus vulcanalis*	252246	*Alicyclobacillus vulcanalis*	https://doi.org/10.1099/ijs.0.03012-0
GCA_000429525.1	Thermophiles	Acidophiles	3272318	Bacteria	Firmicutes	Bacilli	Alicyclobacillales	Alicyclobacillaceae	Alicyclobacillus_A	*Alicyclobacillus_A contaminans*	1120971	*Alicyclobacillus contaminans DSM 17975*	https://doi.org/10.3390/microorganisms3040625
GCA_000472905.1	Thermophiles	Acidophiles	3398558	Bacteria	Firmicutes	Bacilli	Alicyclobacillales	Alicyclobacillaceae	Alicyclobacillus_A	*Alicyclobacillus_A pomorum*	1111479	*Alicyclobacillus pomorum DSM 14955*	https://bacmedia.dsmz.de/medium/402?ccno=DSM+14955 / https://bacdive.dsmz.de/strain/424
GCA_001399675.1		Acidophiles	5044184	Bacteria	Firmicutes	Bacilli	Alicyclobacillales	Alicyclobacillaceae	Alicyclobacillus_B	*Alicyclobacillus_B ferrooxydans*	471514	*Alicyclobacillus ferrooxydans*	http://dx.doi.org/10.1099/ijs.0.2008/000562-0
GCA_017298655.1		Acidophiles	6027356	Bacteria	Firmicutes	Bacilli	Alicyclobacillales	Alicyclobacillaceae	Alicyclobacillus_B	*Alicyclobacillus_B ferrooxydans_A*	471514	*Alicyclobacillus ferrooxydans (Alicyclobacillus curvatus)*	https://doi.org/10.1099/ijsem.0.005285
GCA_017298635.1	Thermophiles	Acidophiles	5293537	Bacteria	Firmicutes	Bacilli	Alicyclobacillales	Alicyclobacillaceae	Alicyclobacillus_B	*Alicyclobacillus_B ferrooxydans_B*	471514	*Alicyclobacillus ferrooxydans (Alicyclobacillus mengziensis)*	https://doi.org/10.1093%2Fg3journal%2Fjkac225
GCA_000430585.1	Thermophiles	Acidophiles	3310509	Bacteria	Firmicutes	Bacilli	Alicyclobacillales	Alicyclobacillaceae	Alicyclobacillus_G	*Alicyclobacillus_G herbarius*	1120972	*Alicyclobacillus herbarius DSM 13609*	https://doi.org/10.1099/00207713-52-1-109
GCA_001552655.1	Thermophiles	Acidophiles	3421583	Bacteria	Firmicutes	Bacilli	Alicyclobacillales	Alicyclobacillaceae	Alicyclobacillus_G	*Alicyclobacillus_G kakegawensis*	1220571	*Alicyclobacillus kakegawensis NBRC 103104*	https://doi.org/10.1099/ijs.0.64692-0
GCA_001552255.1	Thermophiles	Acidophiles	3628929	Bacteria	Firmicutes	Bacilli	Alicyclobacillales	Alicyclobacillaceae	Alicyclobacillus_G	*Alicyclobacillus_G shizuokensis*	1220573	*Alicyclobacillus shizuokensis NBRC 103103*	https://doi.org/10.1099/ijs.0.64692-0

GCA_900116805.1	Thermophiles	Acidophiles	3789420	Bacteria	Firmicutes	Bacilli	Alicyclobacillales	Alicyclobacillaceae	Alicyclobacillus_H	*Alicyclobacillus_H macrosporangiidus*	392015	*Alicyclobacillus macrosporangiidus*	https://doi.org/10.1099/ijs.0.64692-0
GCA_000702485.1	Thermophiles		4091649	Bacteria	Firmicutes	Bacilli	Alicyclobacillales	Alicyclobacillaceae	Alicyclobacillus_H	*Alicyclobacillus_H macrosporangiidus_B*	392015	*Alicyclobacillus macrosporangiidus*	https://doi.org/10.3390%2Fmicroorganisms10030656
GCA_900142255.1	Thermophiles	Acidophiles	3045912	Bacteria	Firmicutes	Bacilli	Alicyclobacillales	Alicyclobacillaceae	Alicyclobacillus_I	*Alicyclobacillus_I montanus*	1830138	*Alicyclobacillus montanus*	https://bacdive.dsmz.de/strain/158721
GCA_900128885.1		Alkaliphiles	2331365	Bacteria	Firmicutes_A	Clostridia	Eubacteriales	Alkalibacteraceae	Alkalibacter	*Alkalibacter saccharofermentans*	1120975	*Alkalibacter saccharofermentans DSM 14828*	https://doi.org/10.1007/s00792-004-0390-7
GCA_900109085.1		Alkaliphiles	2181898	Bacteria	Firmicutes	Bacilli	Lactobacillales	Carnobacteriaceae	Alkalibacterium	*Alkalibacterium gilvum*	1130080	*Alkalibacterium gilvum*	https://doi.org/10.1099/ijs.0.042556-0
GCA_007988865.1		Alkaliphiles	1991267	Bacteria	Firmicutes	Bacilli	Lactobacillales	Carnobacteriaceae	Alkalibacterium	*Alkalibacterium kapii*	426704	*Alkalibacterium kapii*	https://bacdive.dsmz.de/strain/2243
GCA_900109325.1		Alkaliphiles	2638902	Bacteria	Firmicutes	Bacilli	Lactobacillales	Carnobacteriaceae	Alkalibacterium	*Alkalibacterium pelagium*	426702	*Alkalibacterium pelagium*	https://bacdive.dsmz.de/strain/2246
GCA_900109825.1		Alkaliphiles	2600402	Bacteria	Firmicutes	Bacilli	Lactobacillales	Carnobacteriaceae	Alkalibacterium	*Alkalibacterium putridalgicola*	426703	*Alkalibacterium putridalgicola*	https://doi.org/10.1099/ijs.0.65602-0
GCA_900112455.1		Alkaliphiles	2491452	Bacteria	Firmicutes	Bacilli	Lactobacillales	Carnobacteriaceae	Alkalibacterium	*Alkalibacterium subtropicum*	753702	*Alkalibacterium subtropicum*	https://doi.org/10.1099/ijs.0.027953-0
GCA_900101165.1		Alkaliphiles	2364962	Bacteria	Firmicutes	Bacilli	Lactobacillales	Carnobacteriaceae	Alkalibacterium	*Alkalibacterium thalassium*	426701	*Alkalibacterium thalassium*	https://doi.org/10.1099/ijs.0.65602-0
GCA_003317055.1		Alkaliphiles	3098941	Bacteria	Firmicutes_A	Clostridia	Eubacteriales	Alkalibacteraceae	Alkalibaculum	*Alkalibaculum bacchi*	645887	*Alkalibaculum bacchi*	http://dx.doi.org/10.1099/ijs.0.018507-0
GCA_001758465.1		Alkaliphiles	5018451	Bacteria	Proteobacteria	Gammaproteobacteria	Enterobacterales	Alteromonadaceae	Alteromonas	*Alteromonas lipolytica*	1856405	*Alteromonas lipolytica*	https://bacdive.dsmz.de/strain/133345
GCA_000020585.3	Psychrophiles		4480937	Bacteria	Proteobacteria	Gammaproteobacteria	Enterobacterales	Alteromonadaceae	Alteromonas	*Alteromonas mediterranea*	1300253	*Alteromonas mediterranea 615*	https://bacdive.dsmz.de/strain/23563
GCA_003731635.1		Alkaliphiles	4987953	Bacteria	Proteobacteria	Gammaproteobacteria	Enterobacterales	Alteromonadaceae	Alteromonas	*Alteromonas oceani*	2071609	*Alteromonas oceani*	https://bacdive.dsmz.de/strain/158616#ref65338
GCA_002499975.2		Alkaliphiles	4314189	Bacteria	Proteobacteria	Gammaproteobacteria	Enterobacterales	Alteromonadaceae	Alteromonas	*Alteromonas pelagimontana*	1858656	*Alteromonas pelagimontana*	https://bacdive.dsmz.de/strain/140746
GCA_001562115.1	Psychrophiles		4904192	Bacteria	Proteobacteria	Gammaproteobacteria	Enterobacterales	Alteromonadaceae	Alteromonas	*Alteromonas stellipolaris*	233316	*Alteromonas stellipolaris*	https://bacdive.dsmz.de/strain/447#ref6058
GCA_000024605.1	Thermophiles		2157067	Bacteria	Firmicutes_B	Desulfotomaculia	Ammonifexales	Ammonificaceae	Ammonifex	*Ammonifex degensii*	429009	*Ammonifex degensii KC4*	https://bacdive.dsmz.de/strain/16789
GCA_003368535.1	Thermophiles		2111834	Bacteria	Firmicutes_B	Desulfotomaculia	Ammonifexales	Ammonificaceae	Ammonifex	*Ammonifex thiophilus*	444093	*Ammonifex thiophilus*	https://bacdive.dsmz.de/strain/16790
GCA_000833605.1	Thermophiles	Alkaliphiles	2832347	Bacteria	Firmicutes	Bacilli	Bacillales	Anoxybacillaceae	Anoxybacillus	*Anoxybacillus ayderensis*	265546	*Anoxybacillus ayderensis*	https://pubmed.ncbi.nlm.nih.gov/15388701/
GCA_900111795.1	Thermophiles	Alkaliphiles	2625421	Bacteria	Firmicutes	Bacilli	Bacillales	Anoxybacillaceae	Anoxybacillus	*Anoxybacillus pushchinoensis*	150248	*Anoxybacillus pushchinoensis*	https://doi.org/10.1099/00207713-50-6-2109
GCA_014201585.1	Thermophiles	Alkaliphiles	2727467	Bacteria	Firmicutes	Bacilli	Bacillales	Anoxybacillaceae	Anoxybacillus	*Anoxybacillus tengchongensis*	576944	*Anoxybacillus tengchongensis*	https://doi.org/10.1099/ijs.0.020834-0
GCA_001634285.1	Thermophiles		3158269	Bacteria	Firmicutes	Bacilli	Bacillales	Anoxybacillaceae	Anoxybacillus_A	*Anoxybacillus_A amylolyticus*	294699	*Anoxybacillus amylolyticus*	https://doi.org/10.1016/j.syapm.2005.10.003
GCA_014196195.1	Thermophiles	Alkaliphiles	3705654	Bacteria	Firmicutes	Bacilli	Bacillales	Anoxybacillaceae	Anoxybacillus_A	*Anoxybacillus_A rupiensis*	1895648	*Anoxybacillus sp. UARK-01*	https://doi.org/10.1007/s00284-017-1239-5
GCA_013760845.1	Thermophiles	Alkaliphiles	3405528	Bacteria	Firmicutes	Bacilli	Bacillales	Anoxybacillaceae	Anoxybacillus_B	*Anoxybacillus_B calidus*	575178	*Anoxybacillus calidus*	https://pubmed.ncbi.nlm.nih.gov/24052627/
GCA_000008665.1	Hyperthermophiles	Alkaliphiles	2178400	Archaea	Halobacteriota	Archaeoglobi	Archaeoglobales	Archaeoglobaceae	Archaeoglobus	*Archaeoglobus fulgidus*	2234	*Archaeoglobus fulgidus*	https://doi.org/10.1007/s13205-019-1693-9
GCA_002954225.1	Psychrophiles		3655669	Bacteria	Actinobacteriota	Actinomycetia	Actinomycetales	Micrococcaceae	Arthrobacter_D	*Arthrobacter_D ruber*	1258893	*Arthrobacter ruber*	https://doi.org/10.1099/ijsem.0.002719
GCA_003097355.1	Psychrophiles		4708612	Bacteria	Actinobacteriota	Actinomycetia	Actinomycetales	Micrococcaceae	Arthrobacter_I	*Arthrobacter_I sp003097355*	2703675	*Arthrobacter sp. H-02-3*	https://doi.org/10.1186/s40168-021-01084-z
GCA_008369445.1		Alkaliphiles	3936980	Bacteria	Proteobacteria	Alphaproteobacteria	Rhizobiales	Rhizobiaceae	Aureimonas	*Aureimonas fodinaquatilis*	2565783	*Aureimonas fodinaquatilis*	https://doi.org/10.1007/s00203-020-01988-8
GCA_000742895.1	Mesophiles		5506189	Bacteria	Firmicutes	Bacilli	Bacillales	Bacillaceae_G	Bacillus_A	*Bacillus_A anthracis*	572264	*Bacillus cereus 03BB102*	https://edis.ifas.ufl.edu/publication/FS269
GCA_000615945.1		Alkaliphiles	4534596	Bacteria	Firmicutes	Bacilli	Caldalkalibacillales	JCM-10596	Bacillus_AT	*Bacillus_AT mannanilyticus*	1236954	*Caldalkalibacillus mannanilyticus JCM 10596*	https://doi.org/10.1007/s00203-022-02789-x
GCA_004345675.1	Thermophiles		2575920	Bacteria	Firmicutes_A	Thermoanaerobacteria	Thermoanaerobacterales	Thermoanaerobacteraceae	Caldanaerobacter	*Caldanaerobacter subterraneus*	911092	*Caldanaerobacter subterraneus*	https://doi.org/10.1128%2FAEM.05190-11
GCA_000421725.1	Thermophiles		2589957	Bacteria	Firmicutes_A	Thermoanaerobacteria	Caldicellulosiruptorales	Caldicellulosiruptoraceae	Caldicellulosiruptor	*Caldicellulosiruptor acetigenus*	1121259	*Caldicellulosiruptor acetigenus DSM 7040*	https://doi.org/10.1099/ijs.0.63723-0
GCA_000022325.1	Thermophiles		2931662	Bacteria	Firmicutes_A	Thermoanaerobacteria	Caldicellulosiruptorales	Caldicellulosiruptoraceae	Caldicellulosiruptor	*Caldicellulosiruptor bescii*	31899	*Caldicellulosiruptor bescii*	https://doi.org/10.1099/ijs.0.017731-0
GCA_000955725.1	Thermophiles		2834482	Bacteria	Firmicutes_A	Thermoanaerobacteria	Caldicellulosiruptorales	Caldicellulosiruptoraceae	Caldicellulosiruptor	*Caldicellulosiruptor danielii*	1387557	*Caldicellulosiruptor danielii*	https://doi.org/10.1128/aem.02694-17
GCA_000166355.1	Thermophiles		2770676	Bacteria	Firmicutes_A	Thermoanaerobacteria	Caldicellulosiruptorales	Caldicellulosiruptoraceae	Caldicellulosiruptor	*Caldicellulosiruptor hydrothermalis*	632292	*Caldicellulosiruptor hydrothermalis 108*	https://www.microbiologyresearch.org/content/journal/ijsem/10.1099/ijs.0.65236-0
GCA_000166775.1	Thermophiles		2843785	Bacteria	Firmicutes_A	Thermoanaerobacteria	Caldicellulosiruptorales	Caldicellulosiruptoraceae	Caldicellulosiruptor	*Caldicellulosiruptor kronotskyensis*	632348	*Caldicellulosiruptor kronotskyensis 2002*	https://doi.org/10.1099/ijs.0.65236-0
GCA_000955745.1	Thermophiles		2488483	Bacteria	Firmicutes_A	Thermoanaerobacteria	Caldicellulosiruptorales	Caldicellulosiruptoraceae	Caldicellulosiruptor	*Caldicellulosiruptor morganii*	1387555	*Caldicellulosiruptor morganii*	https://doi.org/10.1128/genomeA.00440-15
GCA_000955735.1	Thermophiles		2514985	Bacteria	Firmicutes_A	Thermoanaerobacteria	Caldicellulosiruptorales	Caldicellulosiruptoraceae	Caldicellulosiruptor	*Caldicellulosiruptor naganoensis*	1387569	*Caldicellulosiruptor naganoensis NA10*	https://doi.org/10.1128/mra.01292-22
GCA_000145215.1	Thermophiles		2532343	Bacteria	Firmicutes_A	Thermoanaerobacteria	Caldicellulosiruptorales	Caldicellulosiruptoraceae	Caldicellulosiruptor	*Caldicellulosiruptor obsidiansis*	608506	*Caldicellulosiruptor obsidiansis OB47*	https://doi.org/10.1128/aem.01903-09
GCA_000166335.1	Thermophiles		2428903	Bacteria	Firmicutes_A	Thermoanaerobacteria	Caldicellulosiruptorales	Caldicellulosiruptoraceae	Caldicellulosiruptor	*Caldicellulosiruptor owensensis*	632518	*Caldicellulosiruptor owensensis OL*	https://doi.org/10.1128/jb.01515-10
GCA_000016545.1	Thermophiles		2970275	Bacteria	Firmicutes_A	Thermoanaerobacteria	Caldicellulosiruptorales	Caldicellulosiruptoraceae	Caldicellulosiruptor	*Caldicellulosiruptor saccharolyticus*	351627	*Caldicellulosiruptor saccharolyticus DSM 8903*	https://doi.org/10.1007/s00253-006-0783-x
GCA_000317795.1	Thermophiles	Acidophiles	1546846	Archaea	Thermoproteota	Thermoproteia	Sulfolobales	Acidilobaceae	Caldisphaera	*Caldisphaera lagunensis*	1056495	*Caldisphaera lagunensis*	https://doi.org/10.1099/ijs.0.02580-0
GCA_000018305.1	Hyperthermophiles	Acidophiles	2077567	Archaea	Thermoproteota	Thermoproteia	Thermoproteales	Thermocladiaceae	Caldivirga	*Caldivirga maquilingensis*	397948	*Caldivirga maquilingensis IC-167*	https://doi.org/10.1099/00207713-49-3-1157
GCA_000430045.1	Thermophiles		4688964	Bacteria	Deinococcota	Deinococci	Deinococcales	Thermaceae	Calidithermus	*Calidithermus chliarophilus*	926560	*Calidithermus chliarophilus DSM 9957*	https://www.microbiologyresearch.org/content/journal/ijsem/10.1099/ijsem.0.003270
GCA_003574095.1	Thermophiles		3675477	Bacteria	Deinococcota	Deinococci	Deinococcales	Thermaceae	Calidithermus	*Calidithermus roseus*	1644118	*Calidithermus roseus*	https://www.microbiologyresearch.org/content/journal/ijsem/10.1099/ijsem.0.003270
GCA_000373205.1	Thermophiles		3190628	Bacteria	Deinococcota	Deinococci	Deinococcales	Thermaceae	Calidithermus	*Calidithermus timidus*	1122223	*Calidithermus timidus DSM 17022*	https://www.microbiologyresearch.org/content/journal/ijsem/10.1099/ijsem.0.003270
GCA_001950325.1	Thermophiles		2386596	Bacteria	Firmicutes_B	Z-2901	Carboxydothermales	Carboxydothermaceae	Carboxydothermus	*Carboxydothermus islandicus*	661089	*Carboxydothermus islandicus*	https://doi.org/10.1099/ijs.0.030288-0
GCA_001950255.1	Thermophiles		2465639	Bacteria	Firmicutes_B	Z-2901	Carboxydothermales	Carboxydothermaceae	Carboxydothermus	*Carboxydothermus pertinax*	870242	*Carboxydothermus pertinax*	https://doi.org/10.1099/ijs.0.031583-0
GCA_000744825.1		Alkaliphiles	2632867	Bacteria	Firmicutes	Bacilli	Lactobacillales	Carnobacteriaceae	Carnobacterium_A	*Carnobacterium_A mobile*	1449342	*Carnobacterium mobile DSM 4848*	https://hal.univ-lorraine.fr/tel-01749410/document
GCA_000195575.1	Psychrophiles	Alkaliphiles	2685399	Bacteria	Firmicutes	Bacilli	Lactobacillales	Carnobacteriaceae	Carnobacterium_A	*Carnobacterium_A sp000195575*	208596	*Carnobacterium sp. 17-4*	https://link.springer.com/article/10.1007/s00792-011-0377-0
GCA_000200715.1	Psychrophiles		2045086	Archaea	Thermoproteota	Nitrososphaeria	Nitrososphaerales	Nitrosopumilaceae	Cenarchaeum	*Cenarchaeum symbiosum*	414004	*Cenarchaeum symbiosum A*	https://doi.org/10.1073/pnas.93.13.6241
GCA_000226295.1	Thermophiles	Alkaliphiles	3695372	Bacteria	Acidobacteriota	Blastocatellia	Chloracidobacteriales	Chloracidobacteriaceae	Chloracidobacterium	*Chloracidobacterium thermophilum*	458033	*Chloracidobacterium thermophilum*	https://doi.org/10.1099/ijs.0.000113
GCA_013340765.1	Thermophiles	Acidophiles	1593902	Archaea	Thermoproteota	Nitrososphaeria	Conexivisphaerales	Conexivisphaeraceae	Conexivisphaera	*Conexivisphaera calida*	1874277	*Conexivisphaera calida*	https://doi.org/10.1099/ijsem.0.004595
GCA_000819445.1		Alkaliphiles	2681312	Bacteria	Actinobacteriota	Actinomycetia	Mycobacteriales	Mycobacteriaceae	Corynebacterium	*Corynebacterium humireducens*	1223515	*Corynebacterium humireducens NBRC 106098 = DSM 45392*	https://doi.org/10.1099/ijs.0.020909-0
GCA_001277255.1	Mesophiles		4499482	Bacteria	Proteobacteria	Gammaproteobacteria	Enterobacterales	Enterobacteriaceae	Cronobacter	*Cronobacter condimenti*	1073999	*Cronobacter condimenti 1330*	https://www.microbiologyresearch.org/content/journal/ijsem/10.1099/ijs.0.032292-0
GCA_001277235.1	Mesophiles		4628405	Bacteria	Proteobacteria	Gammaproteobacteria	Enterobacterales	Enterobacteriaceae	Cronobacter	*Cronobacter dublinensis*	1208656	*Cronobacter dublinensis 1210*	https://www.microbiologyresearch.org/content/journal/ijsem/10.1099/ijs.0.65577-0
GCA_001277215.2	Mesophiles		4473761	Bacteria	Proteobacteria	Gammaproteobacteria	Enterobacterales	Enterobacteriaceae	Cronobacter	*Cronobacter malonaticus*	413503	*Cronobacter malonaticus*	https://www.microbiologyresearch.org/content/journal/ijsem/10.1099/ijs.0.65577-0
GCA_001277195.1	Mesophiles		4364114	Bacteria	Proteobacteria	Gammaproteobacteria	Enterobacterales	Enterobacteriaceae	Cronobacter	*Cronobacter muytjensii*	1159613	*Cronobacter muytjensii ATCC 51329*	https://www.microbiologyresearch.org/content/journal/ijsem/10.1099/ijs.0.65577-0
GCA_000982825.1	Mesophiles		4663565	Bacteria	Proteobacteria	Gammaproteobacteria	Enterobacterales	Enterobacteriaceae	Cronobacter	*Cronobacter sakazakii*	1208592	*Cronobacter sakazakii 680*	https://www.microbiologyresearch.org/content/journal/ijsem/10.1099/ijs.0.65577-0
GCA_000027065.2	Mesophiles		4599092	Bacteria	Proteobacteria	Gammaproteobacteria	Enterobacterales	Enterobacteriaceae	Cronobacter	*Cronobacter turicensis*	1208590	*Cronobacter turicensis 564*	https://www.microbiologyresearch.org/content/journal/ijsem/10.1099/ijs.0.65577-0

GCA_001277175.1	Mesophiles		4436873	Bacteria	Proteobacteria	Gammaproteobacteria	Enterobacterales	Enterobacteriaceae	Cronobacter	*Cronobacter universalis*	1074000	*Cronobacter universalis NCTC 9529*	https://www.microbiologyresearch.org/content/journal/ijsem/10.1099/ijs.0.65577-0
GCA_003185895.1	Psychrophiles		4305516	Bacteria	Actinobacteriota	Actinomycetia	Actinomycetales	Microbacteriaceae	Cryobacterium	*Cryobacterium arcticum*	670052	*Cryobacterium arcticum*	https://doi.org/10.1099/ijs.0.027128-0
GCA_001679725.1	Psychrophiles		4351229	Bacteria	Actinobacteriota	Actinomycetia	Actinomycetales	Microbacteriaceae	Cryobacterium	*Cryobacterium arcticum_A*	670052	*Cryobacterium arcticum*	https://doi.org/10.1099/ijs.0.027128-0
GCA_002954245.1	Psychrophiles		4314199	Bacteria	Actinobacteriota	Actinomycetia	Actinomycetales	Microbacteriaceae	Cryobacterium	*Cryobacterium aureum*	995037	*Cryobacterium aureum*	https://www.microbiologyresearch.org/content/journal/ijsem/10.1099/ijsem.0.002647
GCA_900103805.1	Psychrophiles		4040838	Bacteria	Actinobacteriota	Actinomycetia	Actinomycetales	Microbacteriaceae	Cryobacterium	*Cryobacterium flavum*	1424659	*Cryobacterium flavum*	https://doi.org/10.1099/ijs.0.033738-0
GCA_004402405.1	Psychrophiles		3753316	Bacteria	Actinobacteriota	Actinomycetia	Actinomycetales	Microbacteriaceae	Cryobacterium	*Cryobacterium levicorallinum*	995038	*Cryobacterium levicorallinum*	https://doi.org/10.1099/ijs.0.046896-0
GCA_900110125.1	Psychrophiles		3834069	Bacteria	Actinobacteriota	Actinomycetia	Actinomycetales	Microbacteriaceae	Cryobacterium	*Cryobacterium luteum*	1424661	*Cryobacterium luteum*	https://doi.org/10.1099/ijs.0.033738-0
GCA_004365915.1	Psychrophiles		3677616	Bacteria	Actinobacteriota	Actinomycetia	Actinomycetales	Microbacteriaceae	Cryobacterium	*Cryobacterium psychrophilum*	41988	*Cryobacterium psychrophilum*	https://doi.org/10.1099/00207713-47-2-474
GCA_900101115.1	Psychrophiles		3247111	Bacteria	Actinobacteriota	Actinomycetia	Actinomycetales	Microbacteriaceae	Cryobacterium	*Cryobacterium psychrotolerans*	386301	*Cryobacterium psychrotolerans*	https://doi.org/10.1099/ijs.0.64750-0
GCA_014200405.1	Psychrophiles		4531652	Bacteria	Actinobacteriota	Actinomycetia	Actinomycetales	Microbacteriaceae	Cryobacterium	*Cryobacterium roopkundense*	1001240	*Cryobacterium roopkundense*	https://doi.org/10.1099/ijs.0.011775-0
GCA_002909375.1	Psychrophiles		4048390	Bacteria	Actinobacteriota	Actinomycetia	Actinomycetales	Microbacteriaceae	Cryobacterium	*Cryobacterium zongtaii*	1259217	*Cryobacterium zongtaii*	https://doi.org/10.1016/j.syapm.2018.10.005
GCA_000711975.1		Alkaliphiles	2622239	Bacteria	Firmicutes_B	Desulfotomaculia	Desulfotomaculales	Desulfohalotomaculaceae	Desulfofalx	*Desulfofalx alkaliphila*	1121423	*Desulfofalx alkaliphila DSM 12257*	10.1002/9781118960608.gbm01777
GCA_000686525.1		Alkaliphiles	2939696	Bacteria	Desulfobacterota_I	Desulfovibrionia	Desulfovibrionales	Desulfonatronovibrionaceae	Desulfonatronovibrio	*Desulfonatronovibrio hydrogenovorans*	1121413	*Desulfonatronovibrio hydrogenovorans DSM 9292*	https://doi.org/10.1099/00207713-47-1-144
GCA_000934755.1		Alkaliphiles	4809662	Bacteria	Desulfobacterota_I	Desulfovibrionia	Desulfovibrionales	Desulfonatronovibrionaceae	Desulfonatronovibrio	*Desulfonatronovibrio magnus*	698827	*Desulfonatronovibrio magnus*	https://doi.org/10.1007/s00792-011-0370-7
GCA_000934745.1		Alkaliphiles	4634603	Bacteria	Desulfobacterota_I	Desulfovibrionia	Desulfovibrionales	Desulfonatronaceae	Desulfonatronum	*Desulfonatronum thioautotrophicum*	617001	*Desulfonatronum thioautotrophicum*	https://doi.org/10.1007/s00792-011-0370-7
GCA_900104215.1		Alkaliphiles	3602125	Bacteria	Desulfobacterota_I	Desulfovibrionia	Desulfovibrionales	Desulfonatronaceae	Desulfonatronum	*Desulfonatronum thiosulfatophilum*	617002	*Desulfonatronum thiosulfatophilum*	GCA_900104215.1
GCA_001029285.1		Acidophiles	4637866	Bacteria	Firmicutes_B	Desulfitobacteriia	Desulfitobacteriales	Desulfitobacteriaceae	Desulfosporosinus	*Desulfosporosinus acididurans*	476652	*Desulfosporosinus acididurans*	https://doi.org/10.1007/s00792-014-0701-6
GCA_000255115.3		Acidophiles	4991181	Bacteria	Firmicutes_B	Desulfitobacteriia	Desulfitobacteriales	Desulfitobacteriaceae	Desulfosporosinus	*Desulfosporosinus acidiphilus*	646529	*Desulfosporosinus acidiphilus SJ4*	https://doi.org/10.1007/s00792-014-0701-6
GCA_001707885.1		Acidophiles	4523251	Bacteria	Firmicutes_B	Desulfitobacteriia	Desulfitobacteriales	Desulfitobacteriaceae	Desulfosporosinus	*Desulfosporosinus sp001707885*	1633135	*Desulfosporosinus sp. BG*	https://doi.org/10.1016/j.gdata.2016.12.014
GCA_000429345.1	Thermophiles		3060447	Bacteria	Firmicutes_B	Desulfotomaculia	Desulfotomaculales	Desulfovirgulaceae	Desulfovirgula	*Desulfovirgula thermocuniculi*	1121468	*Desulfovirgula thermocuniculi DSM 16036*	https://doi.org/10.1099/ijs.0.64655-0
GCA_000513855.1	Hyperthermophiles		1307099	Archaea	Thermoproteota	Thermoproteia	Sulfolobales	Desulfurococcaceae	Desulfurococcus	*Desulfurococcus amylolyticus*	490899	*Desulfurococcus amylolyticus 1221n*	https://doi.org/10.1099/ijsem.0.000747
GCA_000186365.1	Hyperthermophiles		1314639	Archaea	Thermoproteota	Thermoproteia	Sulfolobales	Desulfurococcaceae	Desulfurococcus	*Desulfurococcus mucosus*	765177	*Desulfurococcus mucosus 07/1, DSM 2162*	https://doi.org/10.1099/ijsem.0.000747
GCA_000020965.1	Thermophiles		1959987	Bacteria	Dictyoglomota	Dictyoglomia	Dictyoglomales	Dictyoglomaceae	Dictyoglomus	*Dictyoglomus thermophilum*	309799	*Dictyoglomus thermophilum H-6-12*	https://doi.org/10.1099/00207713-35-3-253
GCA_000021645.1	Thermophiles		1855560	Bacteria	Dictyoglomota	Dictyoglomia	Dictyoglomales	Dictyoglomaceae	Dictyoglomus	*Dictyoglomus turgidum*	515635	*Dictyoglomus turgidum DSM 6724*	https://doi.org/10.3389/fmicb.2016.01979
GCA_000376225.1	Thermophiles		3363524	Bacteria	Firmicutes	Bacilli	Tumebacillales	Effusibacillaceae	Effusibacillus	*Effusibacillus pohliae*	1120973	*Effusibacillus pohliae DSM 22757*	https://doi.org/10.1099/ijs.0.055814-0
GCA_000378465.1	Thermophiles		4304237	Bacteria	Proteobacteria	Alphaproteobacteria	Acetobacterales	Acetobacteraceae	Elioraea	*Elioraea tepidiphila*	1121861	*Elioraea tepidiphila DSM 17972*	https://doi.org/10.1099/ijs.0.65294-0
GCA_001544255.1	Mesophiles		2484851	Bacteria	Firmicutes	Bacilli	Lactobacillales	Enterococcaceae	Enterococcus_B	*Enterococcus_B faecium*	1442605	*Enterococcus faecium VSE1036*	https://doi.org/10.1016/j.resmic.2005.11.006
GCA_000759775.1	Mesophiles		4422416	Bacteria	Proteobacteria	Gammaproteobacteria	Enterobacterales	Enterobacteriaceae	Escherichia	*Escherichia albertii*	910238	*Escherichia albertii TW15818*	https://www.sciencedirect.com/science/article/pii/S0963996913000896
GCA_000026225.1	Mesophiles		4643861	Bacteria	Proteobacteria	Gammaproteobacteria	Enterobacterales	Enterobacteriaceae	Escherichia	*Escherichia fergusonii*	564	*Escherichia fergusonii*	https://www.sciencedirect.com/science/article/pii/S0378113514002168?via%3Dihub
GCA_002900365.1	Mesophiles		4896291	Bacteria	Proteobacteria	Gammaproteobacteria	Enterobacterales	Enterobacteriaceae	Escherichia	*Escherichia marmotae*	1499973	*Escherichia marmotae*	https://www.microbiologyresearch.org/content/journal/ijsem/10.1099/ijs.0.000228
GCA_000745905.1		Acidophiles	2928893	Bacteria	Actinobacteriota	Acidimicrobiia	Acidimicrobiales	Acidimicrobiaceae	Ferrimicrobium	*Ferrimicrobium acidiphilum*	1121877	*Ferrimicrobium acidiphilum DSM 19497*	https://doi.org/10.1099/ijs.0.65409-0
GCA_900128965.1		Acidophiles	2489535	Bacteria	Actinobacteriota	Acidimicrobiia	Acidimicrobiales	Acidimicrobiaceae	Ferrithrix	*Ferrithrix thermotolerans*	1121881	*Ferrithrix thermotolerans DSM 19514*	https://doi.org/10.1099/ijs.0.65409-0
GCA_000025505.1	Hyperthermophiles		2196266	Archaea	Halobacteriota	Archaeoglobi	Archaeoglobales	Archaeoglobaceae	Ferroglobus	*Ferroglobus placidus*	589924	*Ferroglobus placidus DSM 10642*	https://doi.org/10.1007/s002030050388
GCA_000152265.2		Acidophiles	1935211	Archaea	Thermoplasmatota	Thermoplasmata	Thermoplasmatales	Thermoplasmataceae	Ferroplasma	*Ferroplasma acidarmanus*	333146	*Ferroplasma acidarmanus fer1*	https://www.microbiologyresearch.org/content/journal/micro/10.1099/mic.0.28076-0
GCA_002078355.1		Acidophiles	1826943	Archaea	Thermoplasmatota	Thermoplasmata	Thermoplasmatales	Thermoplasmataceae	Ferroplasma	*Ferroplasma acidiphilum*	74969	*Ferroplasma acidiphilum*	https://doi.org/10.1099/00207713-50-3-997
GCA_004117075.1	Thermophiles		2266449	Bacteria	Thermotogota	Thermotogae	Thermotogales	Fervidobacteriaceae	Fervidobacterium	*Fervidobacterium changbaicum*	310769	*Fervidobacterium changbaicum*	https://doi.org/10.1099/ijs.0.64758-0
GCA_000235405.3	Thermophiles		2166381	Bacteria	Thermotogota	Thermotogae	Thermotogales	Fervidobacteriaceae	Fervidobacterium	*Fervidobacterium pennivorans*	93466	*Fervidobacterium pennivorans*	https://doi.org/10.3390/microorganisms11010022
GCA_001644665.1	Thermophiles		2061852	Bacteria	Thermotogota	Thermotogae	Thermotogales	Fervidobacteriaceae	Fervidobacterium	*Fervidobacterium pennivorans_A*	93466	*Fervidobacterium pennivorans*	https://doi.org/10.3390/microorganisms11010022
GCA_001719065.1	Thermophiles		2040210	Bacteria	Thermotogota	Thermotogae	Thermotogales	Fervidobacteriaceae	Fervidobacterium_A	*Fervidobacterium_A thailandense*	1008305	*Fervidobacterium thailandense*	https://doi.org/10.1099/ijsem.0.001463
GCA_000419685.1	Psychrophiles		3079036	Bacteria	Bacteroidota	Bacteroidia	Flavobacteriales	Flavobacteriaceae	Flavobacterium	*Flavobacterium antarcticum*	1111730	*Flavobacterium antarcticum DSM 19726*	https://doi.org/10.1099/ijs.0.63423-0
GCA_003259835.1	Psychrophiles	Alkaliphiles	2821499	Bacteria	Bacteroidota	Bacteroidia	Flavobacteriales	Flavobacteriaceae	Flavobacterium	*Flavobacterium aquaticum*	1236486	*Flavobacterium aquaticum*	https://link.springer.com/article/10.1007/s12275-013-2293-8
GCA_015223105.1	Psychrophiles		2830228	Bacteria	Bacteroidota	Bacteroidia	Flavobacteriales	Flavobacteriaceae	Flavobacterium	*Flavobacterium aquaticum_A*	1236486	*Flavobacterium proteolyticum*	https://doi.org/10.1007/s00203-021-02744-2
GCA_003344925.1	Psychrophiles		2970356	Bacteria	Bacteroidota	Bacteroidia	Flavobacteriales	Flavobacteriaceae	Flavobacterium	*Flavobacterium arcticum*	1784713	*Flavobacterium arcticum*	https://doi.org/10.1099/ijsem.0.001804
GCA_900106645.1	Psychrophiles		3856409	Bacteria	Bacteroidota	Bacteroidia	Flavobacteriales	Flavobacteriaceae	Flavobacterium	*Flavobacterium degerlachei*	229203	*Flavobacterium degerlachei*	https://doi.org/10.1099/ijs.0.02857-0
GCA_000425505.1	Psychrophiles		3631041	Bacteria	Bacteroidota	Bacteroidia	Flavobacteriales	Flavobacteriaceae	Flavobacterium	*Flavobacterium frigidarium*	1121890	*Flavobacterium frigidarium DSM 17623*	https://doi.org/10.1099/00207713-51-4-1235
GCA_900111075.1	Psychrophiles		4045707	Bacteria	Bacteroidota	Bacteroidia	Flavobacteriales	Flavobacteriaceae	Flavobacterium	*Flavobacterium frigoris*	229204	*Flavobacterium frigoris*	https://doi.org/10.1099/ijs.0.02857-0
GCA_000252125.2	Psychrophiles		3934101	Bacteria	Bacteroidota	Bacteroidia	Flavobacteriales	Flavobacteriaceae	Flavobacterium	*Flavobacterium frigoris_A*	229204	*Flavobacterium frigoris*	https://doi.org/10.1099/ijs.0.02857-0
GCA_900143245.1	Psychrophiles		3709863	Bacteria	Bacteroidota	Bacteroidia	Flavobacteriales	Flavobacteriaceae	Flavobacterium	*Flavobacterium fryxellicola*	249352	*Flavobacterium fryxellicola*	https://doi.org/10.1099/ijs.0.03056-0
GCA_900107635.1	Psychrophiles		4377959	Bacteria	Bacteroidota	Bacteroidia	Flavobacteriales	Flavobacteriaceae	Flavobacterium	*Flavobacterium gillisiae*	150146	*Flavobacterium gillisiae*	https://doi.org/10.1099/00207713-50-3-1055
GCA_003350545.1	Psychrophiles		3143612	Bacteria	Bacteroidota	Bacteroidia	Flavobacteriales	Flavobacteriaceae	Flavobacterium	*Flavobacterium glaciei*	386300	*Flavobacterium glaciei*	https://doi.org/10.1099/ijs.0.64564-0
GCA_003634755.1	Psychrophiles		3305187	Bacteria	Bacteroidota	Bacteroidia	Flavobacteriales	Flavobacteriaceae	Flavobacterium	*Flavobacterium limicola*	180441	*Flavobacterium limicola*	https://doi.org/10.1099/ijs.0.02369-0
GCA_900129585.1	Psychrophiles		3692790	Bacteria	Bacteroidota	Bacteroidia	Flavobacteriales	Flavobacteriaceae	Flavobacterium	*Flavobacterium micromati*	229205	*Flavobacterium micromati*	https://doi.org/10.1099/ijs.0.02857-0
GCA_900099915.1	Psychrophiles		3808360	Bacteria	Bacteroidota	Bacteroidia	Flavobacteriales	Flavobacteriaceae	Flavobacterium	*Flavobacterium omnivorum*	178355	*Flavobacterium omnivorum*	https://doi.org/10.1099/ijs.0.02310-0
GCA_003312425.1	Psychrophiles		3652041	Bacteria	Bacteroidota	Bacteroidia	Flavobacteriales	Flavobacteriaceae	Flavobacterium	*Flavobacterium psychrolimnae*	249351	*Flavobacterium psychrolimnae*	https://doi.org/10.1099/ijs.0.03056-0
GCA_002217405.1	Psychrophiles		2638051	Bacteria	Bacteroidota	Bacteroidia	Flavobacteriales	Flavobacteriaceae	Flavobacterium	*Flavobacterium psychrophilum*	96345	*Flavobacterium psychrophilum*	https://www.nature.com/articles/nbt1313
GCA_001708385.1	Psychrophiles		4142802	Bacteria	Bacteroidota	Bacteroidia	Flavobacteriales	Flavobacteriaceae	Flavobacterium	*Flavobacterium psychrophilum_A*	96345	*Flavobacterium psychrophilum*	https://www.nature.com/articles/nbt1313
GCA_900129575.1	Psychrophiles		3463995	Bacteria	Bacteroidota	Bacteroidia	Flavobacteriales	Flavobacteriaceae	Flavobacterium	*Flavobacterium segetis*	271157	*Flavobacterium degerlachei*	https://doi.org/10.1099/ijs.0.02857-0
GCA_900110375.1	Psychrophiles	Alkaliphiles	3520953	Bacteria	Bacteroidota	Bacteroidia	Flavobacteriales	Flavobacteriaceae	Flavobacterium	*Flavobacterium sinopsychrotolerans*	604089	*Flavobacterium sinopsychrotolerans*	https://doi.org/10.1099/ijs.0.014126-0
GCA_000425485.1	Psychrophiles		3774544	Bacteria	Bacteroidota	Bacteroidia	Flavobacteriales	Flavobacteriaceae	Flavobacterium	*Flavobacterium tegetincola*	1121901	*Flavobacterium tegetincola DSM 22377*	https://www.microbiologyresearch.org/content/journal/ijsem/10.1099/00207713-50-3-1055
GCA_900108015.1	Psychrophiles		3439151	Bacteria	Bacteroidota	Bacteroidia	Flavobacteriales	Flavobacteriaceae	Flavobacterium	*Flavobacterium urumqiense*	935224	*Flavobacterium urumqiense*	https://doi.org/10.1099/ijs.0.030049-0
GCA_003688495.1	Psychrophiles		3397857	Bacteria	Bacteroidota	Bacteroidia	Flavobacteriales	Flavobacteriaceae	Flavobacterium	*Flavobacterium weaverense*	271156	*Flavobacterium weaverense*	https://doi.org/10.1099/ijs.0.64164-0

GCA_900142695.1	Psychrophiles		3755582	Bacteria	Bacteroidota	Bacteroidia	Flavobacteriales	Flavobacteriaceae	Flavobacterium	*Flavobacterium xanthum*	69322	*Flavobacterium xanthum*	https://www.microbiologyresearch.org/content/journal/ijsem/10.1099/00207713-50-3-1055
GCA_900142885.1	Psychrophiles		3902806	Bacteria	Bacteroidota	Bacteroidia	Flavobacteriales	Flavobacteriaceae	Flavobacterium	*Flavobacterium xinjiangense*	178356	*Flavobacterium xinjiangense*	https://doi.org/10.1099/ijs.0.02310-0
GCA_900112975.1	Psychrophiles		3471047	Bacteria	Bacteroidota	Bacteroidia	Flavobacteriales	Flavobacteriaceae	Flavobacterium	*Flavobacterium xueshanense*	935223	*Flavobacterium xueshanense*	https://doi.org/10.1099/ijs.0.030049-0
GCA_000820505.1		Alkaliphiles	3839692	Bacteria	Desulfobacterota	Desulfuromonadia	Desulfuromonadales	Geoalkalibacteraceae	Geoalkalibacter	*Geoalkalibacter ferrihydriticus*	1121915	*Geoalkalibacter ferrihydriticus DSM 17813*	https://pubmed.ncbi.nlm.nih.gov/17205802/
GCA_000789255.1	Hyperthermophiles		1860815	Archaea	Halobacteriota	Archaeoglobi	Archaeoglobales	Archaeoglobaceae	Geoglobus	*Geoglobus acetivorans*	565033	*Geoglobus acetivorans*	https://doi.org/10.1128/aem.02705-14
GCA_015163485.1	Hyperthermophiles		1901114	Archaea	Halobacteriota	Archaeoglobi	Archaeoglobales	Archaeoglobaceae	Geoglobus	*Geoglobus acetivorans_A*	565033	*Geoglobus acetivorans*	https://doi.org/10.1128/aem.02705-14
GCA_001006045.1	Hyperthermophiles		1770093	Archaea	Halobacteriota	Archaeoglobi	Archaeoglobales	Archaeoglobaceae	Geoglobus	*Geoglobus ahangari*	113653	*Geoglobus ahangari*	https://doi.org/10.1099/00207713-52-3-719
GCA_002952775.1		Alkaliphiles	3313120	Archaea	Halobacteriota	Halobacteria	Halobacteriales	Haloferacaceae	Halalkaliarchaeum	*Halalkaliarchaeum desulfuricum*	2055893	*Halalkaliarchaeum desulfuricum*	https://doi.org/10.1099/ijsem.0.003506
GCA_000423105.1		Alkaliphiles	2707549	Bacteria	Firmicutes	Bacilli	Bacillales_D	Alkalibacillaceae	Halalkalibacillus	*Halalkalibacillus halophilus*	1121936	*Halalkalibacillus halophilus DSM 18494*	https://doi.org/10.1099/ijs.0.64830-0
GCA_000196895.1	Thermophiles	Alkaliphiles	3698650	Archaea	Halobacteriota	Halobacteria	Halobacteriales	Halalkalicoccaceae	Halalkalicoccus	*Halalkalicoccus jeotgali*	795797	*Halalkalicoccus jeotgali*	https://pubmed.ncbi.nlm.nih.gov/17911300/
GCA_004799665.1	Mesophiles		3452056	Archaea	Halobacteriota	Halobacteria	Halobacteriales	Haloarculaceae	Halapricum	*Halapricum salinum*	1457250	*Halapricum salinum strain CBA1105*	https://pubmed.ncbi.nlm.nih.gov/24677144/
GCA_014647455.2		Acidophiles	3024665	Archaea	Halobacteriota	Halobacteria	Halobacteriales	Halobacteriaceae	Halarchaeum	*Halarchaeum grantii*	1193105	*Halarchaeum grantii*	https://doi.org/10.1099/ijsem.0.000501
GCA_014647155.1		Acidophiles	3141185	Archaea	Halobacteriota	Halobacteria	Halobacteriales	Halobacteriaceae	Halarchaeum	*Halarchaeum nitratireducens*	489913	*Halarchaeum nitratireducens*	https://doi.org/10.1099/ijs.0.054668-0
GCA_014647115.1	Mesophiles	Acidophiles	2813544	Archaea	Halobacteriota	Halobacteria	Halobacteriales	Halobacteriaceae	Halarchaeum	*Halarchaeum rubridurum*	489911	*Halarchaeum rubridurum*	https://doi.org/10.1099/ijs.0.049262-0
GCA_000336615.1	Thermophiles	Alkaliphiles	4225424	Archaea	Halobacteriota	Halobacteria	Halobacteriales	Haloarculaceae	Haloarcula	*Haloarcula amylolytica*	1227452	*Haloarcula amylolytica JCM 13557*	https://www.microbiologyresearch.org/content/journal/ijsem/10.1099/ijs.0.64647-0
GCA_000336895.1	Mesophiles		4147107	Archaea	Halobacteriota	Halobacteria	Halobacteriales	Haloarculaceae	Haloarcula	*Haloarcula argentinensis*	1230451	*Haloarcula argentinensis*	https://www.microbiologyresearch.org/content/journal/ijsem/10.1099/00207713-47-1-73
GCA_010119195.1	Mesophiles		3788104	Archaea	Halobacteriota	Halobacteria	Halobacteriales	Haloarculaceae	Haloarcula	*Haloarcula salina*	1429914	*Halomicroarcula salina*	https://pubmed.ncbi.nlm.nih.gov/25721722/
GCA_000337775.1	Thermophiles		3923205	Archaea	Halobacteriota	Halobacteria	Halobacteriales	Haloarculaceae	Haloarcula	*Haloarcula vallismortis*	662477	*Haloarcula vallismortis*	https://www.ncbi.nlm.nih.gov/pmc/articles/PMC545725/
GCA_008124605.1	Mesophiles		2364912	Archaea	Halobacteriota	Halobacteria	Halobacteriales	Halobacteriaceae	Halobacterium	*Halobacterium salinarum*	2597657	*Halobacterium salinarum*	https://doi.org/10.1089%2Fomi.2012.0117
GCA_000336935.1	Mesophiles	Alkaliphiles	4199784	Archaea	Halobacteriota	Halobacteria	Halobacteriales	Halococcaceae	Halococcus	*Halococcus salifodinae*	1227456	*Halococcus salifodinae*	https://doi.org/10.1016/j.ijbiomac.2021.02.081
GCA_000337795.1	Thermophiles		3825973	Archaea	Halobacteriota	Halobacteria	Halobacteriales	Haloferacaceae	Haloferax	*Haloferax denitrificans*	662478	*Haloferax denitrificans*	https://www.microbiologyresearch.org/content/journal/ijsem/10.1099/00207713-39-3-359?crawler=true
GCA_000336755.1	Thermophiles	Acidophiles	3952136	Archaea	Halobacteriota	Halobacteria	Halobacteriales	Haloferacaceae	Haloferax	*Haloferax elongans*	1230453	*Haloferax elongans*	https://www.microbiologyresearch.org/content/journal/ijsem/10.1099/ijs.0.65360-0
GCA_000306765.2	Thermophiles		3904707	Archaea	Halobacteriota	Halobacteria	Halobacteriales	Haloferacaceae	Haloferax	*Haloferax mediterranei*	523841	*Haloferax mediterranei*	https://www.ncbi.nlm.nih.gov/pmc/articles/PMC7915512/
GCA_000337815.1	Thermophiles		3368982	Archaea	Halobacteriota	Halobacteria	Halobacteriales	Haloferacaceae	Haloferax	*Haloferax mucosum*	662479	*Haloferax mucosum*	https://www.microbiologyresearch.org/content/journal/ijsem/10.1099/ijs.0.65360-0
GCA_000025685.1	Mesophiles		4012900	Archaea	Halobacteriota	Halobacteria	Halobacteriales	Haloferacaceae	Haloferax	*Haloferax volcanii*	309800	*Haloferax volcanii*	https://www.ncbi.nlm.nih.gov/pmc/articles/PMC545725/
GCA_000172995.2	Thermophiles		3944467	Archaea	Halobacteriota	Halobacteria	Halobacteriales	Haloferacaceae	Halogeometricum	*Halogeometricum borinquense*	469382	*Halogeometricum borinquense*	https://www.ncbi.nlm.nih.gov/pmc/articles/PMC545725/
GCA_900112175.1	Thermophiles		4187125	Archaea	Halobacteriota	Halobacteria	Halobacteriales	Haloferacaceae	Halogeometricum	*Halogeometricum rufum*	553469	*Halogeometricum rufum*	https://www.microbiologyresearch.org/content/journal/ijsem/10.1099/ijs.0.019463-0
GCA_900110465.1	Mesophiles		5185690	Archaea	Halobacteriota	Halobacteria	Halobacteriales	Haloferacaceae	Halogranum	*Halogranum amylolyticum*	660520	*Halogranum amylolyticum*	https://doi.org/10.1099/ijs.0.024976-0
GCA_900103715.1	Mesophiles		3770187	Archaea	Halobacteriota	Halobacteria	Halobacteriales	Haloferacaceae	Halogranum	*Halogranum gelatinilyticum*	660521	*Halogranum gelatinilyticum*	https://doi.org/10.1099/ijs.0.024976-0
GCA_009791395.1	Mesophiles		4069707	Archaea	Halobacteriota	Halobacteria	Halobacteriales	Haloarculaceae	Halomarina	*Halomarina oriensis*	671145	*Halomarina oriensis*	https://doi.org/10.1099/ijs.0.020677-0
GCA_003862495.1	Mesophiles		3654689	Archaea	Halobacteriota	Halobacteria	Halobacteriales	Haloarculaceae	Halomarina	*Halomarina oriensis_A*	671145	*Halocatena pleomorpha*	https://doi.org/10.1099/ijsem.0.004222
GCA_010119205.1	Mesophiles		3906684	Archaea	Halobacteriota	Halobacteria	Halobacteriales	Haloarculaceae	Halomicroarcula	*Halomicroarcula limicola*	1429915	*Halomicroarcula limicola*	https://pubmed.ncbi.nlm.nih.gov/24554639/
GCA_000379085.1	Mesophiles		3607771	Archaea	Halobacteriota	Halobacteria	Halobacteriales	Haloarculaceae	Halomicrobium	*Halomicrobium katesii*	1069082	*Halomicrobium katesii DSM 19301*	https://doi.org/10.1099/ijs.0.65662-0
GCA_000023965.1	Mesophiles		3332349	Archaea	Halobacteriota	Halobacteria	Halobacteriales	Haloarculaceae	Halomicrobium	*Halomicrobium mukohataei*	485914	*Halomicrobium mukohataei DSM 12286*	https://www.microbiologyresearch.org/content/journal/ijsem/10.1099/00207713-52-5-1831
GCA_900114435.1	Mesophiles		4250330	Archaea	Halobacteriota	Halobacteria	Halobacteriales	Haloarculaceae	Halomicrobium	*Halomicrobium zhouii*	767519	*Halomicrobium zhouii*	https://doi.org/10.1099/ijs.0.031989-0
GCA_002966495.1		Alkaliphiles	3650492	Bacteria	Proteobacteria	Gammaproteobacteria	Pseudomonadales	Halomonadaceae	Halomonas	*Halomonas sp002966495*	1118153	*Halomonas sp. GFAJ-1*	https://www.ncbi.nlm.nih.gov/pmc/articles/PMC6598117/
GCA_900100875.1	Mesophiles		3871751	Archaea	Halobacteriota	Halobacteria	Halobacteriales	Haloferacaceae	Halopelagius	*Halopelagius longus*	1236180	*Halopelagius longus*	https://doi.org/10.1099/ijs.0.051375-0
GCA_000455345.1	Thermophiles	Alkaliphiles	3906364	Archaea	Halobacteriota	Halobacteria	Halobacteriales	Natrialbaceae	Halopiger_A	*Halopiger_A goleimassiliensis*	1293048	*Halopiger goleimassiliensis*	http://standardsingenomics.org/content/9/3/956/
GCA_000470655.1	Mesophiles		3146160	Archaea	Halobacteriota	Halobacteria	Halobacteriales	Haloarculaceae	Halorhabdus	*Halorhabdus tiamatea*	1033806	*Halorhabdus tiamatea SARL4B*	https://doi.org/10.1099/ijs.0.65316-0
GCA_000023945.1	Mesophiles		3116795	Archaea	Halobacteriota	Halobacteria	Halobacteriales	Haloarculaceae	Halorhabdus	*Halorhabdus utahensis*	519442	*Halorhabdus utahensis DSM 12940*	https://www.microbiologyresearch.org/content/journal/ijsem/10.1099/00207713-50-1-183
GCA_001542905.1	Mesophiles		3325770	Archaea	Halobacteriota	Halobacteria	Halobacteriales	Haloferacaceae	Halorubrum	*Halorubrum aethiopicum*	1758255	*Halorubrum aethiopicum*	https://doi.org/10.1099/ijsem.0.002525
GCA_000336995.1	Thermophiles		3108525	Archaea	Halobacteriota	Halobacteria	Halobacteriales	Haloferacaceae	Halorubrum	*Halorubrum aidingense*	1230454	*Halorubrum aidingense*	https://www.microbiologyresearch.org/content/journal/ijsem/10.1099/ijs.0.64305-0
GCA_900182635.1	Mesophiles		3176020	Archaea	Halobacteriota	Halobacteria	Halobacteriales	Haloferacaceae	Halorubrum	*Halorubrum cibi*	413815	*Halorubrum cibi*	https://link.springer.com/article/10.1007/s12275-009-0016-y
GCA_000337035.1	Mesophiles		3645313	Archaea	Halobacteriota	Halobacteria	Halobacteriales	Haloferacaceae	Halorubrum	*Halorubrum coriense*	1227466	*Halorubrum coriense*	https://bacdive.dsmz.de/strain/5944
GCA_000022205.1	Psychrophiles		3692576	Archaea	Halobacteriota	Halobacteria	Halobacteriales	Haloferacaceae	Halorubrum	*Halorubrum lacusprofundi*	416348	*Halorubrum lacusprofundi ATCC 49239*	https://doi.org/10.1111/1462-2920.13705
GCA_000337375.1	Thermophiles		3425042	Archaea	Halobacteriota	Halobacteria	Halobacteriales	Haloferacaceae	Halorubrum	*Halorubrum lipolyticum*	1227482	*Halorubrum lipolyticum*	https://www.microbiologyresearch.org/content/journal/ijsem/10.1099/ijs.0.64305-0
GCA_000337915.1	Thermophiles		3423703	Archaea	Halobacteriota	Halobacteria	Halobacteriales	Haloferacaceae	Halorubrum	*Halorubrum saccharovorum*	1227484	*Halorubrum saccharovorum*	https://www.ncbi.nlm.nih.gov/pmc/articles/PMC545725/
GCA_900111935.1	Mesophiles		3030553	Archaea	Halobacteriota	Halobacteria	Halobacteriales	Haloferacaceae	Halorubrum	*Halorubrum sodomense*	35743	*Halorubrum sodomense*	https://www.microbiologyresearch.org/content/journal/ijsem/10.1099/00207713-33-2-381
GCA_018228765.1	Mesophiles		3021820	Archaea	Halobacteriota	Halobacteria	Halobacteriales	Haloferacaceae	Halorubrum	*Halorubrum sp000296615*	35743	*Halorubrum ruber*	https://doi.org/10.1007/s12275-022-2173-1
GCA_900188065.1	Mesophiles	Alkaliphiles	3477860	Archaea	Halobacteriota	Halobacteria	Halobacteriales	Haloferacaceae	Halorubrum	*Halorubrum vacuolatum*	63740	*Halorubrum vacuolatum*	https://bacdive.dsmz.de/strain/5943
GCA_004765815.2	Mesophiles		4753237	Archaea	Halobacteriota	Halobacteria	Halobacteriales	Haladaptataceae	Halorussus	*Halorussus salinus*	1364935	*Halorussus salinus*	https://doi.org/10.1007/s00203-016-1253-1
GCA_900116205.1	Mesophiles		4108147	Archaea	Halobacteriota	Halobacteria	Halobacteriales	Natrialbaceae	Halostagnicola	*Halostagnicola kamekurae*	619731	*Halostagnicola kamekurae*	https://doi.org/10.1099/ijs.0.014449-0
GCA_005954745.1	Mesophiles		3942449	Archaea	Halobacteriota	Halobacteria	Halobacteriales	QS-9-68-17	Halostella	*Halostella pelagica*	2583824	*Halostella pelagica*	https://doi.org/10.1099/ijsem.0.004003
GCA_000025325.1	Thermophiles		5440782	Archaea	Halobacteriota	Halobacteria	Halobacteriales	Natrialbaceae	Haloterrigena	*Haloterrigena turkmenica*	543526	*Haloterrigena turkmenica*	https://www.ncbi.nlm.nih.gov/pmc/articles/PMC545725/
GCA_000378345.1		Alkaliphiles	3351270	Bacteria	Proteobacteria	Alphaproteobacteria	Caulobacterales	Hyphomonadaceae	Hirschia	*Hirschia maritima*	551275	*Hirschia maritima DSM 19733*	https://www.sciencedirect.com/science/article/pii/S221359601530101X?via%3Dihub
GCA_003574215.1	Thermophiles		2288780	Bacteria	Proteobacteria	Gammaproteobacteria	Burkholderiales	Rhodocyclaceae	Hydrogenophilus	*Hydrogenophilus thermoluteolus*	297	*Hydrogenophilus thermoluteolus*	https://doi.org/10.1099/00207713-49-2-783
GCA_000015145.1	Hyperthermophiles		1667163	Archaea	Thermoproteota	Thermoproteia	Sulfolobales	Pyrodictiaceae	Hyperthermus	*Hyperthermus butylicus*	415426	*Hyperthermus butylicus DSM 5456*	https://doi.org/10.1128/jb.172.7.3959-3965.1990
GCA_001481685.1	Hyperthermophiles		1394664	Archaea	Thermoproteota	Thermoproteia	Sulfolobales	Ignicoccaceae	Ignicoccus	*Ignicoccus islandicus*	54259	*Ignicoccus islandicus DSM 13165*	https://doi.org/10.1099/00207713-50-6-2093
GCA_000017945.1	Hyperthermophiles		1297538	Archaea	Thermoproteota	Thermoproteia	Sulfolobales	Ignicoccaceae	Ignicoccus_A	*Ignicoccus_A hospitalis*	453591	*Ignicoccus hospitalis KIN4/I*	https://www.microbiologyresearch.org/content/journal/ijsem/10.1099/ijs.0.64721-0
GCA_000145985.1	Hyperthermophiles		1875953	Archaea	Thermoproteota	Thermoproteia	Sulfolobales	Ignisphaeraceae	Ignisphaera	*Ignisphaera aggregans*	334771	*Ignisphaera aggregans AQ1.S1, DSM 17230*	https://doi.org/10.1099/ijs.0.63899-0
GCA_000525795.1	Mesophiles		2601148	Bacteria	Firmicutes	Bacilli	Lactobacillales	Listeriaceae	Listeria	*Listeria aquatica*	1265818	*Listeria aquatica FSL S10-1188*	https://www.microbiologyresearch.org/content/journal/ijsem/10.1099/ijs.0.052720-0

GCA_000344175.1	Mesophiles		2841134	Bacteria	Firmicutes	Bacilli	Lactobacillales	Listeriaceae	Listeria	*Listeria fleischmannii*	1430899	*Listeria fleischmannii 1991*	https://bacdive.dsmz.de/strain/23081
GCA_000525875.1	Mesophiles		2794388	Bacteria	Firmicutes	Bacilli	Lactobacillales	Listeriaceae	Listeria	*Listeria floridensis*	1265817	*Listeria floridensis FSL S10-1187*	https://www.microbiologyresearch.org/content/journal/ijsem/10.1099/ijs.0.052720-0
GCA_000148995.1	Mesophiles		2598321	Bacteria	Firmicutes	Bacilli	Lactobacillales	Listeriaceae	Listeria	*Listeria grayi*	1641	*Listeria grayi*	https://bacdive.dsmz.de/strain/6870
GCA_015276835.1	Mesophiles		2878821	Bacteria	Firmicutes	Bacilli	Lactobacillales	Listeriaceae	Listeria	*Listeria innocua*	1642	*Listeria innocua*	https://bacdive.dsmz.de/strain/136614
GCA_900187025.1	Mesophiles		2919550	Bacteria	Firmicutes	Bacilli	Lactobacillales	Listeriaceae	Listeria	*Listeria ivanovii*	881621	*Listeria ivanovii subsp. ivanovii PAM 55*	https://bacdive.dsmz.de/strain/6872
GCA_013282665.1	Mesophiles		2788056	Bacteria	Firmicutes	Bacilli	Lactobacillales	Listeriaceae	Listeria	*Listeria monocytogenes_C*	552536	*Listeria monocytogenes HCC23*	https://bacdive.dsmz.de/strain/6875
GCA_000027145.1	Mesophiles		2797636	Bacteria	Firmicutes	Bacilli	Lactobacillales	Listeriaceae	Listeria	*Listeria seeligeri*	1640	*Listeria seeligeri*	https://www.microbiologyresearch.org/content/journal/ijsem/10.1099/00207713-33-4-866
GCA_000060285.1	Mesophiles		2814130	Bacteria	Firmicutes	Bacilli	Lactobacillales	Listeriaceae	Listeria	*Listeria welshimeri*	1643	*Listeria welshimeri*	https://www.microbiologyresearch.org/content/journal/ijsem/10.1099/00207713-33-4-866
GCA_000766865.1	Mesophiles		3436956	Bacteria	Firmicutes	Bacilli	Lactobacillales	Listeriaceae	Listeria_A	*Listeria_A booriae*	1552123	*Listeria booriae*	https://www.microbiologyresearch.org/content/journal/ijsem/10.1099/ijs.0.070839-0
GCA_000766145.1	Mesophiles		3515436	Bacteria	Firmicutes	Bacilli	Lactobacillales	Listeriaceae	Listeria_A	*Listeria_A newyorkensis*	1497681	*Listeria newyorkensis*	https://www.microbiologyresearch.org/content/journal/ijsem/10.1099/ijs.0.070839-0
GCA_000525995.1	Mesophiles		3291042	Bacteria	Firmicutes	Bacilli	Lactobacillales	Listeriaceae	Listeria_A	*Listeria_A riparia*	1265816	*Listeria riparia FSL S10-1204*	https://www.microbiologyresearch.org/content/journal/ijsem/10.1099/ijs.0.052720-0
GCA_000525975.1	Mesophiles		3216749	Bacteria	Firmicutes	Bacilli	Lactobacillales	Listeriaceae	Listeria_A	*Listeria_A rocourtiae*	647910	*Listeria rocourtiae*	https://www.microbiologyresearch.org/content/journal/ijsem/10.1099/ijs.0.017376-0
GCA_003534205.1	Mesophiles		3406292	Bacteria	Firmicutes	Bacilli	Lactobacillales	Listeriaceae	Listeria_A	*Listeria_A weihenstephanensis*	1006155	*Listeria weihenstephanensis*	https://www.microbiologyresearch.org/content/journal/ijsem/10.1099/ijs.0.036830-0
GCA_009765685.1		Acidophiles	6380195	Bacteria	Proteobacteria	Alphaproteobacteria	Acetobacterales	Acetobacteraceae	LMUY01	*LMUY01 sp009765685*	1641851	*Acidisphaera sp. L21*	https://doi.org/10.1099/00207713-50-4-1539
GCA_003970575.1	Mesophiles		2605275	Bacteria	Firmicutes	Bacilli	Staphylococcales	Staphylococcaceae	Mammaliicoccus	*Mammaliicoccus fleurettii*	150056	*Mammaliicoccus fleurettii*	https://bacdive.dsmz.de/strain/14648
GCA_002902755.1	Mesophiles		2546437	Bacteria	Firmicutes	Bacilli	Staphylococcales	Staphylococcaceae	Mammaliicoccus	*Mammaliicoccus lentus*	42858	*Mammaliicoccus lentus*	https://bacdive.dsmz.de/strain/14560
GCA_002901825.1	Mesophiles		2768322	Bacteria	Firmicutes	Bacilli	Staphylococcales	Staphylococcaceae	Mammaliicoccus	*Mammaliicoccus sciuri*	1296	*Mammaliicoccus sciuri*	https://bacdive.dsmz.de/strain/14631
GCA_002902265.1	Mesophiles		2595808	Bacteria	Firmicutes	Bacilli	Staphylococcales	Staphylococcaceae	Mammaliicoccus	*Mammaliicoccus vitulinus*	71237	*Mammaliicoccus vitulinus*	https://bacdive.dsmz.de/strain/138135
GCA_900142385.1	Psychrophiles		3740786	Bacteria	Proteobacteria	Gammaproteobacteria	Pseudomonadales	Oleiphilaceae	Marinobacter	*Marinobacter antarcticus*	564117	*Marinobacter antarcticus*	https://doi.org/10.1099/ijs.0.035774-0
GCA_011049865.1	Psychrophiles		3929789	Bacteria	Proteobacteria	Gammaproteobacteria	Pseudomonadales	Oleiphilaceae	Marinobacter	*Marinobacter antarcticus_A*	564117	*Marinobacter antarcticus*	https://doi.org/10.1099/ijs.0.035774-0
GCA_007671675.1	Psychrophiles		4357025	Bacteria	Proteobacteria	Gammaproteobacteria	Pseudomonadales	Oleiphilaceae	Marinobacter	*Marinobacter maritimus*	277961	*Marinobacter maritimus*	https://doi.org/10.1099/ijs.0.63478-0
GCA_900106945.1	Mesophiles		3971609	Bacteria	Proteobacteria	Gammaproteobacteria	Pseudomonadales	Oleiphilaceae	Marinobacter	*Marinobacter mobilis*	488533	*Marinobacter mobilis*	https://www.microbiologyresearch.org/content/journal/ijsem/10.1099/ijs.0.2008/000786-0
GCA_007671655.1	Mesophiles		3336972	Bacteria	Proteobacteria	Gammaproteobacteria	Pseudomonadales	Oleiphilaceae	Marinobacter	*Marinobacter piscensis*	1562308	*Marinobacter piscensis*	https://doi.org/10.1007/s00284-014-0754-x
GCA_001043175.1	Psychrophiles		3998597	Bacteria	Proteobacteria	Gammaproteobacteria	Pseudomonadales	Oleiphilaceae	Marinobacter	*Marinobacter psychrophilus*	330734	*Marinobacter psychrophilus*	https://doi.org/10.1099/ijs.0.65690-0
GCA_000831005.1	Mesophiles		4616532	Bacteria	Proteobacteria	Gammaproteobacteria	Pseudomonadales	Oleiphilaceae	Marinobacter	*Marinobacter salarius*	1420917	*Marinobacter salarius*	https://doi.org/10.1371/journal.pone.0106514
GCA_002806945.1		Alkaliphiles	3820421	Bacteria	Proteobacteria	Gammaproteobacteria	Pseudomonadales	Oleiphilaceae	Marinobacter	*Marinobacter salexigens*	1925763	*Marinobacter salexigens*	https://doi.org/10.1099/ijsem.0.002337
GCA_018860765.1		Alkaliphiles	4133335	Bacteria	Proteobacteria	Gammaproteobacteria	Pseudomonadales	Oleiphilaceae	Marinobacter	*Marinobacter salexigens_A*	1925763	*Marinobacter salexigens*	https://doi.org/10.1099/ijsem.0.002337
GCA_009617755.1	Mesophiles		4150758	Bacteria	Proteobacteria	Gammaproteobacteria	Pseudomonadales	Oleiphilaceae	Marinobacter	*Marinobacter salsuginis*	418719	*Marinobacter salsuginis*	https://doi.org/10.1099/ijs.0.64862-0
GCA_000347775.1	Mesophiles		4033468	Bacteria	Proteobacteria	Gammaproteobacteria	Pseudomonadales	Oleiphilaceae	Marinobacter	*Marinobacter santoriniensis*	1288826	*Marinobacter santoriniensis NKSG1*	https://doi.org/10.1099/ijs.0.003145-0
GCA_900111555.1	Mesophiles		4218891	Bacteria	Proteobacteria	Gammaproteobacteria	Pseudomonadales	Oleiphilaceae	Marinobacter	*Marinobacter segnicrescens*	430453	*Marinobacter segnicrescens*	https://doi.org/10.1099/ijs.0.65030-0
GCA_000364845.1	Mesophiles		5358909	Bacteria	Proteobacteria	Gammaproteobacteria	Pseudomonadales	Oleiphilaceae	Marinobacter_A	*Marinobacter_A nanhaiticus*	626887	*Marinobacter nanhaiticus D15-8W*	https://pubmed.ncbi.nlm.nih.gov/23117603/
GCA_000620065.1	Thermophiles		3034817	Bacteria	Deinococcota	Deinococci	Deinococcales	Thermaceae	Meiothermus	*Meiothermus cerbereus*	1122221	*Meiothermus cerbereus DSM 11376*	https://doi.org/10.1099/00207713-47-4-1225
GCA_003574035.1	Thermophiles		3684677	Bacteria	Deinococcota	Deinococci	Deinococcales	Thermaceae	Meiothermus	*Meiothermus hypogaeus*	884155	*Meiothermus hypogaeus*	https://doi.org/10.1099/ijs.0.028654-0
GCA_003574085.1	Thermophiles		2874609	Bacteria	Deinococcota	Deinococci	Deinococcales	Thermaceae	Meiothermus	*Meiothermus luteus*	2026184	*Meiothermus luteus*	https://doi.org/10.1099/ijsem.0.002040
GCA_000423425.1	Thermophiles		2747076	Bacteria	Deinococcota	Deinococci	Deinococcales	Thermaceae	Meiothermus	*Meiothermus rufus*	604331	*Meiothermus rufus DSM 22234*	https://doi.org/10.1016/j.syapm.2009.05.002
GCA_000482765.1	Thermophiles		3020616	Bacteria	Deinococcota	Deinococci	Deinococcales	Thermaceae	Meiothermus	*Meiothermus taiwanensis*	172827	*Meiothermus taiwanensis*	https://doi.org/10.1099/00207713-52-5-1647
GCA_000092125.1	Thermophiles		3721669	Bacteria	Deinococcota	Deinococci	Deinococcales	Thermaceae	Meiothermus_B	*Meiothermus_B silvanus*	526227	*Meiothermus silvanus DSM 9946*	https://bacdive.dsmz.de/strain/16703
GCA_000204925.1	Thermophiles	Acidophiles	1840348	Archaea	Thermoproteota	Thermoproteia	Sulfolobales	Sulfolobaceae	Metallosphaera	*Metallosphaera cuprina*	1006006	*Metallosphaera cuprina Ar-4*	https://doi.org/10.1099/ijs.0.026591-0
GCA_003201675.2	Thermophiles	Acidophiles	2544115	Archaea	Thermoproteota	Thermoproteia	Sulfolobales	Sulfolobaceae	Metallosphaera	*Metallosphaera hakonensis*	1293036	*Metallosphaera hakonensis JCM 8857 = DSM 7519*	https://doi.org/10.1099%2F00207713-46-2-377
GCA_000016605.1	Thermophiles		2191517	Archaea	Thermoproteota	Thermoproteia	Sulfolobales	Sulfolobaceae	Metallosphaera	*Metallosphaera sedula*	43687	*Metallosphaera sedula*	https://bacdive.dsmz.de/strain/16645
GCA_013343295.1	Thermophiles	Acidophiles	2176897	Archaea	Thermoproteota	Thermoproteia	Sulfolobales	Sulfolobaceae	Metallosphaera	*Metallosphaera tengchongensis*	1532350	*Metallosphaera tengchongensis*	https://doi.org/10.1099/ijs.0.070870-0
GCA_000243315.1	Thermophiles	Acidophiles	2817452	Archaea	Thermoproteota	Thermoproteia	Sulfolobales	Sulfolobaceae	Metallosphaera	*Metallosphaera yellowstonensis*	671065	*Metallosphaera yellowstonensis MK1*	https://doi.org/10.1128/aem.03416-13
GCA_017873625.1	Mesophiles		2468550	Archaea	Methanobacteriota	Methanobacteria	Methanobacteriales	Methanobacteriaceae	Methanobacterium	*Methanobacterium petrolearium*	710190	*Methanobacterium petrolearium*	https://doi.org/10.1099/ijs.0.022723-0
GCA_002813695.1	Mesophiles		2515817	Archaea	Methanobacteriota	Methanobacteria	Methanobacteriales	Methanobacteriaceae	Methanobacterium	*Methanobacterium subterraneum*	59277	*Methanobacterium subterraneum*	https://doi.org/10.1099/00207713-48-2-357
GCA_000214725.1	Mesophiles		2546541	Archaea	Methanobacteriota	Methanobacteria	Methanobacteriales	Methanobacteriaceae	Methanobacterium_C	*Methanobacterium_C paludis*	868131	*Methanobacterium paludis*	https://doi.org/10.1099/ijs.0.059964-0
GCA_001639275.1	Mesophiles		2140433	Archaea	Methanobacteriota	Methanobacteria	Methanobacteriales	Methanobacteriaceae	Methanobrevibacter_A	*Methanobrevibacter_A oralis*	1415626	*Methanobrevibacter oralis*	https://bacdive.dsmz.de/strain/6969
GCA_002072215.1	Mesophiles		2445031	Archaea	Methanobacteriota	Methanobacteria	Methanobacteriales	Methanobacteriaceae	Methanobrevibacter_C	*Methanobrevibacter_C arboriphilus*	39441	*Methanobrevibacter arboriphilus*	https://bacdive.dsmz.de/strain/6957
GCA_000513315.1	Mesophiles		2221920	Archaea	Methanobacteriota	Methanobacteria	Methanobacteriales	Methanobacteriaceae	Methanobrevibacter_C	*Methanobrevibacter_C arboriphilus_A*	39441	*Methanobrevibacter arboriphilus*	https://bacdive.dsmz.de/strain/6957
GCA_000739065.1	Hyperthermophiles		1607556	Archaea	Methanobacteriota_A	Methanococci	Methanococcales	Methanocaldococcaceae	Methanocaldococcus	*Methanocaldococcus bathoardescens*		*Methanocaldococcus bathoardescens*	https://doi.org/10.1099/ijs.0.000097
GCA_000023985.1	Thermophiles		1507251	Archaea	Methanobacteriota_A	Methanococci	Methanococcales	Methanocaldococcaceae	Methanocaldococcus	*Methanocaldococcus fervens*	573064	*Methanocaldococcus fervens AG86*	https://pubmed.ncbi.nlm.nih.gov/10319479/
GCA_000091665.1	Thermophiles		1739927	Archaea	Methanobacteriota_A	Methanococci	Methanococcales	Methanocaldococcaceae	Methanocaldococcus	*Methanocaldococcus jannaschii*	2190	*Methanocaldococcus jannaschii UBA8849*	https://bacdive.dsmz.de/strain/6981
GCA_000024625.1	Thermophiles		1761737	Archaea	Methanobacteriota_A	Methanococci	Methanococcales	Methanocaldococcaceae	Methanocaldococcus	*Methanocaldococcus vulcanius*	579137	*Methanocaldococcus vulcanius*	https://bacdive.dsmz.de/strain/6983
GCA_000251105.1	Thermophiles		2378438	Archaea	Halobacteriota	Methanocellia	Methanocellales	Methanocellaceae	Methanocella	*Methanocella conradii*	1041930	*Methanocella conradii*	https://doi.org/10.1371/journal.pone.0035279
GCA_000013725.1	Psychrophiles		2575032	Archaea	Halobacteriota	Methanosarcinia	Methanosarcinales	Methanosarcinaceae	Methanococcoides	*Methanococcoides burtonii*	259564	*Methanococcoides burtonii DSM 6242*	https://doi.org/10.1038/ismej.2009.45
GCA_002945325.1	Mesophiles		1714918	Archaea	Methanobacteriota_A	Methanococci	Methanococcales	Methanococcaceae	Methanococcus	*Methanococcus maripaludis*	267377	*Methanococcus maripaludis S2*	https://bacdive.dsmz.de/strain/6991
GCA_000304355.2	Mesophiles		2789774	Archaea	Halobacteriota	Methanomicrobia	Methanomicrobiales	Methanoculleaceae	Methanoculleus	*Methanoculleus bourgensis*	1201294	*Methanoculleus bourgensis*	https://bacdive.dsmz.de/strain/7016
GCA_900095385.1	Mesophiles		2649157	Archaea	Halobacteriota	Methanomicrobia	Methanomicrobiales	Methanoculleaceae	Methanoculleus	*Methanoculleus chikugoensis_A*	1293042	*Methanoculleus chikugoensis*	https://doi.org/10.1099/00207713-51-5-1663
GCA_001602375.1	Mesophiles		2446106	Archaea	Halobacteriota	Methanomicrobia	Methanomicrobiales	Methanoculleaceae	Methanoculleus	*Methanoculleus horonobensis*	528314	*Methanoculleus horonobensis strain T10*	https://doi.org/10.1099/ijs.0.053520-0
GCA_000015825.1	Mesophiles		2478101	Archaea	Halobacteriota	Methanomicrobia	Methanomicrobiales	Methanoculleaceae	Methanoculleus	*Methanoculleus marisnigri*	2198	*Methanoculleus marisnigri isolate 63_41*	https://bacdive.dsmz.de/strain/7020
GCA_002503885.1	Mesophiles		2582043	Archaea	Halobacteriota	Methanomicrobia	Methanomicrobiales	Methanoculleaceae	Methanoculleus	*Methanoculleus marisnigri_B*	368407	*Methanoculleus marisnigri JR1*	https://bacdive.dsmz.de/strain/7020

GCA_001017125.1	Mesophiles		2489717	Archaea	Halobacteriota	Methanomicrobia	Methanomicrobiales	Methanoculleaceae	Methanoculleus	*Methanoculleus sediminis*	1550566	*Methanoculleus sediminis strain S3Fa*	https://doi.org/10.1099/ijs.0.000233
GCA_001571405.1	Thermophiles		2223235	Archaea	Halobacteriota	Methanomicrobia	Methanomicrobiales	Methanoculleaceae	Methanoculleus	*Methanoculleus thermophilus*	2200	*Methanoculleus thermophilus strain CR-1*	https://bacdive.dsmz.de/strain/7022
GCA_004102725.1	Mesophiles		2750720	Archaea	Halobacteriota	Methanomicrobia	Methanomicrobiales	Methanoculleaceae	Methanoculleus_A	*Methanoculleus_A taiwanensis*	1550565	*Methanoculleus taiwanensis strain CYW4*	https://doi.org/10.1099/ijs.0.000062
GCA_000275865.1	Mesophiles		2475100	Archaea	Halobacteriota	Methanomicrobia	Methanomicrobiales	Methanofollaceae	Methanofollis	*Methanofollis liminatans*	28892	*Methanofollis liminatans*	https://www.microbiologyresearch.org/content/journal/ijsem/10.1099/00207713-49-1-247
GCA_009914725.1	Psychrophiles		2189363	Archaea	Halobacteriota	Methanomicrobia	Methanomicrobiales	Methanomicrobiaceae	Methanogenium	*Methanogenium sp009914725*	2599926	*Methanogenium sp. MK-MG*	https://www.nature.com/articles/s41586-019-1916-6
GCA_900215215.1	Thermophiles		1940298	Archaea	Halobacteriota	Methanosarcinia	Methanosarcinales	Methanosarcinaceae	Methanohalophilus	*Methanohalophilus euhalobius*	51203	*Methanohalophilus euhalobius strain DSM 10369*	https://link.springer.com/article/10.1023/A:1000103618451
GCA_001889405.1	Mesophiles		2022959	Archaea	Halobacteriota	Methanosarcinia	Methanosarcinales	Methanosarcinaceae	Methanohalophilus	*Methanohalophilus halophilus*	2177	*Methanohalophilus halophilus strain DSM 3094*	https://bacdive.dsmz.de/strain/7064
GCA_017874375.1	Mesophiles		2116010	Archaea	Halobacteriota	Methanosarcinia	Methanosarcinales	Methanosarcinaceae	Methanohalophilus	*Methanohalophilus levihalophilus*	1431282	*Methanohalophilus levihalophilus*	https://doi.org/10.1099/ijs.0.063677-0
GCA_000025865.1	Mesophiles		2012424	Archaea	Halobacteriota	Methanosarcinia	Methanosarcinales	Methanosarcinaceae	Methanohalophilus	*Methanohalophilus mahii*	547558	*Methanohalophilus mahii DSM 5219*	https://bacdive.dsmz.de/strain/7068
GCA_002761295.1	Mesophiles		2084975	Archaea	Halobacteriota	Methanosarcinia	Methanosarcinales	Methanosarcinaceae	Methanohalophilus	*Methanohalophilus portucalensis*	523843	*Methanohalophilus portucalensis strain FDF-1T chromosome*	https://bacdive.dsmz.de/strain/7074
GCA_004137855.1	Mesophiles		1830088	Archaea	Halobacteriota	Methanosarcinia	Methanosarcinales	Methanosarcinaceae	Methanohalophilus	*Methanohalophilus profundi*	2138083	*Methanohalophilus profundi*	https://doi.org/10.1016/j.syapm.2020.126107
GCA_003722075.1	Mesophiles		1969036	Archaea	Halobacteriota	Methanosarcinia	Methanosarcinales	Methanosarcinaceae	Methanohalophilus	*Methanohalophilus sp003722075*	2485783	*Methanohalophilus sp. RSK*	https://www.frontiersin.org/articles/10.3389/fmicb.2019.00839/full
GCA_000784355.1	Mesophiles		2791704	Archaea	Halobacteriota	Methanomicrobia	Methanomicrobiales	Methanomicrobiaceae	Methanolacinia	*Methanolacinia paynteri*	694436	*Methanolacinia paynteri*	https://bacdive.dsmz.de/strain/7039
GCA_000147875.1	Mesophiles		2843290	Archaea	Halobacteriota	Methanomicrobia	Methanomicrobiales	Methanomicrobiaceae	Methanolacinia	*Methanolacinia petrolearia*	679926	*Methanolacinia petrolearia*	https://bacdive.dsmz.de/strain/7043
GCA_017873855.1	Mesophiles		2662345	Archaea	Halobacteriota	Methanomicrobia	Methanomicrobiales	Methanoregulaceae	Methanolinea_B	*Methanolinea_B mesophila*	547055	*Methanolinea mesophila*	https://doi.org/10.1099/ijs.0.035048-0
GCA_900114835.1	Mesophiles		3107200	Archaea	Halobacteriota	Methanosarcinia	Methanosarcinales	Methanosarcinaceae	Methanolobus	*Methanolobus profundi*	487685	*Methanolobus profundi strain Mob M*	https://doi.org/10.1099/ijs.0.001677-0
GCA_000306725.1	Psychrophiles		3072769	Archaea	Halobacteriota	Methanosarcinia	Methanosarcinales	Methanosarcinaceae	Methanolobus	*Methanolobus psychrophilus*		*Methanolobus psychrophilus R15*	https://journals.asm.org/doi/10.1128/AEM.01146-08
GCA_002243045.1	Psychrophiles		3164721	Archaea	Halobacteriota	Methanosarcinia	Methanosarcinales	Methanosarcinaceae	Methanolobus	*Methanolobus psychrotolerans*	1874706	*Methanolobus psychrotolerans strain YSF-03*	https://doi.org/10.1099/ijsem.0.002685
GCA_004745425.1	Mesophiles	Alkaliphiles	2592212	Archaea	Halobacteriota	Methanosarcinia	Methanosarcinales	Methanosarcinaceae	Methanolobus	*Methanolobus sp004745425*	2052935	*Methanolobus halotolerans*	https://doi.org/10.1099/ijsem.0.004453
GCA_000504205.1	Mesophiles		3151883	Archaea	Halobacteriota	Methanosarcinia	Methanosarcinales	Methanosarcinaceae	Methanolobus	*Methanolobus tindarius*	1090322	*Methanolobus tindarius DSM 2278*	https://bacdive.dsmz.de/strain/7076
GCA_013388255.1	Thermophiles		2704953	Archaea	Halobacteriota	Methanosarcinia	Methanosarcinales	Methanosarcinaceae	Methanolobus	*Methanolobus zinderi*	536044	*Methanolobus zinderi strain DSM 21339*	https://doi.org/10.1099/ijs.0.003772-0
GCA_000711215.1	Mesophiles		1711791	Archaea	Halobacteriota	Methanomicrobia	Methanomicrobiales	Methanomicrobiaceae	Methanomicrobium	*Methanomicrobium mobile*	694440	*Methanomicrobium mobile*	https://bacdive.dsmz.de/strain/7040
GCA_002153915.1	Thermophiles	Alkaliphiles	1513137	Archaea	Halobacteriota	Methanonatronarchaeia	Methanonatronarchaeales	Methanonatronarchaeaceae	Methanonatronarchaeum	*Methanonatronarchaeum thermophilum*	1927129	*Methanonatronarchaeum thermophilum*	https://doi.org/10.1099/ijsem.0.002810
GCA_000243255.1	Mesophiles		3200946	Archaea	Halobacteriota	Methanomicrobia	Methanomicrobiales	Methanomicrobiaceae	Methanoplanus	*Methanoplanus limicola*	937775	*Methanoplanus limicola*	http://standardsingenomics.org/content/9/3/1076/
GCA_000007185.1	Hyperthermophiles		1694969	Archaea	Methanobacteriota_A	Methanopyri	Methanopyrales	Methanopyraceae	Methanopyrus	*Methanopyrus kandleri*	190192	*Methanopyrus kandleri AV19*	https://doi.org/10.1042/bst0320269
GCA_002201895.1	Hyperthermophiles		1421621	Archaea	Methanobacteriota_A	Methanopyri	Methanopyrales	Methanopyraceae	Methanopyrus	*Methanopyrus sp002201895*	1937004	*Methanopyrus sp. KOL6*	https://www.frontiersin.org/articles/10.3389/fmicb.2017.01278/full
GCA_000327485.1	Mesophiles		2820858	Archaea	Halobacteriota	Methanomicrobia	Methanomicrobiales	Methanoregulaceae	Methanoregula	*Methanoregula formicica*	593750	*Methanoregula formicica*	https://doi.org/10.1099/ijs.0.014811-0
GCA_000217995.1		Alkaliphiles	2138444	Archaea	Halobacteriota	Methanosarcinia	Methanosarcinales	Methanosarcinaceae	Methanosalsum	*Methanosalsum zhilinae*	679901	*Methanosalsum zhilinae DSM 4017*	https://doi.org/10.1099/ijsem.0.000488
GCA_000970285.1	Mesophiles		5018607	Archaea	Halobacteriota	Methanosarcinia	Methanosarcinales	Methanosarcinaceae	Methanosarcina	*Methanosarcina horonobensis*	1434110	*Methanosarcina horonobensis*	https://doi.org/10.1099/ijs.0.028548-0
GCA_000970265.1	Psychrophiles		4139808	Archaea	Halobacteriota	Methanosarcinia	Methanosarcinales	Methanosarcinaceae	Methanosarcina	*Methanosarcina lacustris*	1434111	*Methanosarcina lacustris Z-7289*	https://doi.org/10.1078/0723-2020-00058
GCA_000970205.1	Mesophiles		4142816	Archaea	Halobacteriota	Methanosarcinia	Methanosarcinales	Methanosarcinaceae	Methanosarcina	*Methanosarcina mazei*	192952	*Methanosarcina mazei*	https://bacdive.dsmz.de/strain/7096
GCA_000970085.1	Mesophiles		5017558	Archaea	Halobacteriota	Methanosarcinia	Methanosarcinales	Methanosarcinaceae	Methanosarcina	*Methanosarcina siciliae*	1434118	*Methanosarcina siciliae C2J*	https://bacdive.dsmz.de/strain/7083
GCA_002287235.1	Mesophiles		5088600	Archaea	Halobacteriota	Methanosarcinia	Methanosarcinales	Methanosarcinaceae	Methanosarcina	*Methanosarcina spelaei*	1036679	*Methanosarcina spelaei*	https://doi.org/10.1099/ijs.0.064956-0
GCA_000969885.1	Thermophiles		3127379	Archaea	Halobacteriota	Methanosarcinia	Methanosarcinales	Methanosarcinaceae	Methanosarcina	*Methanosarcina thermophila*	523844	*Methanosarcina thermophila TM-1*	https://bacdive.dsmz.de/strain/7121
GCA_000969905.1	Mesophiles		4563885	Archaea	Halobacteriota	Methanosarcinia	Methanosarcinales	Methanosarcinaceae	Methanosarcina	*Methanosarcina vacuolata*	1434123	*Methanosarcina vacuolata Z-761*	https://bacdive.dsmz.de/strain/7126
GCA_000021965.1	Mesophiles		2922917	Archaea	Halobacteriota	Methanomicrobia	Methanomicrobiales	Methanosphaerulaceae	Methanosphaerula	*Methanosphaerula palustris*	521011	*Methanosphaerula palustris*	https://doi.org/10.1099/ijs.0.006890-0
GCA_000013445.1	Mesophiles		3544738	Archaea	Halobacteriota	Methanomicrobia	Methanomicrobiales	Methanospirillaceae	Methanospirillum	*Methanospirillum hungatei*	323259	*Methanospirillum hungatei*	https://bacdive.dsmz.de/strain/7132
GCA_019263745.1	Mesophiles		3393136	Archaea	Halobacteriota	Methanomicrobia	Methanomicrobiales	Methanospirillaceae	Methanospirillum	*Methanospirillum sp012729995*	323259	*Methanospirillum hungatei*	https://bacdive.dsmz.de/strain/7132
GCA_003173335.1	Psychrophiles		3740742	Archaea	Halobacteriota	Methanomicrobia	Methanomicrobiales	Methanospirillaceae	Methanospirillum	*Methanospirillum stamsii*	1277351	*Methanospirillum stamsii*	https://doi.org/10.1099/ijs.0.056218-0
GCA_000145295.1	Thermophiles		1639135	Archaea	Methanobacteriota	Methanobacteria	Methanobacteriales	Methanothermobacteraceae	Methanothermobacter	*Methanothermobacter marburgensis*	2603820	*Methanothermobacter sp. KEPCO-1*	https://doi.org/10.1016/j.enzmictec.2022.110067
GCA_000828575.1	Thermophiles		1731018	Archaea	Methanobacteriota	Methanobacteria	Methanobacteriales	Methanothermobacteraceae	Methanothermobacter	*Methanothermobacter sp000828575*	866790	*Methanothermobacter sp. CaT2*	https://doi.org/10.1128/genomea.00672-13
GCA_000008645.1	Thermophiles		1751377	Archaea	Methanobacteriota	Methanobacteria	Methanobacteriales	Methanothermobacteraceae	Methanothermobacter	*Methanothermobacter thermautotrophicus*	187420	*Methanothermobacter thermautotrophicus str. Delta H*	https://bacdive.dsmz.de/strain/6882
GCA_900095815.1	Thermophiles		1686891	Archaea	Methanobacteriota	Methanobacteria	Methanobacteriales	Methanothermobacteraceae	Methanothermobacter	*Methanothermobacter wolfeii*	145261	*Methanothermobacter wolfeii SIV6*	https://doi.org/10.1099/00207713-50-1-43
GCA_003264935.1	Thermophiles		1467867	Archaea	Methanobacteriota	Methanobacteria	Methanobacteriales	Methanothermobacteraceae_A	Methanothermobacter_A	*Methanothermobacter_A tenebrarum*	680118	*Methanothermobacter tenebrarum*	https://doi.org/10.1099/ijs.0.041681-0
GCA_000017185.1	Mesophiles		1569500	Archaea	Methanobacteriota_A	Methanococci	Methanococcales	Methanococcaceae	Methanothermococcus_A	*Methanothermococcus_A aeolicus*	42879	*Methanococcus aeolicus*	https://doi.org/10.1099/ijs.0.64216-0
GCA_000179575.2	Thermophiles		1677455	Archaea	Methanobacteriota_A	Methanococci	Methanococcales	Methanococcaceae	Methanothermococcus_A	*Methanothermococcus_A okinawensis*	647113	*Methanothermococcus okinawensis IH1*	https://doi.org/10.1099/00207713-52-4-1089
GCA_000166095.1	Hyperthermophiles		1243342	Archaea	Methanobacteriota	Methanobacteria	Methanobacteriales	Methanothermaceae	Methanothermus	*Methanothermus fervidus*	523846	*Methanothermus fervidus V24S, DSM 2088*	https://doi.org/10.1128/jb.174.11.3508-3513.1992
GCA_002502785.1	Mesophiles		2512329	Archaea	Halobacteriota	Methanosarcinia	Methanotrichales	Methanotrichaceae	Methanothrix_A	*Methanothrix_A harundinacea_A*	1110509	*Methanosaeta harundinacea*	https://bacdive.dsmz.de/strain/131756
GCA_002506535.1	Mesophiles		2342380	Archaea	Halobacteriota	Methanosarcinia	Methanotrichales	Methanotrichaceae	Methanothrix_A	*Methanothrix_A harundinacea_B*	1110509	*Methanosaeta harundinacea*	https://bacdive.dsmz.de/strain/131756
GCA_001509375.1	Mesophiles		2382964	Archaea	Halobacteriota	Methanosarcinia	Methanotrichales	Methanotrichaceae	Methanothrix_A	*Methanothrix_A harundinacea_D*	1110509	*Methanosaeta harundinacea*	https://bacdive.dsmz.de/strain/131756
GCA_000235565.1	Mesophiles		2571034	Archaea	Halobacteriota	Methanosarcinia	Methanotrichales	Methanotrichaceae	Methanothrix_A	*Methanothrix_A harundinacea_E*	1110509	*Methanosaeta harundinacea*	https://bacdive.dsmz.de/strain/131756
GCA_000014945.1	Thermophiles		1879471	Archaea	Halobacteriota	Methanosarcinia	Methanotrichales	Methanotrichaceae	Methanothrix_B	*Methanothrix_B thermoacetophila*	349307	*Methanothrix thermoacetophila*	https://doi.org/10.1099/00207713-42-3-463
GCA_000243455.2	Thermophiles		1818783	Archaea	Methanobacteriota_A	Methanococci	Methanococcales	Methanococcaceae	Methanotorris	*Methanotorris formicicus*	647171	*Methanotorris formicicus Mc-S-70*	https://doi.org/10.1099/ijs.0.02887-0
GCA_000214415.1	Hyperthermophiles		1854197	Archaea	Methanobacteriota_A	Methanococci	Methanococcales	Methanococcaceae	Methanotorris	*Methanotorris igneus*	880724	*Methanotorris igneus Kol 5*	https://doi.org/10.1038/s42003-021-01828-5
GCA_902143385.2		Acidophiles	2276790	Bacteria	Verrucomicrobiota	Verrucomicrobiae	Methylacidiphilales	Methylacidiphilaceae	Methylacidimicrobium	*Methylacidimicrobium cyclopophantes*	1041766	*Methylacidimicrobium cyclopophantes*	https://doi.org/10.1128/mra.00315-20
GCA_000953475.1	Thermophiles	Acidophiles	2476671	Bacteria	Verrucomicrobiota	Verrucomicrobiae	Methylacidiphilales	Methylacidiphilaceae	Methylacidiphilum	*Methylacidiphilum fumariolicum*	591154	*Methylacidiphilum fumariolicum*	https://doi.org/10.1038/ismej.2016.171
GCA_007475525.1	Thermophiles	Acidophiles	2202032	Bacteria	Verrucomicrobiota	Verrucomicrobiae	Methylacidiphilales	Methylacidiphilaceae	Methylacidiphilum	*Methylacidiphilum kamchatkense*	1202785	*Methylacidiphilum kamchatkense Kam1*	https://doi.org/10.1128/genomea.00065-15

GCA_004421185.1		Acidophiles	2250350	Bacteria	Verrucomicrobiota	Verrucomicrobiae	Methylacidiphilales	Methylacidiphilaceae	Methylacidiphilum	Methylacidiphilum sp004421185	1847730	Methylacidiphilum sp. Yel	https://www.mdpi.com/2076-2607/10/1/142
GCA_017310505.1	Thermophiles	Acidophiles	2254698	Bacteria	Verrucomicrobiota	Verrucomicrobiae	Methylacidiphilales	Methylacidiphilaceae	Methylacidiphilum	Methylacidiphilum sp004421255	1847729	Methylacidiphilum sp. Phi	https://biotechnologyforbiofuels.biomedcentral.com/articles/10.1186/s13068-022-02105-1#Bib1
GCA_000968535.1		Alkaliphiles	4796711	Bacteria	Proteobacteria	Gammaproteobacteria	Methylococcales	Methylomonadaceae	Methylotuvimicrobium	Methylotuvimicrobium alcaliphilum	1091494	Methylotuvimicrobium alcaliphilum 20Z	https://doi.org/10.1007/s00792-021-01228-x
GCA_004216855.1		Alkaliphiles	2687802	Bacteria	Actinobacteriota	Actinomycetia	Actinomycetales	Microbacteriaceae	Microcella	Microcella alkaliphila	279828	Microcella alkaliphila	https://doi.org/10.1099/ijs.0.64320-0
GCA_002355395.1		Alkaliphiles	2702837	Bacteria	Actinobacteriota	Actinomycetia	Actinomycetales	Microbacteriaceae	Microcella	Microcella alkaliphila_A	279828	Microcella alkaliphila	https://doi.org/10.1099/ijs.0.64320-0
GCA_004216575.1		Alkaliphiles	2544518	Bacteria	Actinobacteriota	Actinomycetia	Actinomycetales	Microbacteriaceae	Microcella	Microcella putealis	337005	Microcella putealis	https://doi.org/10.1016/j.syapm.2005.03.004
GCA_009735625.1	Thermophiles		3559563	Bacteria	Firmicutes_B	Moorellia	Moorellales	Moorellaceae	Moorella	Moorella glycerini	55779	Moorella glycerini	https://doi.org/10.1099/00207713-47-4-969
GCA_002995755.1	Thermophiles		2628568	Bacteria	Firmicutes_B	Moorellia	Moorellales	Moorellaceae	Moorella	Moorella humiferrea	676965	Moorella humiferrea	https://doi.org/10.1099/ijs.0.029009-0
GCA_001594015.1	Thermophiles		2999839	Bacteria	Firmicutes_B	Moorellia	Moorellales	Moorellaceae	Moorella	Moorella mulderi	1122241	Moorella mulderi DSM 14980	https://doi.org/10.1007/s00203-003-0523-x
GCA_002995805.1	Thermophiles		3328173	Bacteria	Firmicutes_B	Moorellia	Moorellales	Moorellaceae	Moorella	Moorella stamsii	1266720	Moorella stamsii	https://doi.org/10.1099/ijs.0.050369-0
GCA_001267405.1	Thermophiles		2527564	Bacteria	Firmicutes_B	Moorellia	Moorellales	Moorellaceae	Moorella	Moorella thermoacetica	1325331	Moorella thermoacetica Y72	https://doi.org/10.1016/j.resmic.2004.10.002
GCA_000276805.1	Psychrophiles		4889582	Bacteria	Proteobacteria	Gammaproteobacteria	Enterobacterales	Moritellaceae	Moritella	Moritella dasanensis	1201293	Moritella dasanensis ArB 0140	https://doi.org/10.1099/ijs.0.65501-0
GCA_008931805.1	Psychrophiles		4760425	Bacteria	Proteobacteria	Gammaproteobacteria	Enterobacterales	Moritellaceae	Moritella	Moritella marina	1202962	Moritella marina ATCC 15381	https://doi.org/10.1128/jb.01382-12
GCA_000953735.1	Psychrophiles		5093989	Bacteria	Proteobacteria	Gammaproteobacteria	Enterobacterales	Moritellaceae	Moritella	Moritella viscosa	80854	Moritella viscosa	https://doi.org/10.1016/j.carres.2014.10.007
GCA_900465055.1	Psychrophiles		4433651	Bacteria	Proteobacteria	Gammaproteobacteria	Enterobacterales	Moritellaceae	Moritella	Moritella yayanosii	69539	Moritella yayanosii	https://www.microbiologyresearch.org/content/journal/mgen/10.1099/mgen.0.000591
GCA_002156705.1		Alkaliphiles	3930546	Archaea	Halobacteriota	Halobacteria	Halobacteriales	Natrialbaceae	Natrarchaeobaculum	Natrarchaeobaculum aegyptiacum	745377	Natrarchaeobaculum aegyptiacum	https://doi.org/10.1099/ijsem.0.004186
GCA_003430825.1		Alkaliphiles	3789323	Archaea	Halobacteriota	Halobacteria	Halobacteriales	Natrialbaceae	Natrarchaeobaculum	Natrarchaeobaculum sulfurireducens	2044521	Natrarchaeobaculum sulfurireducens	https://doi.org/10.1099/ijsem.0.004186
GCA_003841505.1		Alkaliphiles	4566486	Archaea	Halobacteriota	Halobacteria	Halobacteriales	Natrialbaceae	Natrarchaeobius	Natrarchaeobius chitinivorans	1679083	Natrarchaeobius chitinivorans	https://doi.org/10.1016/j.syapm.2019.01.001
GCA_003841465.1		Alkaliphiles	4614480	Archaea	Halobacteriota	Halobacteria	Halobacteriales	Natrialbaceae	Natrarchaeobius	Natrarchaeobius chitinivorans_A	1679083	Natrarchaeobius chitinivorans	https://doi.org/10.1016/j.syapm.2019.01.001
GCA_008245225.1		Alkaliphiles	4201486	Archaea	Halobacteriota	Halobacteria	Halobacteriales	Natrialbaceae	Natrarchaeobius	Natrarchaeobius swarupiae	2448032	Natrialba swarupiae	https://doi.org/10.1099/ijsem.0.003986
GCA_000337555.1	Mesophiles		4404175	Archaea	Halobacteriota	Halobacteria	Halobacteriales	Natrialbaceae	Natrialba	Natrialba asiatica	29540	Natrialba asiatica DSM 12278	https://www.microbiologyresearch.org/content/journal/ijsem/10.1099/00207713-51-3-1133
GCA_000337135.1	Mesophiles	Alkaliphiles	4309274	Archaea	Halobacteriota	Halobacteria	Halobacteriales	Natrialbaceae	Natrialba	Natrialba chahannaoensis	1227492	Natrialba chahannaoensis JCM 10990	https://doi.org/10.1099/00207713-51-5-1693
GCA_000337575.1	Mesophiles	Alkaliphiles	4159606	Archaea	Halobacteriota	Halobacteria	Halobacteriales	Natrialbaceae	Natrialba	Natrialba hulunbeirensis	1227493	Natrialba hulunbeirensis JCM 10989	https://doi.org/10.1099/00207713-51-5-1693
GCA_000025625.1		Alkaliphiles	4443643	Archaea	Halobacteriota	Halobacteria	Halobacteriales	Natrialbaceae	Natrialba	Natrialba magadii	547559	Natrialba magadii ATCC 43099	https://doi.org/10.1099/00207713-47-3-853
GCA_004217335.1	Thermophiles		4256545	Archaea	Halobacteriota	Halobacteria	Halobacteriales	Natrialbaceae	Natrinema	Natrinema hispanicum	392421	Haloterrigena hispanica	https://doi.org/10.1099/ijs.0.64895-0
GCA_900111485.1	Thermophiles		3963480	Archaea	Halobacteriota	Halobacteria	Halobacteriales	Natrialbaceae	Natrinema	Natrinema hispanicum_A	392421	Haloterrigena hispanica	https://doi.org/10.1099/ijs.0.64895-0
GCA_000337475.1	Thermophiles		3522035	Archaea	Halobacteriota	Halobacteria	Halobacteriales	Natrialbaceae	Natrinema	Natrinema limicola	1230457	Haloterrigena limicola	https://www.microbiologyresearch.org/content/journal/ijsem/10.1099/ijs.0.64372-0
GCA_000690595.2	Thermophiles		3794337	Archaea	Halobacteriota	Halobacteria	Halobacteriales	Natrialbaceae	Natrinema	Natrinema mahii	1416969	Haloterrigena mahii	https://doi.org/10.1099/ijsem.0.001811
GCA_001953745.1	Thermophiles		3980616	Archaea	Halobacteriota	Halobacteria	Halobacteriales	Natrialbaceae	Natrinema	Natrinema saccharevitans	301967	Haloterrigena saccharevitans	https://doi.org/10.1099/ijs.0.63761-0
GCA_900110865.1	Mesophiles		4857017	Archaea	Halobacteriota	Halobacteria	Halobacteriales	Natrialbaceae	Natrinema	Natrinema salaciae	1186196	Natrinema salaciae	https://doi.org/10.1016/j.syapm.2012.06.005
GCA_900110455.1	Mesophiles		4272165	Archaea	Halobacteriota	Halobacteria	Halobacteriales	Natrialbaceae	Natrinema	Natrinema salifodinae	1202768	Halopiger salifodinae	https://doi.org/10.1099/ijs.0.050971-0
GCA_002572525.1	Thermophiles		5058058	Archaea	Halobacteriota	Halobacteria	Halobacteriales	Natrialbaceae	Natrinema	Natrinema sp002572525	1608465	Natrinema sp. CBA1119	https://www.nature.com/articles/s41598-018-25887-7
GCA_900215575.1	Mesophiles		3164179	Archaea	Halobacteriota	Halobacteria	Halobacteriales	Natronoarchaeaceae	Natronoarchaeum	Natronoarchaeum philippinense	558529	Natronoarchaeum philippinense	https://doi.org/10.1099/ijs.0.042549-0
GCA_000230715.3		Alkaliphiles	3788356	Archaea	Halobacteriota	Halobacteria	Halobacteriales	Natrialbaceae	Natronobacterium	Natronobacterium gregoryi	797304	Natronobacterium gregoryi SP2	https://doi.org/10.1099/00207713-47-3-853
GCA_900104065.1	Mesophiles	Alkaliphiles	4009868	Archaea	Halobacteriota	Halobacteria	Halobacteriales	Natrialbaceae	Natronobacterium	Natronobacterium texcoconense	1095778	Natronobacterium texcoconense	https://doi.org/10.1099/ijs.0.053629-0
GCA_000337675.1		Alkaliphiles	4416525	Archaea	Halobacteriota	Halobacteria	Halobacteriales	Natrialbaceae	Natronococcus	Natronococcus amylolyticus	1227497	Natronococcus amylolyticus DSM 10524	https://doi.org/10.1099/00207713-45-4-762
GCA_000337695.1	Mesophiles		4496185	Archaea	Halobacteriota	Halobacteria	Halobacteriales	Natrialbaceae	Natronococcus	Natronococcus jeotgali	1227498	Natronococcus jeotgali	https://doi.org/10.1099/ijs.0.65120-0
GCA_000328685.1		Alkaliphiles	4314118	Archaea	Halobacteriota	Halobacteria	Halobacteriales	Natrialbaceae	Natronococcus	Natronococcus occultus	694430	Natronococcus occultus SP4	https://doi.org/10.1002/1521-4028(200112)41:6%3C375::aid-jobm375%3E3.0.co;2-0
GCA_008122205.1		Alkaliphiles	5316806	Archaea	Halobacteriota	Halobacteria	Halobacteriales	Natrialbaceae	Natronococcus	Natronococcus sp008122205	2055836	Natronococcus pandeyae	https://doi.org/10.1007/s00284-021-02740-1
GCA_000026045.1		Alkaliphiles	2749696	Archaea	Halobacteriota	Halobacteria	Halobacteriales	Haloarculaceae	Natronomonas	Natronomonas pharaonis	348780	Natronomonas pharaonis DSM 2160	https://doi.org/10.1099/00207713-47-3-853
GCA_009392895.1		Alkaliphiles	4348483	Archaea	Halobacteriota	Halobacteria	Halobacteriales	Natrialbaceae	Natronorubrum	Natronorubrum aibiense	348826	Natronorubrum aibiense	https://doi.org/10.1099/ijs.0.64222-0
GCA_001971705.1	Mesophiles	Alkaliphiles	3835796	Archaea	Halobacteriota	Halobacteria	Halobacteriales	Natrialbaceae	Natronorubrum	Natronorubrum daqingense	588898	Haloterrigena daqingensis	https://doi.org/10.1099/ijs.0.013995-0
GCA_900108095.1		Alkaliphiles	3782545	Archaea	Halobacteriota	Halobacteria	Halobacteriales	Natrialbaceae	Natronorubrum	Natronorubrum sediminis	640943	Natronorubrum sediminis	https://doi.org/10.1099/ijs.0.015602-0
GCA_000337735.1	Thermophiles	Alkaliphiles	3460288	Archaea	Halobacteriota	Halobacteria	Halobacteriales	Natrialbaceae	Natronorubrum	Natronorubrum sulfidifaciens	1230460	Natronorubrum sulfidifaciens	https://doi.org/10.1099/ijs.0.64651-0
GCA_000383975.1		Alkaliphiles	4934841	Archaea	Halobacteriota	Halobacteria	Halobacteriales	Natrialbaceae	Natronorubrum	Natronorubrum tibetense	1114856	Natronorubrum tibetense GA33	https://doi.org/10.1099/00207713-49-1-261
GCA_000220175.2	Mesophiles		1607695	Archaea	Thermoproteota	Nitrososphaeria	Nitrososphaerales	Nitrosopumilaceae	Nitrosarchaeum	Nitrosarchaeum koreense	1088740	Nitrosarchaeum koreense MY1	https://www.microbiologyresearch.org/content/journal/ijsem/10.1099/ijsem.0.002926
GCA_000956175.1	Mesophiles		1803090	Archaea	Thermoproteota	Nitrososphaeria	Nitrososphaerales	Nitrosopumilaceae	Nitrosopumilus	Nitrosopumilus adriaticus	1580092	Nitrosopumilus adriaticus	https://doi.org/10.1099/ijsem.0.003360
GCA_000018465.1	Mesophiles		1645259	Archaea	Thermoproteota	Nitrososphaeria	Nitrososphaerales	Nitrosopumilaceae	Nitrosopumilus	Nitrosopumilus maritimus	436308	Nitrosopumilus maritimus SCM1	10.1099/ijsem.0.002416
GCA_000875775.1	Mesophiles		1713078	Archaea	Thermoproteota	Nitrososphaeria	Nitrososphaerales	Nitrosopumilaceae	Nitrosopumilus	Nitrosopumilus piranensis	1582439	Nitrosopumilus piranensis	https://doi.org/10.1099/ijsem.0.003360
GCA_000698785.1	Mesophiles		2527938	Archaea	Thermoproteota	Nitrososphaeria	Nitrososphaerales	Nitrososphaeraceae	Nitrososphaera	Nitrososphaera viennensis	926571	Nitrososphaera viennensis EN76	https://doi.org/10.1099/ijs.0.063172-0
GCA_000967895.1	Psychrophiles		4406383	Bacteria	Proteobacteria	Gammaproteobacteria	Pseudomonadales	DSM-6294	Oleispira	Oleispira antarctica	698738	Oleispira antarctica RB-8	https://www.microbiologyresearch.org/content/journal/ijsem/10.1099/ijs.0.02366-0
GCA_000966265.1	Hyperthermophiles		2206431	Archaea	Methanobacteriota_B	Thermococci	Thermococcales	Thermococcaceae	Palaeococcus	Palaeococcus ferrophilus	588319	Palaeococcus ferrophilus DSM 13482	https://doi.org/10.1099/00207713-50-2-489
GCA_000725425.1	Thermophiles		1859370	Archaea	Methanobacteriota_B	Thermococci	Thermococcales	Thermococcaceae	Palaeococcus	Palaeococcus pacificus	1343739	Palaeococcus pacificus DY20341	https://doi.org/10.1099/ijs.0.044487-0
GCA_900111865.1	Thermophiles		3448881	Bacteria	Firmicutes	Bacilli	Bacillales	Anoxybacillaceae	Parageobacillus	Parageobacillus thermantarcticus	186116	Parageobacillus thermantarcticus	https://bacdive.dsmz.de/strain/1438
GCA_001295365.1	Thermophiles		3873116	Bacteria	Firmicutes	Bacilli	Bacillales	Anoxybacillaceae	Parageobacillus	Parageobacillus thermoglucosidasius	1136178	Parageobacillus thermoglucosidasius TNO-09.020	https://bacdive.dsmz.de/strain/1430
GCA_001029445.1		Alkaliphiles	5078735	Bacteria	Proteobacteria	Gammaproteobacteria	Enterobacterales	Vibrionaceae	Photobacterium	Photobacterium aquae	1195763	Photobacterium aquae	https://doi.org/10.1099/ijs.0.055020-0
GCA_002954455.1	Psychrophiles		4525475	Bacteria	Proteobacteria	Gammaproteobacteria	Enterobacterales	Vibrionaceae	Photobacterium	Photobacterium aquimaris	512643	Photobacterium aquimaris	https://www.microbiologyresearch.org/content/journal/ijsem/10.1099/ijs.0.004309-0
GCA_002849605.1	Psychrophiles		4559543	Bacteria	Proteobacteria	Gammaproteobacteria	Enterobacterales	Vibrionaceae	Photobacterium	Photobacterium carnosum	2023717	Photobacterium carnosum	https://www.sciencedirect.com/science/article/pii/S0723202017301686?via%3Dihub
GCA_003026815.1	Psychrophiles		4914916	Bacteria	Proteobacteria	Gammaproteobacteria	Enterobacterales	Vibrionaceae	Photobacterium	Photobacterium damselae	38293	Photobacterium damselae	https://www.frontiersin.org/articles/10.3389/fmicb.2013.00283/full
GCA_003025615.1	Psychrophiles		6446463	Bacteria	Proteobacteria	Gammaproteobacteria	Enterobacterales	Vibrionaceae	Photobacterium	Photobacterium frigidiphilum	264736	Photobacterium frigidiphilum	https://doi.org/10.1099/ijs.0.63338-0
GCA_003025595.1		Alkaliphiles	5533005	Bacteria	Proteobacteria	Gammaproteobacteria	Enterobacterales	Vibrionaceae	Photobacterium	Photobacterium ganghwense	320778	Photobacterium ganghwense	https://www.microbiologyresearch.org/content/journal/ijsem/10.1099/ijs.0.63811-0

GCA_000425165.1	Psychrophiles		4690166	Bacteria	Proteobacteria	Gammaproteobacteria	Enterobacterales	Vibrionaceae	Photobacterium	*Photobacterium halotolerans*	1122959	*Photobacterium halotolerans DSM 18316*	https://www.microbiologyresearch.org/content/journal/ijsem/10.1099/ijs.0.64099-0
GCA_003026395.1	Psychrophiles		4308695	Bacteria	Proteobacteria	Gammaproteobacteria	Enterobacterales	Vibrionaceae	Photobacterium	*Photobacterium iliopiscarium*	56192	*Photobacterium iliopiscarium*	https://bacdive.dsmz.de/strain/17224
GCA_003026355.1	Psychrophiles		4732354	Bacteria	Proteobacteria	Gammaproteobacteria	Enterobacterales	Vibrionaceae	Photobacterium	*Photobacterium kishitanii*	318456	*Photobacterium kishitanii*	https://bacdive.dsmz.de/strain/17235
GCA_003026475.1	Psychrophiles		4943313	Bacteria	Proteobacteria	Gammaproteobacteria	Enterobacterales	Vibrionaceae	Photobacterium	*Photobacterium lipolyticum*	266810	*Photobacterium lipolyticum*	https://www.microbiologyresearch.org/content/journal/ijsem/10.1099/ijs.0.63215-0
GCA_900166965.1	Psychrophiles		4525300	Bacteria	Proteobacteria	Gammaproteobacteria	Enterobacterales	Vibrionaceae	Photobacterium	*Photobacterium piscicola*	1378299	*Photobacterium piscicola*	https://www.sciencedirect.com/science/article/pii/S0723202014000770?via%3Dihub
GCA_003026285.1	Psychrophiles		6116719	Bacteria	Proteobacteria	Gammaproteobacteria	Enterobacterales	Vibrionaceae	Photobacterium	*Photobacterium profundum*	314280	*Photobacterium profundum 3TCK*	https://doi.org/10.1007/s007920050036
GCA_001939735.1	Psychrophiles		6484429	Bacteria	Proteobacteria	Gammaproteobacteria	Enterobacterales	Vibrionaceae	Photobacterium	*Photobacterium proteolyticum*	1903952	*Photobacterium proteolyticum*	https://www.microbiologyresearch.org/content/journal/ijsem/10.1099/ijsem.0.001873
GCA_001077885.1	Psychrophiles		5523519	Bacteria	Proteobacteria	Gammaproteobacteria	Enterobacterales	Vibrionaceae	Photobacterium	*Photobacterium swingsii*	680026	*Photobacterium swingsii*	https://www.microbiologyresearch.org/content/journal/ijsem/10.1099/ijs.0.019687-0
GCA_900166975.1	Psychrophiles		4420947	Bacteria	Proteobacteria	Gammaproteobacteria	Enterobacterales	Vibrionaceae	Photobacterium	*Photobacterium toruni*	1935446	*Photobacterium toruni*	https://www.microbiologyresearch.org/content/journal/ijsem/10.1099/ijsem.0.002325
GCA_900176435.1	Thermophiles	Acidophiles	1534155	Archaea	Thermoplasmatota	Thermoplasmata	Thermoplasmatales	Thermoplasmataceae	Picrophilus	*Picrophilus oshimae*	1122961	*Picrophilus oshimae DSM 9789*	https://doi.org/10.1023/a:1020525525490
GCA_003719725.1		Alkaliphiles	3335228	Bacteria	Firmicutes	Bacilli	Bacillales_A	Planococcaceae	Planococcus	*Planococcus salinus*	1848460	*Planococcus salinus*	https://doi.org/10.1099/ijsem.0.002548
GCA_001640115.1	Psychrophiles		3942702	Bacteria	Bacteroidota	Bacteroidia	Flavobacteriales	Flavobacteriaceae	Polaribacter	*Polaribacter atrinae*	1333662	*Polaribacter atrinae*	https://www.microbiologyresearch.org/content/journal/ijsem/10.1099/ijs.0.060889-0
GCA_002954665.1	Psychrophiles		4064562	Bacteria	Bacteroidota	Bacteroidia	Flavobacteriales	Flavobacteriaceae	Polaribacter	*Polaribacter glomeratus*	102	*Polaribacter glomeratus*	https://bacdive.dsmz.de/strain/136694
GCA_014784055.1	Psychrophiles		3800626	Bacteria	Bacteroidota	Bacteroidia	Flavobacteriales	Flavobacteriaceae	Polaribacter	*Polaribacter haliotis*	1888915	*Polaribacter haliotis*	https://pubmed.ncbi.nlm.nih.gov/27902190/
GCA_000153225.1	Psychrophiles		2763458	Bacteria	Bacteroidota	Bacteroidia	Flavobacteriales	Flavobacteriaceae	Polaribacter	*Polaribacter irgensii*	313594	*Polaribacter irgensii 23-P*	https://www.microbiologyresearch.org/content/journal/ijsem/10.1099/00207713-48-1-223
GCA_002954685.1	Psychrophiles		3904103	Bacteria	Bacteroidota	Bacteroidia	Flavobacteriales	Flavobacteriaceae	Polaribacter	*Polaribacter porphyrae*	1137780	*Polaribacter porphyrae*	https://www.microbiologyresearch.org/content/journal/ijsem/10.1099/ijs.0.041434-0
GCA_001975665.1	Psychrophiles	Alkaliphiles	4125014	Bacteria	Bacteroidota	Bacteroidia	Flavobacteriales	Flavobacteriaceae	Polaribacter	*Polaribacter reichenbachii*	996801	*Polaribacter reichenbachii*	https://link.springer.com/article/10.1007/s00284-012-0200-x
GCA_001761365.1	Psychrophiles	Alkaliphiles	3809314	Bacteria	Bacteroidota	Bacteroidia	Flavobacteriales	Flavobacteriaceae	Polaribacter	*Polaribacter vadi*	1774273	*Polaribacter vadi*	https://doi.org/10.1099/ijsem.0.001591
GCA_018861005.1	Psychrophiles	Alkaliphiles	3934366	Bacteria	Bacteroidota	Bacteroidia	Flavobacteriales	Flavobacteriaceae	Polaribacter	*Polaribacter vadi_A*	1774273	*Polaribacter vadi*	https://doi.org/10.1099/ijsem.0.001591
GCA_000709345.1	Psychrophiles		5284042	Bacteria	Proteobacteria	Gammaproteobacteria	Burkholderiales	Burkholderiaceae	Polaromonas	*Polaromonas glacialis*	866564	*Polaromonas glacialis*	https://doi.org/10.1099/ijs.0.037556-0
GCA_001598235.1	Psychrophiles		5134650	Bacteria	Proteobacteria	Gammaproteobacteria	Burkholderiales	Burkholderiaceae	Polaromonas	*Polaromonas jejuensis*	1321608	*Polaromonas jejuensis NBRC 106434*	https://www.microbiologyresearch.org/content/journal/ijsem/10.1099/ijs.0.65529-0
GCA_000015505.1	Psychrophiles		5366143	Bacteria	Proteobacteria	Gammaproteobacteria	Burkholderiales	Burkholderiaceae	Polaromonas	*Polaromonas naphthalenivorans*	365044	*Polaromonas naphthalenivorans CJ2*	https://pubmed.ncbi.nlm.nih.gov/14742464/
GCA_000507185.2	Hyperthermophiles		6150048	Bacteria	Proteobacteria	Gammaproteobacteria	Pseudomonadales	Pseudomonadaceae	Pseudomonas_E	*Pseudomonas_E syringae*	52001	*Stetteria hydrogenophila*	https://bacdive.dsmz.de/strain/4193
GCA_000217815.1	Thermophiles		2039943	Bacteria	Thermotogota	Thermotogae	Thermotogales	DSM-5069	Pseudothermotoga	*Pseudothermotoga thermarum*	688269	*Pseudothermotoga thermarum DSM 5069*	https://link.springer.com/article/10.1007/s10482-013-0062-7
GCA_000828655.1	Thermophiles		2014912	Bacteria	Thermotogota	Thermotogae	Thermotogales	DSM-5069	Pseudothermotoga_A	*Pseudothermotoga_A caldifontis*	1408159	*Thermotoga caldifontis AZM44c09*	https://doi.org/10.1099/ijs.0.060137-0
GCA_000816145.1	Thermophiles		2165416	Bacteria	Thermotogota	Thermotogae	Thermotogales	DSM-5069	Pseudothermotoga_A	*Pseudothermotoga_A hypogea*	1123384	*Pseudothermotoga hypogea DSM 11164 = NBRC 106472*	https://link.springer.com/article/10.1007/s10482-013-0062-7
GCA_000504085.1	Thermophiles		2169860	Bacteria	Thermotogota	Thermotogae	Thermotogales	DSM-5069	Pseudothermotoga_B	*Pseudothermotoga_B elfii*	416591	*Pseudothermotoga lettingae TMO*	https://link.springer.com/article/10.1007/s10482-013-0062-7
GCA_000828675.1	Thermophiles		2187612	Bacteria	Thermotogota	Thermotogae	Thermotogales	DSM-5069	Pseudothermotoga_B	*Pseudothermotoga_B profunda*	1408160	*Thermotoga profunda AZM34c06*	https://doi.org/10.1099/ijs.0.060137-0
GCA_001606025.1	Psychrophiles		3349444	Bacteria	Proteobacteria	Gammaproteobacteria	Pseudomonadales	Moraxellaceae	Psychrobacter	*Psychrobacter alimentarius_A*	261164	*Psychrobacter alimentarius*	https://pubmed.ncbi.nlm.nih.gov/15653872/
GCA_000471625.1	Psychrophiles		3216409	Bacteria	Proteobacteria	Gammaproteobacteria	Pseudomonadales	Moraxellaceae	Psychrobacter	*Psychrobacter aquaticus*	1354303	*Psychrobacter aquaticus CMS 56*	https://doi.org/10.1099/ijs.0.03030-0
GCA_000012305.1	Psychrophiles		2650701	Bacteria	Proteobacteria	Gammaproteobacteria	Pseudomonadales	Moraxellaceae	Psychrobacter	*Psychrobacter arcticus*	259536	*Psychrobacter arcticus 273-4*	https://doi.org/10.1099/ijs.0.64043-0
GCA_016107535.1	Psychrophiles		3242921	Bacteria	Proteobacteria	Gammaproteobacteria	Pseudomonadales	Moraxellaceae	Psychrobacter	*Psychrobacter cibarius*	282669	*Psychrobacter cibarius*	https://doi.org/10.1099/ijs.0.63398-0
GCA_000013905.1	Psychrophiles		3101097	Bacteria	Proteobacteria	Gammaproteobacteria	Pseudomonadales	Moraxellaceae	Psychrobacter	*Psychrobacter cryohalolentis*	330922	*Psychrobacter cryohalolentis*	https://doi.org/10.1099/ijs.0.64043-0
GCA_003217155.1	Psychrophiles		3499844	Bacteria	Proteobacteria	Gammaproteobacteria	Pseudomonadales	Moraxellaceae	Psychrobacter	*Psychrobacter fozii*	198480	*Psychrobacter fozii*	https://doi.org/10.1099/ijs.0.02457-0
GCA_007997305.1	Psychrophiles		2846672	Bacteria	Proteobacteria	Gammaproteobacteria	Pseudomonadales	Moraxellaceae	Psychrobacter	*Psychrobacter frigidicola*	45611	*Psychrobacter frigidicola*	https://doi.org/10.1128/jb.01137-08
GCA_001411745.2	Psychrophiles		3490652	Bacteria	Proteobacteria	Gammaproteobacteria	Pseudomonadales	Moraxellaceae	Psychrobacter	*Psychrobacter glacincola_A*	56810	*Psychrobacter glacincola*	https://doi.org/10.1099/ijs.0.02457-0
GCA_904846215.1	Psychrophiles		3247549	Bacteria	Proteobacteria	Gammaproteobacteria	Pseudomonadales	Moraxellaceae	Psychrobacter	*Psychrobacter glacincola_B*	56810	*Psychrobacter glacincola*	https://doi.org/10.1099/ijs.0.02457-0
GCA_904846075.1	Psychrophiles		3304715	Bacteria	Proteobacteria	Gammaproteobacteria	Pseudomonadales	Moraxellaceae	Psychrobacter	*Psychrobacter glacincola_C*	56810	*Psychrobacter glacincola*	https://doi.org/10.1099/ijs.0.02457-0
GCA_003148585.1	Psychrophiles		3243066	Bacteria	Proteobacteria	Gammaproteobacteria	Pseudomonadales	Moraxellaceae	Psychrobacter	*Psychrobacter immobilis*	498	*Psychrobacter immobilis*	https://bacdive.dsmz.de/strain/8176
GCA_904846265.1	Psychrophiles		3444614	Bacteria	Proteobacteria	Gammaproteobacteria	Pseudomonadales	Moraxellaceae	Psychrobacter	*Psychrobacter immobilis_A*	498	*Psychrobacter immobilis*	https://bacdive.dsmz.de/strain/8176
GCA_904846185.1	Psychrophiles		3473556	Bacteria	Proteobacteria	Gammaproteobacteria	Pseudomonadales	Moraxellaceae	Psychrobacter	*Psychrobacter immobilis_B*	498	*Psychrobacter immobilis*	https://bacdive.dsmz.de/strain/8176
GCA_904846235.1	Psychrophiles		3223472	Bacteria	Proteobacteria	Gammaproteobacteria	Pseudomonadales	Moraxellaceae	Psychrobacter	*Psychrobacter immobilis_C*	498	*Psychrobacter immobilis*	https://bacdive.dsmz.de/strain/8176
GCA_904846285.1	Psychrophiles		3229758	Bacteria	Proteobacteria	Gammaproteobacteria	Pseudomonadales	Moraxellaceae	Psychrobacter	*Psychrobacter immobilis_D*	498	*Psychrobacter immobilis*	https://bacdive.dsmz.de/strain/8176
GCA_904846175.1	Psychrophiles		3123162	Bacteria	Proteobacteria	Gammaproteobacteria	Pseudomonadales	Moraxellaceae	Psychrobacter	*Psychrobacter immobilis_E*	498	*Psychrobacter immobilis*	https://bacdive.dsmz.de/strain/8176
GCA_904846225.1	Psychrophiles		3336711	Bacteria	Proteobacteria	Gammaproteobacteria	Pseudomonadales	Moraxellaceae	Psychrobacter	*Psychrobacter immobilis_F*	498	*Psychrobacter immobilis*	https://bacdive.dsmz.de/strain/8176
GCA_904846145.1	Psychrophiles		3247678	Bacteria	Proteobacteria	Gammaproteobacteria	Pseudomonadales	Moraxellaceae	Psychrobacter	*Psychrobacter immobilis_G*	498	*Psychrobacter immobilis*	https://bacdive.dsmz.de/strain/8176
GCA_000382145.1	Psychrophiles		3176011	Bacteria	Proteobacteria	Gammaproteobacteria	Pseudomonadales	Moraxellaceae	Psychrobacter	*Psychrobacter lutiphocae*	1123033	*Psychrobacter lutiphocae DSM 21542*	https://doi.org/10.1099/ijs.0.008706-0
GCA_900101915.1	Psychrophiles		3062581	Bacteria	Proteobacteria	Gammaproteobacteria	Pseudomonadales	Moraxellaceae	Psychrobacter	*Psychrobacter pacificensis*	112002	*Psychrobacter pacificensis*	https://pubmed.ncbi.nlm.nih.gov/23868329/
GCA_900162825.1	Psychrophiles		2820896	Bacteria	Proteobacteria	Gammaproteobacteria	Pseudomonadales	Moraxellaceae	Psychrobacter	*Psychrobacter piechaudii*	1945521	*Psychrobacter piechaudii*	https://www.microbiologyresearch.org/content/journal/ijsem/10.1099/ijsem.0.002065
GCA_001444505.1	Psychrophiles		3089314	Bacteria	Proteobacteria	Gammaproteobacteria	Pseudomonadales	Moraxellaceae	Psychrobacter	*Psychrobacter piscatorii*	554343	*Psychrobacter piscatorii*	https://doi.org/10.1099/ijs.0.010959-0
GCA_904846415.1	Psychrophiles		3514701	Bacteria	Proteobacteria	Gammaproteobacteria	Pseudomonadales	Moraxellaceae	Psychrobacter	*Psychrobacter piscatorii_A*	554343	*Psychrobacter piscatorii*	https://doi.org/10.1099/ijs.0.010959-0
GCA_003350005.1	Psychrophiles		3031855	Bacteria	Proteobacteria	Gammaproteobacteria	Pseudomonadales	Moraxellaceae	Psychrobacter	*Psychrobacter proteolyticus_A*	147825	*Psychrobacter proteolyticus*	https://doi.org/10.1078/0723-2020-00006
GCA_001298525.1	Psychrophiles		2931580	Bacteria	Proteobacteria	Gammaproteobacteria	Pseudomonadales	Moraxellaceae	Psychrobacter	*Psychrobacter urativorans_A*	45610	*Psychrobacter urativorans*	https://bacdive.dsmz.de/strain/8178
GCA_904846675.1	Psychrophiles		2734405	Bacteria	Proteobacteria	Gammaproteobacteria	Pseudomonadales	Moraxellaceae	Psychrobacter	*Psychrobacter urativorans_B*	45610	*Psychrobacter urativorans*	https://bacdive.dsmz.de/strain/8178
GCA_904846695.1	Psychrophiles		3458123	Bacteria	Proteobacteria	Gammaproteobacteria	Pseudomonadales	Moraxellaceae	Psychrobacter	*Psychrobacter urativorans_C*	45610	*Psychrobacter urativorans*	https://bacdive.dsmz.de/strain/8178
GCA_000153485.2	Psychrophiles		4321832	Bacteria	Bacteroidota	Bacteroidia	Flavobacteriales	Flavobacteriaceae	Psychroflexus	*Psychroflexus torquis*	313595	*Psychroflexus torquis ATCC 700755*	https://doi.org/10.1099/00221287-144-6-1601
GCA_000428725.1	Psychrophiles		5534630	Bacteria	Proteobacteria	Gammaproteobacteria	Enterobacterales	Psychromonadaceae	Psychromonas	*Psychromonas aquimarina*	1278312	*Psychromonas aquimarina ATCC BAA-1526*	https://doi.org/10.1099/ijs.0.65744-0
GCA_000482725.1	Psychrophiles		4745897	Bacteria	Proteobacteria	Gammaproteobacteria	Enterobacterales	Psychromonadaceae	Psychromonas	*Psychromonas arctica*	1123036	*Psychromonas arctica DSM 14288*	https://doi.org/10.1099/ijs.0.02182-0
GCA_000420245.1	Psychrophiles		3979980	Bacteria	Proteobacteria	Gammaproteobacteria	Enterobacterales	Psychromonadaceae	Psychromonas	*Psychromonas hadalis*	1278302	*Psychromonas hadalis ATCC BAA-638*	https://doi.org/10.1099/ijs.0.64933-0
GCA_000015285.1	Psychrophiles		4559598	Bacteria	Proteobacteria	Gammaproteobacteria	Enterobacterales	Psychromonadaceae	Psychromonas	*Psychromonas ingrahamii*	357804	*Psychromonas ingrahamii 37*	https://doi.org/10.1099/ijs.0.64068-0

GCA_000381745.1	Psychrophiles		5204311	Bacteria	Proteobacteria	Gammaproteobacteria	Enterobacterales	Psychromonadaceae	Psychromonas	*Psychromonas ossibalaenae*	1278307	*Psychromonas ossibalaenae ATCC BAA-1528*	https://doi.org/10.1099/ijs.0.65744-0
GCA_000007225.1	Hyperthermophiles		2222430	Archaea	Thermoproteota	Thermoproteia	Thermoproteales	Thermoproteaceae	Pyrobaculum	*Pyrobaculum aerophilum*	13773	*Pyrobaculum aerophilum*	https://pubmed.ncbi.nlm.nih.gov/19047344/
GCA_000016385.1	Hyperthermophiles		2121076	Archaea	Thermoproteota	Thermoproteia	Thermoproteales	Thermoproteaceae	Pyrobaculum	*Pyrobaculum arsenaticum*	121277	*Pyrobaculum arsenaticum PZ6, DSM 13514*	https://bacdive.dsmz.de/strain/17028
GCA_000015805.1	Hyperthermophiles		2009313	Archaea	Thermoproteota	Thermoproteia	Thermoproteales	Thermoproteaceae	Pyrobaculum	*Pyrobaculum calidifontis*	410359	*Pyrobaculum calidifontis VA1*	https://doi.org/10.1155/2002/616075
GCA_000234805.1	Hyperthermophiles		2467972	Archaea	Thermoproteota	Thermoproteia	Thermoproteales	Thermoproteaceae	Pyrobaculum	*Pyrobaculum ferrireducens*	1104324	*Pyrobaculum ferrireducens*	https://doi.org/10.1099/ijs.0.000027
GCA_000015205.1	Hyperthermophiles		1826402	Archaea	Thermoproteota	Thermoproteia	Thermoproteales	Thermoproteaceae	Pyrobaculum	*Pyrobaculum islandicum*	384616	*Pyrobaculum islandicum DSM 4184*	https://doi.org/10.1271/bbb.120367
GCA_000019805.1	Hyperthermophiles		1769823	Archaea	Thermoproteota	Thermoproteia	Thermoproteales	Thermoproteaceae	Pyrobaculum	*Pyrobaculum neutrophilum*	444157	*Thermoproteus neutrophilus*	https://bacdive.dsmz.de/strain/17030
GCA_000247545.1	Hyperthermophiles		2452920	Archaea	Thermoproteota	Thermoproteia	Thermoproteales	Thermoproteaceae	Pyrobaculum	*Pyrobaculum oguniense*	698757	*Pyrobaculum oguniense TE7, DSM 13380*	https://doi.org/10.1099/00207713-51-2-303
GCA_001189275.1	Thermophiles		1993257	Archaea	Thermoproteota	Thermoproteia	Thermoproteales	Thermoproteaceae	Pyrobaculum	*Pyrobaculum sp001189275*	1227555	*Pyrobaculum yellowstonensis sp WP30*	https://doi.org/10.1128/aem.01095-15
GCA_000195935.2	Hyperthermophiles		1768562	Archaea	Methanobacteriota_B	Thermococci	Thermococcales	Thermococcaceae	Pyrococcus	*Pyrococcus abyssi*	272844	*Pyrococcus abyssi GE5*	https://bacdive.dsmz.de/strain/16859
GCA_002214605.1	Hyperthermophiles		1961979	Archaea	Methanobacteriota_B	Thermococci	Thermococcales	Thermococcaceae	Pyrococcus	*Pyrococcus chitonophagus*	54262	*Pyrococcus chitonophagus DSM 10152*	https://doi.org/10.1128/aem.00319-16
GCA_000007305.1	Hyperthermophiles		1908256	Archaea	Methanobacteriota_B	Thermococci	Thermococcales	Thermococcaceae	Pyrococcus	*Pyrococcus furiosus*	186497	*Pyrococcus furiosus*	https://bacdive.dsmz.de/strain/16854
GCA_000211475.1	Hyperthermophiles		1861320	Archaea	Methanobacteriota_B	Thermococci	Thermococcales	Thermococcaceae	Pyrococcus	*Pyrococcus sp000211475*	342949	*Pyrococcus sp NA2*	https://doi.org/10.1128/jb.05150-11
GCA_000215995.1	Hyperthermophiles	Acidophiles	1716818	Archaea	Methanobacteriota_B	Thermococci	Thermococcales	Thermococcaceae	Pyrococcus	*Pyrococcus yayanosii*	529709	*Pyrococcus yayanosii CH1*	https://bacdive.dsmz.de/strain/161812
GCA_001412615.1	Hyperthermophiles		2023836	Archaea	Thermoproteota	Thermoproteia	Sulfolobales	Pyrodictiaceae	Pyrodictium	*Pyrodictium delaneyi*	1273541	*Pyrodictium delaneyi*	https://www.microbiologyresearch.org/content/journal/ijsem/10.1099/ijsem.0.001201
GCA_001462395.1	Hyperthermophiles		1621727	Archaea	Thermoproteota	Thermoproteia	Sulfolobales	Pyrodictiaceae	Pyrodictium	*Pyrodictium occultum*	2309	*Pyrodictium occultum*	https://doi.org/10.1128/jb.177.8.2164-2177.1995
GCA_000223395.1	Hyperthermophiles	Acidophiles	1843267	Archaea	Thermoproteota	Thermoproteia	Sulfolobales	Pyrodictiaceae	Pyrolobus	*Pyrolobus fumarii*	694429	*Pyrolobus fumarii 1A*	https://doi.org/10.1007/s007920050010
GCA_009765975.1		Acidophiles	8370829	Bacteria	Proteobacteria	Alphaproteobacteria	Acetobacterales	Acetobacteraceae	Rhodopila	*Rhodopila sp009765975*	1747223	*Acidisphaera sp. S103*	https://doi.org/10.1099/00207713-50-4-1539
GCA_000024845.1	Thermophiles		3386737	Bacteria	Bacteroidota	Rhodothermia	Rhodothermales	Rhodothermaceae	Rhodothermus	*Rhodothermus marinus*	518766	*Rhodothermus marinus DSM 4252*	https://bacdive.dsmz.de/strain/17794
GCA_900142415.1	Thermophiles		3139689	Bacteria	Bacteroidota	Rhodothermia	Rhodothermales	Rhodothermaceae	Rhodothermus	*Rhodothermus profundi*	633813	*Rhodothermus profundi*	https://doi.org/10.1099/ijs.0.012724-0
GCA_003568865.1	Mesophiles		3078689	Bacteria	Actinobacteriota	Rubrobacteria	Rubrobacterales	Rubrobacteraceae	Rubrobacter	*Rubrobacter indicoceani*	2051957	*Rubrobacter indicoceani SCSIO 08198*	https://www.microbiologyresearch.org/content/journal/ijsem/10.1099/ijsem.0.003018
GCA_900175965.1		Alkaliphiles	3398074	Bacteria	Actinobacteriota	Rubrobacteria	Rubrobacterales	Rubrobacteraceae	Rubrobacter	*Rubrobacter radiotolerans*	42256	*Rubrobacter radiotolerans*	https://www.ncbi.nlm.nih.gov/pmc/articles/PMC4148983/
GCA_000014185.1	Thermophiles	Alkaliphiles	3225748	Bacteria	Actinobacteriota	Rubrobacteria	Rubrobacterales	Rubrobacteraceae	Rubrobacter_B	*Rubrobacter_B xylanophilus*	266117	*Rubrobacter xylanophilus DSM 9941*	https://bacdive.dsmz.de/strain/14036
GCA_000632715.1	Thermophiles		3798752	Bacteria	Firmicutes	Bacilli	Bacillales	Anoxybacillaceae	Saccharococcus	*Saccharococcus caldoxylosilyticus*	81408	*Parageobacillus caldoxylosilyticus*	https://doi.org/10.1016/j.dib.2021.107764
GCA_000022485.1	Hyperthermophiles	Acidophiles	2854410	Archaea	Thermoproteota	Thermoproteia	Sulfolobales	Sulfolobaceae	Saccharolobus	*Saccharolobus islandicus*	425944	*Sulfolobus islandicus L.D.8.5*	https://doi.org/10.1007/978-1-0716-2445-6_10
GCA_900079115.1	Hyperthermophiles	Acidophiles	3034024	Archaea	Thermoproteota	Thermoproteia	Sulfolobales	Sulfolobaceae	Saccharolobus	*Saccharolobus solfataricus*	2287	*Saccharolobus solfataricus*	https://doi.org/10.1099/ijsem.0.002665
GCA_001719125.1	Thermophiles	Acidophiles	2688317	Archaea	Thermoproteota	Thermoproteia	Sulfolobales	Sulfolobaceae	Saccharolobus	*Saccharolobus sp001719125*	1891280	*Sulfolobus sp. A20*	https://www.frontiersin.org/articles/10.3389/fmicb.2016.01902/full
GCA_007004735.1	Thermophiles		4196798	Archaea	Halobacteriota	Halobacteria	Halobacteriales	Haloferacaceae	Salinigranum	*Salinigranum halophilum*	2565931	*Salinigranum halophilum*	https://www.microbiologyresearch.org/content/journal/ijsem/10.1099/ijsem.0.003951
GCA_003226325.1		Alkaliphiles	4150426	Bacteria	Firmicutes	Bacilli	Bacillales_H	Salisediminibacteriaceae	Salipaludibacillus	*Salipaludibacillus keqinensis*	2045207	*Salipaludibacillus keqinensis*	https://pubmed.ncbi.nlm.nih.gov/30788630/
GCA_009183365.2	Mesophiles		4924764	Bacteria	Proteobacteria	Gammaproteobacteria	Enterobacterales	Shewanellaceae	Shewanella	*Shewanella algae*	38313	*Shewanella algae*	https://bacdive.dsmz.de/strain/14062
GCA_003605125.1	Psychrophiles		4203325	Bacteria	Proteobacteria	Gammaproteobacteria	Enterobacterales	Shewanellaceae	Shewanella	*Shewanella algidipiscicola*	614070	*Shewanella algidipiscicola*	https://doi.org/10.1099/ijs.0.64708-0
GCA_003966265.1	Psychrophiles		5392127	Bacteria	Proteobacteria	Gammaproteobacteria	Enterobacterales	Shewanellaceae	Shewanella	*Shewanella atlantica*	271099	*Shewanella atlantica*	https://doi.org/10.1099/ijs.0.64708-0
GCA_900456975.1	Psychrophiles		5300842	Bacteria	Proteobacteria	Gammaproteobacteria	Enterobacterales	Shewanellaceae	Shewanella	*Shewanella baltica*	693974	*Shewanella baltica BA175*	https://doi.org/10.1016/j.jprot.2019.103419
GCA_003966225.1	Psychrophiles		5676915	Bacteria	Proteobacteria	Gammaproteobacteria	Enterobacterales	Shewanellaceae	Shewanella	*Shewanella canadensis*	271096	*Shewanella canadensis*	https://doi.org/10.1099/ijs.0.64596-0
GCA_002777975.1	Mesophiles		4375287	Bacteria	Proteobacteria	Gammaproteobacteria	Enterobacterales	Shewanellaceae	Shewanella	*Shewanella carassii*	1987584	*Shewanella carassii*	https://bacdive.dsmz.de/strain/158312
GCA_002836945.1		Alkaliphiles	4399136	Bacteria	Proteobacteria	Gammaproteobacteria	Enterobacterales	Shewanellaceae	Shewanella	*Shewanella chilikensis*	558541	*Shewanella chilikensis*	https://doi.org/10.1099/ijs.0.010918-0
GCA_000518705.1	Mesophiles		4575622	Bacteria	Proteobacteria	Gammaproteobacteria	Enterobacterales	Shewanellaceae	Shewanella	*Shewanella colwelliana*	1336240	*Shewanella colwelliana ATCC 39565*	https://bacdive.dsmz.de/strain/14082
GCA_003353085.1	Mesophiles		5215037	Bacteria	Proteobacteria	Gammaproteobacteria	Enterobacterales	Shewanellaceae	Shewanella	*Shewanella corallii*	560080	*Shewanella corallii*	https://doi.org/10.1099/ijs.0.015768-0
GCA_000013765.1	Mesophiles		4545906	Bacteria	Proteobacteria	Gammaproteobacteria	Enterobacterales	Shewanellaceae	Shewanella	*Shewanella denitrificans*	318161	*Shewanella denitrificans OS217*	https://www.microbiologyresearch.org/docserver/fulltext/ijsem/52/6/0522211a.pdf?expires=1677115145&id=id&accname=guest&checksum=9A4347E7FD89240A16EF15F868F657D3
GCA_007567505.1	Psychrophiles		4912773	Bacteria	Proteobacteria	Gammaproteobacteria	Enterobacterales	Shewanellaceae	Shewanella	*Shewanella donghaensis*	238836	*Shewanella donghaensis*	https://doi.org/10.1099/ijs.0.64469-0
GCA_000518605.1	Mesophiles		4798688	Bacteria	Proteobacteria	Gammaproteobacteria	Enterobacterales	Shewanellaceae	Shewanella	*Shewanella fidelis*	1336247	*Shewanella fidelis ATCC BAA-318*	https://www.microbiologyresearch.org/content/journal/ijsem/10.1099/ijs.0.02198-0
GCA_014651955.1	Mesophiles		3905718	Bacteria	Proteobacteria	Gammaproteobacteria	Enterobacterales	Shewanellaceae	Shewanella	*Shewanella fodinae*	552357	*Shewanella fodinae*	https://doi.org/10.1099/ijs.0.017046-0
GCA_003797125.1	Psychrophiles		4784071	Bacteria	Proteobacteria	Gammaproteobacteria	Enterobacterales	Shewanellaceae	Shewanella	*Shewanella frigidimarina*	56812	*Shewanella frigidimarina*	https://www.microbiologyresearch.org/content/journal/ijsem/10.1099/00207713-52-1-195
GCA_000019185.1	Psychrophiles		5226917	Bacteria	Proteobacteria	Gammaproteobacteria	Enterobacterales	Shewanellaceae	Shewanella	*Shewanella halifaxensis*	271098	*Shewanella halifaxensis*	https://doi.org/10.1099/ijs.0.63829-0
GCA_007197645.1	Psychrophiles		5939027	Bacteria	Proteobacteria	Gammaproteobacteria	Enterobacterales	Shewanellaceae	Shewanella	*Shewanella hanedai*	25	*Shewanella hanedai*	https://www.microbiologyresearch.org/content/journal/ijsem/10.1099/ijsem.0.005152
GCA_002836975.1		Alkaliphiles	4402806	Bacteria	Proteobacteria	Gammaproteobacteria	Enterobacterales	Shewanellaceae	Shewanella	*Shewanella indica*	768528	*Shewanella indica*	https://pubmed.ncbi.nlm.nih.gov/20851908/
GCA_002075795.1	Mesophiles		4975677	Bacteria	Proteobacteria	Gammaproteobacteria	Enterobacterales	Shewanellaceae	Shewanella	*Shewanella japonica*	93973	*Shewanella japonica*	https://www.microbiologyresearch.org/content/journal/ijsem/10.1099/00207713-51-3-1027
GCA_003855395.1	Psychrophiles		4839879	Bacteria	Proteobacteria	Gammaproteobacteria	Enterobacterales	Shewanellaceae	Shewanella	*Shewanella livingstonensis*	150120	*Shewanella livingstonensis*	https://doi.org/10.1099/00207713-52-1-195
GCA_000016065.1	Psychrophiles		4602594	Bacteria	Proteobacteria	Gammaproteobacteria	Enterobacterales	Shewanellaceae	Shewanella	*Shewanella loihica*	323850	*Shewanella loihica PV-4*	https://doi.org/10.1099/ijs.0.64354-0
GCA_000753795.1	Psychrophiles		4215794	Bacteria	Proteobacteria	Gammaproteobacteria	Enterobacterales	Shewanellaceae	Shewanella	*Shewanella mangrovi*	1515746	*Shewanella mangrovi*	https://doi.org/10.1099/ijs.0.000313
GCA_000614975.1	Psychrophiles		4424648	Bacteria	Proteobacteria	Gammaproteobacteria	Enterobacterales	Shewanellaceae	Shewanella	*Shewanella marina*	1236542	*Shewanella marina JCM 15074*	https://www.microbiologyresearch.org/content/journal/ijsem/10.1099/ijs.0.005470-0
GCA_002215585.1	Psychrophiles		4321452	Bacteria	Proteobacteria	Gammaproteobacteria	Enterobacterales	Shewanellaceae	Shewanella	*Shewanella marisflavi*	260364	*Shewanella marisflavi*	https://doi.org/10.1099/ijs.0.63198-0
GCA_900156405.1	Psychrophiles		4190369	Bacteria	Proteobacteria	Gammaproteobacteria	Enterobacterales	Shewanellaceae	Shewanella	*Shewanella morhuae*	365591	*Shewanella morhuae*	https://www.microbiologyresearch.org/content/journal/ijsem/10.1099/ijs.0.63931-0
GCA_000018285.1	Mesophiles		5174581	Bacteria	Proteobacteria	Gammaproteobacteria	Enterobacterales	Shewanellaceae	Shewanella	*Shewanella pealeana*	398579	*Shewanella pealeana ATCC 700345*	https://doi.org/10.1099/00207713-49-4-1341
GCA_000014885.1	Psychrophiles		5396476	Bacteria	Proteobacteria	Gammaproteobacteria	Enterobacterales	Shewanellaceae	Shewanella	*Shewanella piezotolerans*	225849	*Shewanella piezotolerans WP3*	https://doi.org/10.1099/ijs.0.64500-0
GCA_002005305.1	Psychrophiles		6353406	Bacteria	Proteobacteria	Gammaproteobacteria	Enterobacterales	Shewanellaceae	Shewanella	*Shewanella psychrophila*	225848	*Shewanella psychrophila*	https://doi.org/10.1099/ijs.0.64500-0
GCA_016406325.1	Mesophiles		4386330	Bacteria	Proteobacteria	Gammaproteobacteria	Enterobacterales	Shewanellaceae	Shewanella	*Shewanella putrefaciens*	24	*Shewanella putrefaciens*	https://bacdive.dsmz.de/strain/14054
GCA_016406305.1	Mesophiles		4575397	Bacteria	Proteobacteria	Gammaproteobacteria	Enterobacterales	Shewanellaceae	Shewanella	*Shewanella putrefaciens_C*	24	*Shewanella putrefaciens*	https://bacdive.dsmz.de/strain/14054
GCA_000018025.1	Psychrophiles		5517674	Bacteria	Proteobacteria	Gammaproteobacteria	Enterobacterales	Shewanellaceae	Shewanella	*Shewanella sediminis*	425104	*Shewanella sediminis HAW-EB3*	https://doi.org/10.1099/ijs.0.63604-0
GCA_003797885.1	Psychrophiles		4722637	Bacteria	Proteobacteria	Gammaproteobacteria	Enterobacterales	Shewanellaceae	Shewanella	*Shewanella vesiculosa*	518738	*Shewanella vesiculosa*	https://doi.org/10.1099/ijs.0.000737-0
GCA_000091325.1	Psychrophiles		4962103	Bacteria	Proteobacteria	Gammaproteobacteria	Enterobacterales	Shewanellaceae	Shewanella	*Shewanella violacea*	637905	*Shewanella violacea DSS12*	https://doi.org/10.1039/c000396d

GCA_000518805.1	Mesophiles		4971480	Bacteria	Proteobacteria	Gammaproteobacteria	Enterobacterales	Shewanellaceae	Shewanella	*Shewanella waksmanii*	1336233	*Shewanella waksmanii ATCC BAA-643*	https://pubmed.ncbi.nlm.nih.gov/13130035/
GCA_000019525.1	Psychrophiles		5935403	Bacteria	Proteobacteria	Gammaproteobacteria	Enterobacterales	Shewanellaceae	Shewanella	*Shewanella woodyi*	392500	*Shewanella woodyi ATCC 51908*	https://www.microbiologyresearch.org/content/journal/ijsem/10.1099/00207713-47-4-1034
GCA_014647135.1	Mesophiles		4600673	Bacteria	Proteobacteria	Gammaproteobacteria	Enterobacterales	Shewanellaceae	Shewanella	*Shewanella xiamenensis*	332186	*Shewanella xiamenensis*	https://bacdive.dsmz.de/strain/14084
GCA_004123295.1		Acidophiles	5116187	Bacteria	Acidobacteriota	Acidobacteriae	Acidobacteriales	Acidobacteriaceae	Silvibacterium	*Silvibacterium dinghuense*	1560006	*Silvibacterium dinghuense*	https://doi.org/10.1099/ijsem.0.005415
GCA_900105965.1	Psychrophiles		4622641	Bacteria	Actinobacteriota	Actinomycetia	Actinomycetales	Micrococcaceae	Specibacter	*Specibacter alpinus*	656366	*Arthrobacter alpinus*	https://doi.org/10.1099/ijs.0.017178-0
GCA_001294625.1	Psychrophiles		4046453	Bacteria	Actinobacteriota	Actinomycetia	Actinomycetales	Micrococcaceae	Specibacter	*Specibacter alpinus_A*	656366	*Arthrobacter alpinus*	https://doi.org/10.1099/ijs.0.017178-0
GCA_001445575.1	Psychrophiles		4333648	Bacteria	Actinobacteriota	Actinomycetia	Actinomycetales	Micrococcaceae	Specibacter	*Specibacter alpinus_C*	656366	*Arthrobacter alpinus*	https://doi.org/10.1099/ijs.0.017178-0
GCA_002909445.1	Psychrophiles		4469153	Bacteria	Actinobacteriota	Actinomycetia	Actinomycetales	Micrococcaceae	Specibacter	*Specibacter glacialis*	1664	*Arthrobacter glacialis*	https://doi.org/10.1016/j.syapm.2018.10.005
GCA_003219815.1	Psychrophiles		5042614	Bacteria	Actinobacteriota	Actinomycetia	Actinomycetales	Micrococcaceae	Specibacter	*Specibacter livingstonensis*	670078	*Arthrobacter livingstonensis*	https://doi.org/10.1099/ijs.0.021022-0
GCA_003185915.1	Psychrophiles		4049680	Bacteria	Actinobacteriota	Actinomycetia	Actinomycetales	Micrococcaceae	Specibacter	*Specibacter psychrochitiniphilus*	291045	*Arthrobacter psychrochitiniphilus*	https://doi.org/10.1099/ijs.0.008912-0
GCA_003219795.1	Psychrophiles		3871146	Bacteria	Actinobacteriota	Actinomycetia	Actinomycetales	Micrococcaceae	Specibacter	*Specibacter psychrolactophilus*	92442	*Arthrobacter psychrolactophilus*	https://doi.org/10.1007/s002030050722
GCA_000242595.3		Alkaliphiles	3285855	Bacteria	Spirochaetota	Spirochaetia	DSM-27196	DSM-8902	Spirochaeta_B	*Spirochaeta_B africana*	889378	*Spirochaeta africana DSM 8902*	https://doi.org/10.1099/00207713-46-1-305
GCA_002901865.1	Mesophiles		2491359	Bacteria	Firmicutes	Bacilli	Staphylococcales	Staphylococcaceae	Staphylococcus	*Staphylococcus agnetis*	985762	*Staphylococcus agnetis*	https://bacdive.dsmz.de/strain/14677
GCA_002902305.1	Mesophiles		2452468	Bacteria	Firmicutes	Bacilli	Staphylococcales	Staphylococcaceae	Staphylococcus	*Staphylococcus argensis*	1607738	*Staphylococcus argensis*	https://bacdive.dsmz.de/strain/132229
GCA_000236925.1	Mesophiles		2787638	Bacteria	Firmicutes	Bacilli	Staphylococcales	Staphylococcaceae	Staphylococcus	*Staphylococcus argenteus*	985002	*Staphylococcus argenteus*	https://bacdive.dsmz.de/strain/130706
GCA_002902345.1	Mesophiles		2665344	Bacteria	Firmicutes	Bacilli	Staphylococcales	Staphylococcaceae	Staphylococcus	*Staphylococcus arlettae*	29378	*Staphylococcus arlettae*	https://bacdive.dsmz.de/strain/14441
GCA_001027105.1	Mesophiles		2782562	Bacteria	Firmicutes	Bacilli	Staphylococcales	Staphylococcaceae	Staphylococcus	*Staphylococcus aureus*	1280	*Staphylococcus aureus*	https://bacdive.dsmz.de/strain/14487
GCA_001500315.1	Mesophiles		2201708	Bacteria	Firmicutes	Bacilli	Staphylococcales	Staphylococcaceae	Staphylococcus	*Staphylococcus auricularis*	29379	*Staphylococcus auricularis*	https://bacdive.dsmz.de/strain/14503
GCA_002902325.1	Mesophiles		2434909	Bacteria	Firmicutes	Bacilli	Staphylococcales	Staphylococcaceae	Staphylococcus	*Staphylococcus capitis*	904334	*Staphylococcus capitis VCU116*	https://bacdive.dsmz.de/strain/14508
GCA_002902725.1	Mesophiles		2606761	Bacteria	Firmicutes	Bacilli	Staphylococcales	Staphylococcaceae	Staphylococcus	*Staphylococcus caprae*	29380	*Staphylococcus caprae*	https://bacdive.dsmz.de/strain/14509
GCA_900458435.1	Mesophiles		2576833	Bacteria	Firmicutes	Bacilli	Staphylococcales	Staphylococcaceae	Staphylococcus	*Staphylococcus carnosus*	1281	*Staphylococcus carnosus*	https://bacdive.dsmz.de/strain/14516
GCA_002901945.1	Mesophiles		2276768	Bacteria	Firmicutes	Bacilli	Staphylococcales	Staphylococcaceae	Staphylococcus	*Staphylococcus chromogenes*	46126	*Staphylococcus chromogenes*	https://bacdive.dsmz.de/strain/14517
GCA_900636325.1	Mesophiles		2798161	Bacteria	Firmicutes	Bacilli	Staphylococcales	Staphylococcaceae	Staphylococcus	*Staphylococcus delphini*	53344	*Staphylococcus delphini*	https://bacdive.dsmz.de/strain/14521
GCA_002902625.1	Mesophiles		2379883	Bacteria	Firmicutes	Bacilli	Staphylococcales	Staphylococcaceae	Staphylococcus	*Staphylococcus devriesei*	586733	*Staphylococcus devriesei*	https://www.microbiologyresearch.org/content/journal/ijsem/10.1099/ijs.0.015982-0
GCA_003035445.1	Mesophiles		2394070	Bacteria	Firmicutes	Bacilli	Staphylococcales	Staphylococcaceae	Staphylococcus	*Staphylococcus devriesei_A*	586733	*Staphylococcus devriesei*	https://www.microbiologyresearch.org/content/journal/ijsem/10.1099/ijs.0.015982-0
GCA_900458565.1	Mesophiles		2758007	Bacteria	Firmicutes	Bacilli	Staphylococcales	Staphylococcaceae	Staphylococcus	*Staphylococcus equorum*	1357294	*Staphylococcus equorum UMC-CNS-924*	https://bacdive.dsmz.de/strain/14662
GCA_003012915.1	Mesophiles		2479423	Bacteria	Firmicutes	Bacilli	Staphylococcales	Staphylococcaceae	Staphylococcus	*Staphylococcus felis*	46127	*Staphylococcus felis*	https://bacdive.dsmz.de/strain/14607
GCA_000875895.1	Mesophiles		3171720	Bacteria	Firmicutes	Bacilli	Staphylococcales	Staphylococcaceae	Staphylococcus	*Staphylococcus gallinarum*	1293	*Staphylococcus gallinarum*	https://bacdive.dsmz.de/strain/14542
GCA_006094395.1	Mesophiles		2572027	Bacteria	Firmicutes	Bacilli	Staphylococcales	Staphylococcaceae	Staphylococcus	*Staphylococcus haemolyticus*	1283	*Staphylococcus haemolyticus*	https://bacdive.dsmz.de/strain/14544
GCA_002901845.1	Mesophiles		2204528	Bacteria	Firmicutes	Bacilli	Staphylococcales	Staphylococcaceae	Staphylococcus	*Staphylococcus hominis*	1290	*Staphylococcus hominis*	https://bacdive.dsmz.de/strain/14639
GCA_000816085.1	Mesophiles		2472129	Bacteria	Firmicutes	Bacilli	Staphylococcales	Staphylococcaceae	Staphylococcus	*Staphylococcus hyicus*	1284	*Staphylococcus hyicus*	https://bacdive.dsmz.de/strain/14553
GCA_002902385.1	Mesophiles		2801199	Bacteria	Firmicutes	Bacilli	Staphylococcales	Staphylococcaceae	Staphylococcus	*Staphylococcus intermedius*	1285	*Staphylococcus intermedius*	https://bacdive.dsmz.de/strain/14555
GCA_003019255.1	Mesophiles		2639038	Bacteria	Firmicutes	Bacilli	Staphylococcales	Staphylococcaceae	Staphylococcus	*Staphylococcus kloosii*	29384	*Staphylococcus kloosii*	https://bacdive.dsmz.de/strain/14556
GCA_002901705.1	Mesophiles		2519514	Bacteria	Firmicutes	Bacilli	Staphylococcales	Staphylococcaceae	Staphylococcus	*Staphylococcus lugdunensis*	904354	*Staphylococcus lugdunensis VCU150*	https://bacdive.dsmz.de/strain/14562
GCA_000298075.1	Mesophiles		2366595	Bacteria	Firmicutes	Bacilli	Staphylococcales	Staphylococcaceae	Staphylococcus	*Staphylococcus massiliensis*	1229783	*Staphylococcus massiliensis S46*	https://bacdive.dsmz.de/strain/14673
GCA_000934465.1	Mesophiles		2381859	Bacteria	Firmicutes	Bacilli	Staphylococcales	Staphylococcaceae	Staphylococcus	*Staphylococcus microti*	569857	*Staphylococcus microti*	https://bacdive.dsmz.de/strain/14672
GCA_003019275.1	Mesophiles		2095131	Bacteria	Firmicutes	Bacilli	Staphylococcales	Staphylococcaceae	Staphylococcus	*Staphylococcus muscae*	1294	*Staphylococcus muscae*	https://bacdive.dsmz.de/strain/14605
GCA_014635045.1	Mesophiles		2849797	Bacteria	Firmicutes	Bacilli	Staphylococcales	Staphylococcaceae	Staphylococcus	*Staphylococcus nepalensis*	214473	*Staphylococcus nepalensis*	https://bacdive.dsmz.de/strain/14652
GCA_003970495.1	Mesophiles		2462952	Bacteria	Firmicutes	Bacilli	Staphylococcales	Staphylococcaceae	Staphylococcus	*Staphylococcus pasteuri*	45972	*Staphylococcus pasteuri*	https://bacdive.dsmz.de/strain/14617
GCA_002902685.1	Mesophiles		2455272	Bacteria	Firmicutes	Bacilli	Staphylococcales	Staphylococcaceae	Staphylococcus	*Staphylococcus pettenkoferi*	170573	*Staphylococcus pettenkoferi*	https://bacdive.dsmz.de/strain/14671
GCA_900186985.1	Mesophiles		2613271	Bacteria	Firmicutes	Bacilli	Staphylococcales	Staphylococcaceae	Staphylococcus	*Staphylococcus piscifermentans*	70258	*Staphylococcus piscifermentans*	https://bacdive.dsmz.de/strain/14606
GCA_001792775.2	Mesophiles		2519048	Bacteria	Firmicutes	Bacilli	Staphylococcales	Staphylococcaceae	Staphylococcus	*Staphylococcus pseudintermedius*	283734	*Staphylococcus pseudintermedius*	https://bacdive.dsmz.de/strain/14670
GCA_000010125.1	Mesophiles		2577899	Bacteria	Firmicutes	Bacilli	Staphylococcales	Staphylococcaceae	Staphylococcus	*Staphylococcus saprophyticus*	29385	*Staphylococcus saprophyticus*	https://bacdive.dsmz.de/strain/14626
GCA_002902405.1	Mesophiles		2743713	Bacteria	Firmicutes	Bacilli	Staphylococcales	Staphylococcaceae	Staphylococcus	*Staphylococcus schweitzeri*	1654388	*Staphylococcus schweitzeri*	https://bacdive.dsmz.de/strain/130707
GCA_002902285.1	Mesophiles		2735408	Bacteria	Firmicutes	Bacilli	Staphylococcales	Staphylococcaceae	Staphylococcus	*Staphylococcus simulans*	1286	*Staphylococcus simulans*	https://bacdive.dsmz.de/strain/14572
GCA_003043455.1	Mesophiles		2556508	Bacteria	Firmicutes	Bacilli	Staphylococcales	Staphylococcaceae	Staphylococcus	*Staphylococcus simulans_A*	1286	*Staphylococcus simulans*	https://bacdive.dsmz.de/strain/14572
GCA_002994445.1	Mesophiles		2595226	Bacteria	Firmicutes	Bacilli	Staphylococcales	Staphylococcaceae	Staphylococcus	*Staphylococcus simulans_B*	1286	*Staphylococcus simulans*	https://bacdive.dsmz.de/strain/14572
GCA_001006765.1	Mesophiles		2887686	Bacteria	Firmicutes	Bacilli	Staphylococcales	Staphylococcaceae	Staphylococcus	*Staphylococcus succinus*	61015	*Staphylococcus succinus*	https://www.microbiologyresearch.org/content/journal/ijsem/10.1099/00207713-48-2-511
GCA_002902235.1	Mesophiles		2670427	Bacteria	Firmicutes	Bacilli	Staphylococcales	Staphylococcaceae	Staphylococcus	*Staphylococcus ureilyticus*	94138	*Staphylococcus ureilyticus*	https://bacdive.dsmz.de/strain/136260
GCA_900636385.1	Mesophiles		2427576	Bacteria	Firmicutes	Bacilli	Staphylococcales	Staphylococcaceae	Staphylococcus	*Staphylococcus warneri*	904338	*Staphylococcus warneri VCU121*	https://bacdive.dsmz.de/strain/14587
GCA_002732165.1	Mesophiles		2734769	Bacteria	Firmicutes	Bacilli	Staphylococcales	Staphylococcaceae	Staphylococcus	*Staphylococcus xylosus*	1288	*Staphylococcus xylosus*	https://bacdive.dsmz.de/strain/14599
GCA_000338275.1	Mesophiles		2939263	Bacteria	Firmicutes	Bacilli	Staphylococcales	Staphylococcaceae	Staphylococcus	*Staphylococcus xylosus_B*	1288	*Staphylococcus xylosus*	https://bacdive.dsmz.de/strain/14599
GCA_000015945.1	Hyperthermophiles		1570485	Archaea	Thermoproteota	Thermoproteia	Sulfolobales	Desulfurococcaceae	Staphylothermus	*Staphylothermus marinus*	399550	*Staphylothermus marinus F1*	https://www.sciencedirect.com/science/article/pii/S0168165606008595?via%3Dihub#aep-section-id32
GCA_013343115.1	Mesophiles		2358846	Bacteria	Firmicutes	Bacilli	Lactobacillales	Streptococcaceae	Streptococcus	*Streptococcus sanguinis_H*	888825	*Streptococcus sanguinis VMC66*	https://bacdive.dsmz.de/strain/14767
GCA_010604095.1	Mesophiles		2173350	Bacteria	Firmicutes	Bacilli	Lactobacillales	Streptococcaceae	Streptococcus	*Streptococcus sp000187445*	1343	*Streptococcus vestibularis*	https://bacdive.dsmz.de/strain/14794
GCA_900095845.1	Mesophiles		1925331	Bacteria	Firmicutes	Bacilli	Lactobacillales	Streptococcaceae	Streptococcus	*Streptococcus timonensis*	1852387	*Streptococcus timonensis*	https://www.sciencedirect.com/science/article/pii/S2052297516301251?via%3Dihub
GCA_002355215.1	Mesophiles		2097874	Bacteria	Firmicutes	Bacilli	Lactobacillales	Streptococcaceae	Streptococcus	*Streptococcus troglodytae*	1111760	*Streptococcus troglodytae*	https://www.microbiologyresearch.org/content/journal/ijsem/10.1099/ijs.0.039388-0
GCA_900475595.1	Mesophiles		1975601	Bacteria	Firmicutes	Bacilli	Lactobacillales	Streptococcaceae	Streptococcus	*Streptococcus uberis*	1349	*Streptococcus uberis*	https://bacdive.dsmz.de/strain/14787
GCA_000785785.1	Mesophiles		2149440	Bacteria	Firmicutes	Bacilli	Lactobacillales	Streptococcaceae	Streptococcus	*Streptococcus uberis_A*	1349	*Streptococcus uberis*	https://bacdive.dsmz.de/strain/14787
GCA_000188055.3	Mesophiles		2130431	Bacteria	Firmicutes	Bacilli	Lactobacillales	Streptococcaceae	Streptococcus	*Streptococcus urinalis*	764291	*Streptococcus urinalis 2285-97*	https://bacdive.dsmz.de/strain/14822
GCA_001375655.1	Mesophiles		2460376	Bacteria	Firmicutes	Bacilli	Lactobacillales	Streptococcaceae	Streptococcus	*Streptococcus varani*	1608583	*Streptococcus varani*	https://bacdive.dsmz.de/strain/139780
GCA_000188295.1	Mesophiles		1872773	Bacteria	Firmicutes	Bacilli	Lactobacillales	Streptococcaceae	Streptococcus	*Streptococcus vestibularis*	1343	*Streptococcus vestibularis*	https://bacdive.dsmz.de/strain/14794
GCA_009729035.1	Thermophiles	Acidophiles	1987069	Archaea	Thermoproteota	Thermoproteia	Sulfolobales	Sulfolobaceae	Stygiolobus	*Stygiolobus azoricus*	1313299	*Stygiolobus azoricus*	https://doi.org/10.1128/mra.01490-19
GCA_006716115.1	Psychrophiles		4214389	Bacteria	Actinobacteriota	Actinomycetia	Actinomycetales	Microbacteriaceae	Subtercola	*Subtercola boreus*	120213	*Subtercola boreus*	https://doi.org/10.1099/00207713-50-5-1731
GCA_003399645.1	Psychrophiles		3670876	Bacteria	Actinobacteriota	Actinomycetia	Actinomycetales	Microbacteriaceae	Subtercola	*Subtercola boreus_A*	120213	*Subtercola boreus*	https://doi.org/10.1099/00207713-50-5-1731
GCA_003399635.1	Psychrophiles		3848592	Bacteria	Actinobacteriota	Actinomycetia	Actinomycetales	Microbacteriaceae	Subtercola	*Subtercola boreus_B*	120213	*Subtercola boreus*	https://doi.org/10.1099/00207713-50-5-1731
GCA_003399625.1	Psychrophiles		4121892	Bacteria	Actinobacteriota	Actinomycetia	Actinomycetales	Microbacteriaceae	Subtercola	*Subtercola boreus_C*	120213	*Subtercola boreus*	https://doi.org/10.1099/00207713-50-5-1731

GCA_004923255.1	Psychrophiles		4043135	Bacteria	Actinobacteriota	Actinomycetia	Actinomycetales	Microbacteriaceae	Subtercola	*Subtercola vilae*	2056433	*Subtercola vilae*	https://pubmed.ncbi.nlm.nih.gov/29214367/
GCA_003023725.1		Acidophiles	4556669	Bacteria	Firmicutes_E	Sulfobacillia	Sulfobacillales	Sulfobacillaceae	Sulfobacillus	*Sulfobacillus benefaciens_A*	453960	*Sulfobacillus benefaciens*	https://doi.org/10.1007/s00792-008-0184-4
GCA_900176145.1	Thermophiles	Acidophiles	3861015	Bacteria	Firmicutes_E	Sulfobacillia	Sulfobacillales	Sulfobacillaceae	Sulfobacillus	*Sulfobacillus thermosulfidooxidans*	28034	*Sulfobacillus thermosulfidooxidans*	https://www.ncbi.nlm.nih.gov/pmc/articles/PMC7000362/
GCA_001280565.1	Thermophiles	Acidophiles	3828023	Bacteria	Firmicutes_E	Sulfobacillia	Sulfobacillales	Sulfobacillaceae	Sulfobacillus	*Sulfobacillus thermosulfidooxidans_A*	28034	*Sulfobacillus thermosulfidooxidans*	https://www.ncbi.nlm.nih.gov/pmc/articles/PMC7000362/
GCA_000237975.1		Acidophiles	3557831	Bacteria	Firmicutes_E	Sulfobacillia	Sulfobacillales	Sulfobacillaceae	Sulfobacillus_A	*Sulfobacillus_A acidophilus*	1051632	*Sulfobacillus acidophilus TPY*	https://doi.org/10.4056/sigs.2736042
GCA_003023695.1		Acidophiles	4108624	Bacteria	Firmicutes_E	Sulfobacillia	Sulfobacillales	Sulfobacillaceae	Sulfobacillus_C	*Sulfobacillus_C benefaciens_A*	453960	*Sulfobacillus benefaciens*	https://doi.org/10.1007/s00792-008-0184-4
GCA_003967175.1	Thermophiles	Acidophiles	2353189	Archaea	Thermoproteota	Thermoproteia	Sulfolobales	Sulfolobaceae	Sulfodiicoccus	*Sulfodiicoccus acidiphilus*	1670455	*Sulfodiicoccus acidiphilus*	https://doi.org/10.1099/ijsem.0.001881
GCA_000012285.1	Thermophiles	Acidophiles	2225959	Archaea	Thermoproteota	Thermoproteia	Sulfolobales	Sulfolobaceae	Sulfolobus	*Sulfolobus acidocaldarius*	2285	*Sulfolobus acidocaldarius*	https://pubmed.ncbi.nlm.nih.gov/2478523/
GCA_000508305.1	Thermophiles	Acidophiles	2061920	Archaea	Thermoproteota	Thermoproteia	Sulfolobales	Sulfolobaceae	Sulfolobus	*Sulfolobus acidocaldarius_A*	2285	*Sulfolobus acidocaldarius*	https://pubmed.ncbi.nlm.nih.gov/2478523/
GCA_009938015.1		Acidophiles	3452774	Bacteria	Proteobacteria	Gammaproteobacteria	Burkholderiales	Sulfuriferulaceae	Sulfuriferula	*Sulfuriferula plumbiphila*	171865	*Sulfuriferula plumbiphila*	https://www.microbiologyresearch.org/content/journal/ijsem/10.1099/ijsem.0.004166
GCA_009729055.1	Thermophiles	Acidophiles	2803915	Archaea	Thermoproteota	Thermoproteia	Sulfolobales	Sulfolobaceae	Sulfurisphaera	*Sulfurisphaera ohwakuensis*	69656	*Sulfurisphaera ohwakuensis*	https://doi.org/10.1099/00207713-48-2-451
GCA_000011205.1	Hyperthermophiles		2694756	Archaea	Thermoproteota	Thermoproteia	Sulfolobales	Sulfolobaceae	Sulfurisphaera	*Sulfurisphaera tokodaii*	273063	*Sulfurisphaera tokodaii str. 7*	https://pubmed.ncbi.nlm.nih.gov/30304218/
GCA_002754935.1	Thermophiles		2659739	Bacteria	Cyanobacteria	Cyanobacteriia	Thermosynechococcales	Thermosynechococcaceae	Synechococcus_A	*Synechococcus_A lividus*	1917166	*Thermostichus lividus PCC 6715*	https://pubmed.ncbi.nlm.nih.gov/35602029/
GCA_004634385.1	Hyperthermophiles		3039102	Bacteria	Firmicutes_E	Thermaerobacteria	Thermaerobacterales	Thermaerobacteraceae	Thermaerobacter	*Thermaerobacter sp004634385*	2546351	*Thermaerobacter sp. FW80*	http://www.kjom.org/journal/view.html?uid=176&pn=lastest&vmd=Full
GCA_000183545.3		Alkaliphiles	2888741	Bacteria	Firmicutes_E	Thermaerobacteria	Thermaerobacterales	Thermaerobacteraceae	Thermaerobacter	*Thermaerobacter subterraneus*	867903	*Thermaerobacter subterraneus DSM 13965*	https://pubmed.ncbi.nlm.nih.gov/12054240/
GCA_900176005.1	Thermophiles		3334124	Bacteria	Firmicutes_B	Moorellia	Moorellales	Moorellaceae	Thermanaeromonas	*Thermanaeromonas toyohensis*	698762	*Thermanaeromonas toyohensis ToBE*	https://doi.org/10.1099/00207713-52-5-1675
GCA_003722315.1	Thermophiles		2911280	Bacteria	Firmicutes_A	Thermoanaerobacteria	Thermoanaerobacterales	Thermoanaerobacteraceae	Thermoanaerobacter	*Thermoanaerobacter ethanolicus*	509192	*Thermoanaerobacter ethanolicus JW 200*	https://doi.org/10.1128/aem.01773-20
GCA_000763575.1	Thermophiles		2397824	Bacteria	Firmicutes_A	Thermoanaerobacteria	Thermoanaerobacterales	Thermoanaerobacteraceae	Thermoanaerobacter	*Thermoanaerobacter kivui*	2325	*Thermoanaerobacter kivui*	https://bacdive.dsmz.de/strain/16823
GCA_000019085.1	Thermophiles		2362816	Bacteria	Firmicutes_A	Thermoanaerobacteria	Thermoanaerobacterales	Thermoanaerobacteraceae	Thermoanaerobacter	*Thermoanaerobacter pseudethanolicus*	509193	*Thermoanaerobacter brockii subsp. finnii Ako-1*	https://doi.org/10.1099/00207713-45-4-783
GCA_001310975.1	Thermophiles		2452939	Bacteria	Firmicutes_A	Thermoanaerobacteria	Thermoanaerobacterales	Thermoanaerobacteraceae	Thermoanaerobacter	*Thermoanaerobacter thermocopriae*	580331	*Thermoanaerobacter thermocopriae*	https://bacdive.dsmz.de/strain/165540
GCA_900129115.1	Thermophiles	Acidophiles	2409754	Bacteria	Firmicutes_A	Thermoanaerobacteria	Thermoanaerobacterales	Thermoanaerobacteraceae	Thermoanaerobacter	*Thermoanaerobacter uzonensis*	1123369	*Thermoanaerobacter uzonensis DSM 18761*	https://doi.org/10.1099/ijs.0.65343-0
GCA_000147695.3	Thermophiles		2785056	Bacteria	Firmicutes_A	Thermoanaerobacteria	Thermoanaerobacterales	Thermoanaerobacteraceae	Thermoanaerobacter	*Thermoanaerobacter wiegelii*	697303	*Thermoanaerobacter wiegelii Rt8.B1*	https://doi.org/10.1007/s007920070014
GCA_000145615.1	Thermophiles	Acidophiles	2785752	Bacteria	Firmicutes_A	Thermoanaerobacteria	Thermoanaerobacterales	Thermoanaerobacteraceae	Thermoanaerobacterium	*Thermoanaerobacterium thermosaccharolyticum*	1517	*Thermoanaerobacterium thermosaccharolyticum*	https://bacdive.dsmz.de/strain/18044
GCA_001516585.1	Thermophiles		1876950	Archaea	Thermoproteota	Thermoproteia	Thermoproteales	Thermocladiaceae	Thermocladium	*Thermocladium sp001516585*	1714261	*Thermocladium sp. ECH_B*	https://doi.org/10.1099/00207713-48-3-879
GCA_002214465.1	Hyperthermophiles		1922421	Archaea	Methanobacteriota_B	Thermococci	Thermococcales	Thermococcaceae	Thermococcus	*Thermococcus barossii*	54077	*Thermococcus barossii*	https://doi.org/10.1016/s0723-2020(98)80007-6
GCA_002214365.1	Hyperthermophiles		1866819	Archaea	Methanobacteriota_B	Thermococci	Thermococcales	Thermococcaceae	Thermococcus	*Thermococcus celer*	1293037	*Thermococcus celer Vu 13 = JCM 8558*	https://doi.org/10.1016/s0167-7012(99)00092-5
GCA_001484195.1	Hyperthermophiles		2337139	Archaea	Methanobacteriota_B	Thermococci	Thermococcales	Thermococcaceae	Thermococcus	*Thermococcus celericrescens*	2598455	*Thermococcus celericrescens*	https://doi.org/10.1099/ijs.0.64597-0
GCA_000265525.1	Hyperthermophiles		1950313	Archaea	Methanobacteriota_B	Thermococci	Thermococcales	Thermococcaceae	Thermococcus	*Thermococcus cleftensis*	163003	*Thermococcus cleftensis CL1*	https://doi.org/10.1099/ijs.0.066100-0
GCA_000769655.1	Hyperthermophiles		2126164	Archaea	Methanobacteriota_B	Thermococci	Thermococcales	Thermococcaceae	Thermococcus	*Thermococcus eurythermalis*	1505907	*Thermococcus eurythermalis*	https://doi.org/10.1099/ijs.0.067942-0
GCA_000022365.1	Hyperthermophiles		2045438	Archaea	Methanobacteriota_B	Thermococci	Thermococcales	Thermococcaceae	Thermococcus	*Thermococcus gammatolerans*	593117	*Thermococcus gammatolerans EJ3*	https://doi.org/10.1099/ijs.0.02503-0
GCA_002214385.1	Thermophiles		1674122	Archaea	Methanobacteriota_B	Thermococci	Thermococcales	Thermococcaceae	Thermococcus	*Thermococcus gorgonarius*	71997	*Thermococcus gorgonarius W-12*	https://doi.org/10.1099/00207713-48-1-23
GCA_000816105.1	Hyperthermophiles		1920914	Archaea	Methanobacteriota_B	Thermococci	Thermococcales	Thermococcaceae	Thermococcus	*Thermococcus guaymasensis*	1432656	*Thermococcus guaymasensis DSM 11113*	https://doi.org/10.1099/00207713-48-4-1181
GCA_000009965.1	Hyperthermophiles		2088737	Archaea	Methanobacteriota_B	Thermococci	Thermococcales	Thermococcaceae	Thermococcus	*Thermococcus kodakarensis*	69014	*Thermococcus kodakarensis KOD1*	https://doi.org/10.1007/978-1-0716-2445-6_5
GCA_000585495.1	Hyperthermophiles		1976356	Archaea	Methanobacteriota_B	Thermococci	Thermococcales	Thermococcaceae	Thermococcus	*Thermococcus nautili*	195522	*Thermococcus nautili 30-1*	https://doi.org/10.1099/ijs.0.060376-0
GCA_000018365.1	Hyperthermophiles	Alkaliphiles	1847607	Archaea	Methanobacteriota_B	Thermococci	Thermococcales	Thermococcaceae	Thermococcus	*Thermococcus onnurineus*	523850	*Thermococcus onnurineus NA1*	https://bacdive.dsmz.de/strain/161372
GCA_002214485.1	Thermophiles		1785673	Archaea	Methanobacteriota_B	Thermococci	Thermococcales	Thermococcaceae	Thermococcus	*Thermococcus pacificus*	71998	*Thermococcus pacificus P-4*	https://doi.org/10.1099/00207713-48-1-23
GCA_001592435.1	Thermophiles		1896106	Archaea	Methanobacteriota_B	Thermococci	Thermococcales	Thermococcaceae	Thermococcus	*Thermococcus peptonophilus*	53952	*Thermococcus peptonophilus*	https://pubmed.ncbi.nlm.nih.gov/7545383/
GCA_001647085.1	Hyperthermophiles		1928800	Archaea	Methanobacteriota_B	Thermococci	Thermococcales	Thermococcaceae	Thermococcus	*Thermococcus piezophilus*	1712654	*Thermococcus piezophilus*	https://doi.org/10.1016/j.syapm.2016.08.003
GCA_002214565.1	Hyperthermophiles		1868990	Archaea	Methanobacteriota_B	Thermococci	Thermococcales	Thermococcaceae	Thermococcus	*Thermococcus radiotolerans*	187880	*Thermococcus radiotolerans EJ2*	https://doi.org/10.1007/s00792-004-0380-9
GCA_000151205.2	Hyperthermophiles		2086428	Archaea	Methanobacteriota_B	Thermococci	Thermococcales	Thermococcaceae	Thermococcus	*Thermococcus sp000151205*	246969	*Thermococcus sp. AM4*	https://doi.org/10.1128/jb.06259-11
GCA_900198835.1	Hyperthermophiles		2155760	Archaea	Methanobacteriota_B	Thermococci	Thermococcales	Thermococcaceae	Thermococcus	*Thermococcus sp900198835*	2016361	*Thermococcus henrietii*	https://doi.org/10.1099/ijsem.0.004895
GCA_017873335.1	Hyperthermophiles		2034780	Archaea	Methanobacteriota_B	Thermococci	Thermococcales	Thermococcaceae	Thermococcus	*Thermococcus stetteri*	49900	*Thermococcus stetteri*	https://doi.org/10.1093/protein/10.8.905
GCA_002214545.1	Hyperthermophiles		2065932	Archaea	Methanobacteriota_B	Thermococci	Thermococcales	Thermococcaceae	Thermococcus	*Thermococcus thioreducens*	277988	*Thermococcus thioreducens*	https://doi.org/10.1099/ijs.0.65057-0
GCA_000258515.1	Hyperthermophiles		1764559	Archaea	Methanobacteriota_B	Thermococci	Thermococcales	Thermococcaceae	Thermococcus	*Thermococcus zilligii*	1151117	*Thermococcus zilligii AN1*	https://doi.org/10.1128/jb.182.16.4632-4636.2000
GCA_000246985.3	Hyperthermophiles		2215172	Archaea	Methanobacteriota_B	Thermococci	Thermococcales	Thermococcaceae	Thermococcus_A	*Thermococcus_A litoralis*	523849	*Thermococcus litoralis DSM 5473*	https://bacdive.dsmz.de/strain/16862
GCA_000022545.1	Hyperthermophiles		1845800	Archaea	Methanobacteriota_B	Thermococci	Thermococcales	Thermococcaceae	Thermococcus_A	*Thermococcus_A sibiricus*	604354	*Thermococcus sibiricus MM 739*	https://doi.org/10.3390/ijms22189894
GCA_000151105.2	Hyperthermophiles		2064237	Archaea	Methanobacteriota_B	Thermococci	Thermococcales	Thermococcaceae	Thermococcus_B	*Thermococcus_B barophilus*	391623	*Thermococcus barophilus*	https://doi.org/10.1099/00207713-49-2-351
GCA_000025605.1	Thermophiles		1500577	Bacteria	Aquificota	Aquificae	Aquificales	Aquificaceae	Thermocrinis	*Thermocrinis albus*	638303	*Thermocrinis albus DSM 14484*	https://link.springer.com/article/10.1007/s00792-001-0259-y
GCA_000512735.1	Hyperthermophiles		1521037	Bacteria	Aquificota	Aquificae	Aquificales	Aquificaceae	Thermocrinis	*Thermocrinis ruber*	75906	*Thermocrinis ruber*	https://doi.org/10.1128/aem.64.10.3576-3583.1998
GCA_900142435.1	Thermophiles		1367921	Bacteria	Aquificota	Aquificae	Aquificales	Aquificaceae	Thermocrinis_A	*Thermocrinis_A minervae*	381751	*Thermocrinis minervae*	https://pubmed.ncbi.nlm.nih.gov/19651724/
GCA_003057965.1	Thermophiles	Acidophiles	1774794	Bacteria	Thermodesulfobiota	Thermodesulfobiia	Thermodesulfobiales	Thermodesulfobiaceae	Thermodesulfobium	*Thermodesulfobium acidiphilum*	1794699	*Thermodesulfobium acidiphilum*	https://doi.org/10.1099/ijsem.0.001745
GCA_000212395.1	Thermophiles		1898865	Bacteria	Thermodesulfobiota	Thermodesulfobiia	Thermodesulfobiales	Thermodesulfobiaceae	Thermodesulfobium	*Thermodesulfobium narugense*	747365	*Thermodesulfobium narugense DSM 14796*	https://doi.org/10.1007/s00792-003-0320-0
GCA_000446015.1	Hyperthermophiles		1750259	Archaea	Thermoproteota	Thermoproteia	Thermofilales	Thermofilaceae	Thermofilum_B	*Thermofilum_B adornatum*	1365176	*Thermofilum adornatus 1910b*	https://doi.org/10.3389/fmicb.2019.02972
GCA_000015225.1	Hyperthermophiles		1813393	Archaea	Thermoproteota	Thermoproteia	Thermofilales	Thermofilaceae	Thermofilum_B	*Thermofilum_B pendens_A*	368408	*Thermofilum pendens Hrk 5*	https://doi.org/10.1007/s00253-009-2190-6
GCA_000264495.1	Hyperthermophiles		1356318	Archaea	Thermoproteota	Thermoproteia	Sulfolobales	Desulfurococcaceae	Thermogladius	*Thermogladius calderae*	1184251	*Thermogladius calderae 1633*	https://doi.org/10.1099/ijsem.0.000916
GCA_000195915.1	Thermophiles	Acidophiles	1564906	Archaea	Thermoplasmatota	Thermoplasmata	Thermoplasmatales	Thermoplasmataceae	Thermoplasma	*Thermoplasma acidophilum*	273075	*Thermoplasma acidophilum DSM 1728*	https://bacdive.dsmz.de/strain/17017
GCA_000011185.1	Thermophiles	Acidophiles	1584804	Archaea	Thermoplasmatota	Thermoplasmata	Thermoplasmatales	Thermoplasmataceae	Thermoplasma	*Thermoplasma volcanium*	273116	*Thermoplasma volcanium GSS1*	https://doi.org/10.1128/jb.178.3.902-905.1996
GCA_000253055.1	Hyperthermophiles	Acidophiles	1841542	Archaea	Thermoproteota	Thermoproteia	Thermoproteales	Thermoproteaceae	Thermoproteus	*Thermoproteus tenax*	768679	*Thermoproteus tenax Kra 1*	https://bacdive.dsmz.de/strain/17031
GCA_000193375.1	Thermophiles	Acidophiles	1936063	Archaea	Thermoproteota	Thermoproteia	Thermoproteales	Thermoproteaceae	Thermoproteus	*Thermoproteus uzoniensis*	999630	*Thermoproteus uzoniensis 768-20*	https://doi.org/10.1128/jb.00409-11
GCA_000008545.1	Hyperthermophiles		1860725	Bacteria	Thermotogota	Thermotogae	Thermotogales	Thermotogaceae	Thermotoga	*Thermotoga maritima*	243274	*Thermotoga maritima MSB8*	https://bacdive.dsmz.de/strain/17060
GCA_000018945.1	Hyperthermophiles		1884562	Bacteria	Thermotogota	Thermotogae	Thermotogales	Thermotogaceae	Thermotoga	*Thermotoga neapolitana*	309803	*Thermotoga neapolitana DSM 4359*	https://bacdive.dsmz.de/strain/17061

GCA_000016785.1	Hyperthermophiles		1823511	Bacteria	Thermotogota	Thermotogae	Thermotogales	Thermotogaceae	Thermotoga	*Thermotoga petrophila*	590168	*Thermotoga petrophila*	https://doi.org/10.1099/00207713-51-5-1901
GCA_000744885.1	Thermophiles		2160855	Bacteria	Deinococcota	Deinococci	Deinococcales	Thermaceae	Thermus	*Thermus amyloliquefaciens*	1449080	*Thermus amyloliquefaciens*	https://doi.org/10.1099/ijs.0.000289
GCA_000423905.1	Thermophiles		2165150	Bacteria	Deinococcota	Deinococci	Deinococcales	Thermaceae	Thermus	*Thermus antranikianii*	1123386	*Thermus antranikianii DSM 12462*	https://bacdive.dsmz.de/strain/16730
GCA_001280255.1	Thermophiles		2248795	Bacteria	Deinococcota	Deinococci	Deinococcales	Thermaceae	Thermus	*Thermus aquaticus*	271	*Thermus aquaticus*	https://doi.org/10.1128/jb.98.1.289-297.1969
GCA_900102145.1	Thermophiles		2442297	Bacteria	Deinococcota	Deinococci	Deinococcales	Thermaceae	Thermus	*Thermus arciformis*	482827	*Thermus arciformis*	https://doi.org/10.1099/ijs.0.007690-0
GCA_001880325.1	Thermophiles		2388273	Bacteria	Deinococcota	Deinococci	Deinococcales	Thermaceae	Thermus	*Thermus brockianus*	56956	*Thermus brockianus*	https://bacdive.dsmz.de/strain/161175
GCA_003336745.1	Thermophiles		2163786	Bacteria	Deinococcota	Deinococci	Deinococcales	Thermaceae	Thermus	*Thermus caldifontis*	1930763	*Thermus caldifontis*	https://doi.org/10.1099/ijsem.0.002037
GCA_000745065.1	Thermophiles		2218114	Bacteria	Deinococcota	Deinococci	Deinococcales	Thermaceae	Thermus	*Thermus calditerrae*	1330700	*Thermus calditerrae*	https://doi.org/10.1099/ijs.0.056838-0
GCA_000376265.1	Thermophiles		2225983	Bacteria	Deinococcota	Deinococci	Deinococcales	Thermaceae	Thermus	*Thermus igniterrae*	1123388	*Thermus igniterrae ATCC 700962*	https://doi.org/10.1099/00207713-50-1-209
GCA_000421625.1	Thermophiles	Acidophiles	2263010	Bacteria	Deinococcota	Deinococci	Deinococcales	Thermaceae	Thermus	*Thermus islandicus*	1123389	*Thermus islandicus DSM 21543*	https://doi.org/10.1099/ijs.0.007013-0
GCA_000373145.1	Thermophiles		2260954	Bacteria	Deinococcota	Deinococci	Deinococcales	Thermaceae	Thermus	*Thermus oshimai*	1123390	*Thermus oshimai DSM 12092*	https://doi.org/10.1099/00207713-46-2-403
GCA_001535545.1	Hyperthermophiles		2016098	Bacteria	Deinococcota	Deinococci	Deinococcales	Thermaceae	Thermus	*Thermus parvatiensis*	456163	*Thermus parvatiensis*	https://pubmed.ncbi.nlm.nih.gov/26543260/
GCA_000381045.1	Thermophiles		2070699	Bacteria	Deinococcota	Deinococci	Deinococcales	Thermaceae	Thermus	*Thermus scotoductus*	1123391	*Thermus scotoductus DSM 8553*	https://bacdive.dsmz.de/strain/16724
GCA_000744175.1	Thermophiles		2562314	Bacteria	Deinococcota	Deinococci	Deinococcales	Thermaceae	Thermus	*Thermus tengchongensis*	1214928	*Thermus tengchongensis*	https://pubmed.ncbi.nlm.nih.gov/23104072/
GCA_002964845.1	Thermophiles		2261036	Bacteria	Deinococcota	Deinococci	Deinococcales	Thermaceae	Thermus	*Thermus tenuipuniceus*	2078690	*Thermus tenuipuniceus*	https://bacdive.dsmz.de/strain/163768
GCA_000091545.1	Thermophiles		2116056	Bacteria	Deinococcota	Deinococci	Deinococcales	Thermaceae	Thermus	*Thermus thermophilus*	274	*Thermus thermophilus*	https://doi.org/10.1016/j.ymben.2017.10.007
GCA_002355995.1	Thermophiles		2140665	Bacteria	Deinococcota	Deinococci	Deinococcales	Thermaceae	Thermus	*Thermus thermophilus_C*	274	*Thermus thermophilus*	https://doi.org/10.1016/j.ymben.2017.10.007
GCA_000771745.2	Thermophiles		2386081	Bacteria	Deinococcota	Deinococci	Deinococcales	Thermaceae	Thermus_A	*Thermus_A filiformis*	276	*Thermus filiformis*	https://bacdive.dsmz.de/strain/16723
GCA_000376425.1	Thermophiles		3609948	Bacteria	Proteobacteria	Gammaproteobacteria	Burkholderiales	Thiobacillaceae	Thiobacillus	*Thiobacillus denitrificans*	1123392	*Thiobacillus denitrificans DSM 12475*	https://bacdive.dsmz.de/strain/6143
GCA_000012745.1	Thermophiles		2909809	Bacteria	Proteobacteria	Gammaproteobacteria	Burkholderiales	Thiobacillaceae	Thiobacillus	*Thiobacillus denitrificans_B*	292415	*Thiobacillus denitrificans ATCC 25259*	https://bacdive.dsmz.de/strain/6143
GCA_900113635.1		Alkaliphiles	3083807	Bacteria	Firmicutes_A	Clostridia	Peptostreptococcales	Tindalliaceae	Tindallia	*Tindallia magadiensis*	69895	*Tindallia magadiensis*	https://doi.org/10.1007/s002849900345
GCA_000092425.1	Thermophiles	Alkaliphiles	3260398	Bacteria	Deinococcota	Deinococci	Deinococcales	Trueperaceae	Truepera	*Truepera radiovictrix*	649638	*Truepera radiovictrix DSM 17093*	https://pubmed.ncbi.nlm.nih.gov/15927420/
GCA_000148385.1	Hyperthermophiles	Acidophiles	2374137	Archaea	Thermoproteota	Thermoproteia	Thermoproteales	Thermocladiaceae	Vulcanisaeta	*Vulcanisaeta distributa*	572478	*Vulcanisaeta distributa DSM 14429*	https://doi.org/10.1099/00207713-52-4-1097
GCA_000190315.1	Hyperthermophiles		2298983	Archaea	Thermoproteota	Thermoproteia	Thermoproteales	Thermocladiaceae	Vulcanisaeta	*Vulcanisaeta moutnovskia*	985053	*Vulcanisaeta moutnovskia 768-28*	https://doi.org/10.1128/jb.00237-11
GCA_014646555.1	Hyperthermophiles	Acidophiles	2424395	Archaea	Thermoproteota	Thermoproteia	Thermoproteales	Thermocladiaceae	Vulcanisaeta	*Vulcanisaeta souniana*	1293586	*Vulcanisaeta souniana JCM 11219*	https://doi.org/10.1099/00207713-52-4-1097
GCA_001748385.1	Hyperthermophiles	Acidophiles	2022594	Archaea	Thermoproteota	Thermoproteia	Thermoproteales	Thermocladiaceae	Vulcanisaeta_B	*Vulcanisaeta_B thermophila*	867917	*Vulcanisaeta thermophila*	https://doi.org/10.1099/ijs.0.065862-0
GCA_002094855.1	Psychrophiles		4780723	Bacteria	Bacteroidota	Bacteroidia	Flavobacteriales	Flavobacteriaceae	Zunongwangia	*Zunongwangia atlantica*	1185767	*Zunongwangia atlantica 22II14-10F7*	https://doi.org/10.1099/ijs.0.054007-0
GCA_000023465.1	Psychrophiles		5128187	Bacteria	Bacteroidota	Bacteroidia	Flavobacteriales	Flavobacteriaceae	Zunongwangia	*Zunongwangia profunda*	655815	*Zunongwangia profunda SM-A87*	https://doi.org/10.1186%2F1471-2164-11-247
