# Supplementary material for: Environment and taxonomy shape the genomic signature of prokaryotic extremophiles
Source: Sci Rep. 2023 Sep 26;13:16105. doi: 10.1038/s41598-023-42518-y (PMC10522608; doi:10.1038/s41598-023-42518-y)
Supplement: Supplementary file 6 — Supplementary Information 6. [file 41598_2023_42518_MOESM6_ESM.pdf]

Supplementary Table S2-Dataset Metadata			
**Assembly Accession**	**Submission Date**	**Last Updated**	**Submission Organization**
GCF_000091545.1	2004/11/15	2020/03/06	National Institute of Advanced Industrial Science and Technology, Japan
GCF_000236925.1	2011/11/10	2013/09/23	The Wellcome Trust Sanger Institute
GCF_000025685.1	2010/03/23	2016/11/29	TIGR
GCF_002945325.1	2018/02/12	2018/03/02	Goettingen Genomics Laboratory
GCF_000196795.1	2010/06/21	2016/11/29	Division of Environmental Science and Ecological Engineering, Korea University
GCF_000009965.1	2005/01/05	2016/11/29	Kyoto University, Japan
GCF_009662475.1	2019/11/14	2019/11/16	Shandong University(Qingdao)
GCF_000022325.1	2009/02/04	2016/11/29	US DOE Joint Genome Institute (JGI-PGF)
GCF_000027145.1	2010/02/18	2016/11/29	PathoGenoMik
GCF_000816085.1	2015/01/07	2015/01/23	University of Missouri - Columbia
GCF_000304355.2	2014/02/04	2015/07/16	irena.maus
GCF_001277215.2	2016/01/13	2016/01/17	Nestle Institute of Health Sciences
GCF_001975665.1	2017/01/30	2017/02/03	Kyung Hee University
GCF_000014885.1	2008/11/13	2016/11/29	James D. Watson Institute of Genome Sciences
GCF_000016065.1	2007/03/07	2016/11/29	US DOE Joint Genome Institute
GCF_000091665.1	1999/12/22	2016/06/03	TIGR
GCF_000018465.1	2007/11/15	2019/08/31	US DOE Joint Genome Institute (JGI-PGF)
GCF_000195915.1	2003/05/06	2016/11/29	Max-Plank-Institute
GCF_000172995.2	2010/11/22	2016/11/29	US DOE Joint Genome Institute (JGI-PGF)
GCF_000007225.1	2002/01/24	2019/01/29	UCLA/CalTech
GCF_001277235.1	2015/09/01	2015/09/02	Nestle Institute of Health Sciences
GCF_000202835.1	2011/03/01	2017/04/07	National Institute of Technology and Evaluation
GCF_000024845.1	2009/11/03	2016/11/29	US DOE Joint Genome Institute (JGI-PGF)
GCF_000023965.1	2009/09/03	2016/11/29	US DOE Joint Genome Institute (JGI-PGF)
GCF_000024285.1	2009/09/09	2016/11/29	US DOE Joint Genome Institute (JGI-PGF)
GCF_000022205.1	2009/02/04	2021/04/08	US DOE Joint Genome Institute (JGI-PGF)
GCF_003012915.1	2018/03/21	2018/03/23	Illinois Institute of Technology

GCF_008931805.1	2019/10/09	2019/10/13	University hospital Essen
GCF_000968535.2	2011/10/14	2021/12/27	CEA/DSV/IG/Genoscope
GCF_000230715.2	2012/12/10	2012/12/21	DOE Joint Genome Institute
GCF_000196895.1	2010/06/23	2016/11/29	Kyung Hee University
GCF_000017945.1	2007/08/29	2016/11/29	US DOE Joint Genome Institute
GCF_001277175.1	2015/09/01	2015/09/02	Nestle Institute of Health Sciences
GCF_000014185.1	2006/06/09	2016/11/29	DOE Joint Genome Institute
GCF_000698785.1	2014/06/06	2016/12/26	University of Vienna
GCF_000019085.1	2008/02/05	2021/06/08	US DOE Joint Genome Institute
GCF_000018945.1	2009/01/27	2016/11/29	Genotech Corp.
GCF_000018365.1	2008/11/06	2016/11/29	Korea Ocean Research & Development Institute
GCF_000011125.1	2006/10/11	2019/08/31	NITE
GCF_001277195.1	2015/09/01	2015/09/02	Nestle Institute of Health Sciences
GCF_000016545.1	2007/04/19	2016/11/29	US DOE Joint Genome Institute
GCF_000235405.2	2012/04/02	2016/11/30	DOE Joint Genome Institute
GCF_000151105.2	2010/12/17	2016/11/29	Moore Foundation
GCF_000195935.2	2003/05/06	2014/02/03	Genoscope
GCF_002075795.1	2017/03/31	2017/04/03	Korea Polar Research Institute
GCF_014784055.1	2020/09/29	2020/09/29	Kyung Hee University
GCF_001971705.1	2017/01/26	2017/02/15	Heilongjiang Academy
GCF_003344925.1	2018/07/30	2018/08/01	Shandong University
GCF_001634285.1	2016/04/29	2016/08/18	Los Alamos National Laboratory
GCF_000470655.1	2013/08/01	2021/02/10	MPI BREMEN
GCF_003019255.1	2018/03/26	2018/03/29	Illinois Institute of Technology
GCF_000214725.1	2011/05/24	2016/11/29	US DOE Joint Genome Institute
GCF_000763575.1	2014/10/06	2015/07/25	Georg-August-University Goettingen, Genomic and Applied Microbiology, Goettingen Genomics Laboratory
GCF_003574215.1	2018/06/21	2018/09/20	Department of Biotechnology, The University of Tokyo
GCF_001412615.1	2015/10/26	2015/10/27	Kyung Hee University
GCF_001889405.1	2016/12/06	2016/12/18	UBO

GCF_002078355.1	2017/04/04	2017/04/05	Max Planck Institute for Chemical Ecology
GCF_000023465.1	2010/04/21	2016/11/29	ShanDong University
GCF_000970285.1	2015/04/07	2015/06/26	University of Illinois at Urbana-Champaign
GCF_000015505.1	2007/01/04	2016/11/29	DOE Joint Genome Institute
GCF_000022565.1	2009/03/30	2016/11/29	J. Craig Venter Institute
GCF_000025625.1	2010/02/24	2016/11/29	US DOE Joint Genome Institute (JGI-PGF)
GCF_000012305.1	2005/07/20	2016/11/29	DOE Joint Genome Institute
GCF_000013725.1	2006/04/10	2016/11/29	DOE Joint Genome Institute
GCF_000145215.1	2010/08/11	2016/11/29	US DOE Joint Genome Institute
GCF_000025505.1	2010/02/16	2016/11/29	US DOE Joint Genome Institute
GCF_000504085.1	2013/10/23	2015/11/23	National Institute of Technology and Evaluation
GCF_000223395.1	2011/08/12	2012/11/23	DOE Joint Genome Institute
GCF_000007185.1	2002/04/03	2019/08/31	Fidelity Systems
GCF_000591035.1	2013/09/27	2019/09/01	Laboratory of Marine Microbiology, Division of Applied Biosciences, Graduate School of Agriculture, Kyoto University
GCF_000019185.1	2008/02/07	2016/11/29	US DOE Joint Genome Institute
GCF_001277255.1	2015/09/01	2015/09/02	Nestle Institute of Health Sciences
GCF_000025325.1	2010/01/19	2016/11/29	US DOE Joint Genome Institute (JGI-PGF)
GCF_000013445.1	2006/02/17	2016/11/29	DOE Joint Genome Institute
GCF_000969885.1	2015/04/07	2015/06/26	University of Illinois at Urbana-Champaign
GCF_000226295.1	2011/09/23	2016/11/30	The Pennsylvania State University
GCF_000148385.1	2010/09/22	2016/11/29	US DOE Joint Genome Institute (JGI-PGF)
GCF_000016385.1	2007/04/16	2019/01/29	DOE Joint Genome Institute
GCF_000022365.1	2009/06/12	2016/11/29	IGM/ Universit Paris-Sud, France
GCF_000020965.1	2008/09/26	2016/11/29	The J. Craig Venter Institute
GCF_000253055.1	2011/10/06	2012/10/11	University of Duisburg-Essen, Faculty of Chemistry, Molecular Enzyme Technology and Biochemistry
GCF_000016785.1	2007/05/18	2016/11/29	US DOE Joint Genome Institute
GCF_000186365.1	2011/01/20	2016/11/29	US DOE Joint Genome Institute (JGI-PGF)
GCF_000166095.1	2010/11/05	2016/11/29	US DOE Joint Genome Institute (JGI-PGF)

GCF_003430825.1	2018/08/28	2018/09/02	C.N.R. - Institute for Coastal Marine Environment (IAMC)
GCF_003201835.2	2020/03/10	2020/04/16	North Carolina State University
GCF_000585495.1	2014/03/05	2015/02/16	Institute de Genetique et Microbiologie
GCF_001535545.1	2016/01/25	2016/01/26	University of Delhi
GCF_000021645.1	2008/12/12	2020/03/06	US DOE Joint Genome Institute (JGI-PGF)
GCF_000875775.1	2015/02/13	2019/09/01	University of Vienna
GCF_000512735.1	2014/01/03	2015/02/04	JGI
GCF_000019525.1	2008/03/13	2016/11/29	US DOE Joint Genome Institute
GCF_000018025.1	2007/09/18	2016/11/29	US DOE Joint Genome Institute (JGI-PGF)
GCF_000018285.1	2007/10/04	2016/11/29	US DOE Joint Genome Institute
GCF_000970085.1	2015/04/07	2015/06/26	University of Illinois at Urbana-Champaign
GCF_000091325.1	2010/04/01	2016/11/29	Nara Institute of Science and Technology
GCF_000255115.2	2012/06/15	2012/10/18	DOE Joint Genome Institute
GCF_000015285.1	2006/12/22	2016/11/29	DOE Joint Genome Institute
GCF_000013765.1	2006/04/10	2016/11/29	DOE Joint Genome Institute
GCF_000153485.2	2012/10/09	2018/04/04	The Gordon and Betty Moore Foundation Marine Microbiology Initiative
GCF_000328685.1	2012/12/28	2012/12/31	JGI
GCF_000242595.2	2012/04/02	2016/11/30	DOE Joint Genome Institute
GCF_000092425.1	2010/05/28	2016/11/29	US DOE Joint Genome Institute (JGI-PGF)
GCF_000092125.1	2010/05/26	2016/11/29	US DOE Joint Genome Institute (JGI-PGF)
GCF_000023945.1	2009/08/27	2016/11/29	US DOE Joint Genome Institute (JGI-PGF)
GCF_000021965.1	2009/01/05	2016/11/29	US DOE Joint Genome Institute (JGI-PGF)
GCF_000166775.1	2010/11/17	2016/11/29	US DOE Joint Genome Institute (JGI-PGF)
GCF_000147875.1	2010/09/17	2019/12/27	US DOE Joint Genome Institute (JGI-PGF)
GCF_000327485.1	2012/12/21	2012/12/21	DOE Joint Genome Institute
GCF_000147695.2	2011/08/23	2018/04/04	US DOE Joint Genome Institute (JGI-PGF)
GCF_000166355.1	2010/11/10	2016/11/29	US DOE Joint Genome Institute
GCF_000011205.1	2004/05/11	2016/11/29	NITE
GCF_000026045.1	2005/09/28	2016/11/29	Max Planck Institute
GCF_000015825.1	2007/02/16	2016/11/29	DOE Joint Genome Institute

GCF_000015025.1	2006/11/09	2016/11/29	DOE Joint Genome Institute
GCF_000166335.1	2010/11/10	2016/11/29	US DOE Joint Genome Institute
GCF_000251105.1	2012/03/07	2016/11/30	China Agricultural University
GCF_000190315.1	2011/02/22	2016/11/29	Centre Bioengineering RAS
GCF_000246985.2	2013/08/13	2015/07/20	New England Biolabs, Inc.
GCF_000217995.1	2011/06/13	2016/11/30	US DOE Joint Genome Institute (JGI-PGF)
GCF_000024605.1	2009/10/08	2016/11/29	US DOE Joint Genome Institute
GCF_000018305.1	2007/10/05	2016/11/29	US DOE Joint Genome Institute (JGI-PGF)
GCF_000217815.1	2011/06/10	2016/11/30	US DOE Joint Genome Institute (JGI-PGF)
GCF_000025865.1	2010/03/31	2016/11/29	US DOE Joint Genome Institute (JGI-PGF)
GCF_000015805.1	2007/02/27	2020/10/25	DOE Joint Genome Institute
GCF_000152265.2	2013/06/26	2013/06/29	DOE Joint Genome Institute
GCF_000212395.1	2011/05/05	2016/11/29	US DOE Joint Genome Institute (JGI-PGF)
GCF_000014945.1	2006/10/25	2020/06/19	US DOE Joint Genome Institute
GCF_000725425.1	2014/07/14	2015/06/26	The third institute of oceanography of SOA,PRChina
GCF_000214415.1	2011/05/23	2016/11/29	US DOE Joint Genome Institute
GCF_000022545.1	2009/06/29	2016/11/29	Centre
GCF_000204925.1	2011/04/19	2016/11/29	Environmental Microbiology Research Center (EMRC)
GCF_000015205.1	2006/12/20	2016/11/29	DOE Joint Genome Institute
GCF_000015225.1	2006/12/18	2016/11/29	DOE Joint Genome Institute
GCF_000019805.1	2008/03/27	2019/08/31	US DOE Joint Genome Institute (JGI-PGF)
GCF_000024625.1	2009/10/15	2016/11/29	US DOE Joint Genome Institute (JGI-PGF)
GCF_000215995.1	2011/06/07	2016/11/30	Shanghai JiaoTong University
GCF_000015145.1	2007/01/22	2016/11/29	Univ. Copenhagen
GCF_000179575.2	2011/06/06	2018/04/04	US DOE Joint Genome Institute (JGI-PGF)
GCF_000145295.1	2010/08/11	2016/11/29	Georg-August-University Goettingen
GCF_000011185.1	2004/05/11	2016/11/29	National Institute of Advanced Industrial Science and Technology, Japan
GCF_000015945.1	2007/02/21	2020/05/06	US DOE Joint Genome Institute
GCF_000017185.1	2007/06/29	2016/11/29	US DOE Joint Genome Institute (JGI-PGF)
GCF_000317795.1	2012/12/10	2012/12/10	JGI

GCF_000025605.1	2010/02/19	2016/11/29	US DOE Joint Genome Institute (JGI-PGF)
GCF_000144915.1	2010/08/06	2016/11/29	Centre Bioengineering RAS
GCF_000023985.1	2009/08/28	2016/11/29	US DOE Joint Genome Institute (JGI-PGF)
GCF_001481685.1	2015/12/23	2016/12/26	Oak Ridge National Laboratory
GCF_002005305.1	2017/02/21	2017/02/23	Shanghai Jiao Tong University
GCF_017298635.1	2021/03/08	2022/05/13	Institute of Microbiology
GCF_007567505.1	2019/07/29	2019/08/03	Korea Institute of Ocean Science and Technology
GCF_003855395.1	2018/12/02	2018/12/04	Korea Polar Research Institute
GCF_000969905.1	2015/04/07	2015/06/26	University of Illinois at Urbana-Champaign
GCF_900465055.1	2018/06/24	2018/07/13	Genoscope CEA
GCF_002499975.2	2020/05/11	2020/05/13	NCAOR
GCF_001043175.1	2015/06/30	2015/07/02	Beijing Institute of Genomics, Chinese Academy of Sciences
GCF_002156705.1	2017/05/23	2017/05/25	Chinese Academy of Agricultural Sciences
GCF_003352165.1	2018/08/01	2018/08/03	institute of microbiology
GCF_001761365.1	2016/10/12	2016/10/14	Korea University
GCF_009938015.1	2020/01/17	2020/02/03	Hokkaido University
GCF_009392895.1	2019/10/30	2019/11/01	Jiangsu University
GCF_002952775.1	2018/02/01	2018/09/26	C.N.R. - Institute for Coastal Marine Environment (IAMC)
GCF_018228765.1	2021/05/03	2021/05/11	Incheon National University
GCF_003568865.1	2018/09/13	2018/09/18	South China Sea Institute of Oceanology, Chinese Academy of Sciences
GCF_009729055.1	2019/12/04	2019/12/09	North Carolina State University
GCF_013388255.1	2020/07/04	2020/07/04	National Chung Hsing University
GCF_002754935.1	2017/11/06	2017/11/09	Peking University Shenzhen Graduate School
GCF_900186985.1	2017/08/15	2021/12/15	SC
GCF_003201675.2	2020/03/10	2020/04/16	North Carolina State University
GCF_002813695.1	2017/12/07	2017/12/09	Japan Agency for Marine-Earth Science and technology
GCF_000234805.1	2011/11/10	2019/08/31	Centre Bioengineering RAS
GCF_003201765.2	2020/03/10	2020/04/16	North Carolina State University
GCF_009729015.1	2019/12/04	2019/12/09	North Carolina State University
GCF_000828675.1	2014/03/21	2015/01/30	National Institute of Technology and Evaluation

GCF_013343295.1	2020/06/15	2020/06/15	Institute of microbiology
GCF_900198835.1	2017/10/17	2017/10/28	Universite de Bretagne Occidentale
GCF_000769655.1	2014/10/28	2015/08/04	Laboratory of Marine Oceanography
GCF_002355215.1	2015/02/05	2019/01/29	Tsurumi University, School of Dental Medicine
GCF_000828655.1	2014/03/21	2015/01/30	National Institute of Technology and Evaluation
GCF_009729035.1	2019/12/04	2019/12/09	North Carolina State University
GCF_000265525.1	2012/06/11	2016/11/30	Kyung Hee University
GCF_001647085.1	2016/05/18	2016/08/19	Institut National des Sciences Appliquees de Lyon
GCF_002214465.1	2017/07/05	2017/07/07	Ecole Normale Superieure de Lyon
GCF_000816105.1	2015/01/07	2016/03/28	University of Waterloo
GCF_002214565.1	2017/07/05	2017/07/07	Ecole Normale Superieure de Lyon
GCF_002214365.1	2017/07/05	2017/07/07	Ecole Normale Superieure de Lyon
GCF_001592435.1	2016/03/15	2016/03/28	Ecole Normale Superieure de Lyon
GCF_000956175.1	2015/03/19	2015/06/26	University of Vienna
GCF_002214485.1	2017/07/05	2017/07/07	Ecole Normale Superieure de Lyon
GCF_003057965.1	2018/04/19	2018/04/25	Immanuel Kant Baltic Federal University
GCF_001006045.1	2015/05/11	2015/06/26	Michigan State University
GCF_002214385.1	2017/07/05	2017/07/07	Ecole Normale Superieure de Lyon
GCF_000739065.1	2014/08/12	2015/06/26	Kyung Hee University
GCF_013340765.1	2019/05/09	2020/06/14	Japan Collection of Microorganisms
GCF_000264495.1	2012/05/25	2016/11/30	Centre Bioengineering RAS
GCF_002215585.1	2017/07/06	2017/07/08	Hubei Polytechnic University
GCF_003534205.1	2017/03/13	2019/09/18	Technische Universitaet Muenchen
GCF_000970265.1	2015/04/07	2015/06/26	University of Illinois at Urbana-Champaign
GCF_000235565.1	2011/11/16	2016/11/30	State Key Laboratory of Microbial Resources, Institute of Microbiology, Chinese Academy of Sciences
GCF_007475525.1	2019/07/25	2019/07/31	University of Bergen
GCF_000193375.1	2011/03/28	2016/11/29	Centre Bioengineering RAS
GCF_001606025.1	2016/04/04	2016/04/07	Korea Polar Research Institute
GCF_001298525.1	2015/09/24	2016/03/28	University of Malaya

GCF_002116695.1	2017/05/01	2019/09/01	Cental South University, China
GCF_002761295.1	2017/11/07	2017/11/09	Macumba
GCF_004117075.1	2019/01/29	2019/02/01	Kyungpook National University
GCF_900176005.1	2017/04/06	2017/04/17	DOE - JOINT GENOME INSTITUTE
GCF_009735625.1	2019/11/28	2019/12/11	Georg-August-University Goettingen
GCF_900142435.1	2016/12/02	2017/03/18	DOE - JOINT GENOME INSTITUTE
GCF_000243255.1	2012/01/24	2016/11/30	JGI
GCF_000275865.1	2012/07/13	2012/07/23	DOE Joint Genome Institute
GCF_004346035.1	2019/03/11	2019/03/16	DOE Joint Genome Institute
GCF_900458435.1	2018/08/01	2018/08/07	SC
GCF_900101915.1	2016/10/21	2017/07/29	DOE - JOINT GENOME INSTITUTE
GCF_900176145.1	2017/04/07	2017/04/17	DOE - JOINT GENOME INSTITUTE
GCF_014196195.1	2020/08/14	2020/08/17	DOE Joint Genome Institute
GCF_900107035.1	2016/10/22	2016/12/22	DOE - JOINT GENOME INSTITUTE
GCF_900129125.1	2016/12/03	2016/12/18	DOE - JOINT GENOME INSTITUTE
GCF_000376425.1	2013/04/20	2014/07/12	DOE Joint Genome Institute
GCF_006716115.1	2019/07/08	2019/07/12	DOE Joint Genome Institute
GCF_004123295.1	2019/01/30	2019/02/07	Sun Yat-sen University
GCF_010119205.1	2020/02/03	2020/02/10	Anhui Normal University
GCF_900100875.1	2016/10/21	2017/07/29	DOE - JOINT GENOME INSTITUTE
GCF_003185895.1	2018/06/04	2018/06/07	Institute of Microbiology, Chinese Academy of Sciences
GCF_900110455.1	2016/10/31	2016/12/23	DOE - JOINT GENOME INSTITUTE
GCF_000771745.2	2015/02/24	2015/03/20	Centro Nacional de Pesquisa em Energia e Materiais
GCF_900111935.1	2016/11/02	2017/07/29	DOE - JOINT GENOME INSTITUTE
GCF_010119195.1	2020/02/03	2020/02/10	Anhui Normal University
GCF_003259835.1	2018/06/21	2018/06/23	DOE Joint Genome Institute
GCF_002954685.1	2018/02/27	2018/03/04	Atmosphere and Ocean Research Institute, The University of Tokyo
GCF_003185915.1	2018/06/04	2018/06/07	Institute of Microbiology, Chinese Academy of Sciences
GCF_004216855.1	2019/02/20	2019/02/22	DOE Joint Genome Institute
GCF_003797885.1	2018/11/19	2018/11/26	Korea Polar Research Institute

GCF_900143245.1	2016/12/03	2016/12/18	DOE - JOINT GENOME INSTITUTE
GCF_003312425.1	2018/07/10	2018/07/15	Konggi University
GCF_002954665.1	2018/02/27	2018/03/04	Atmosphere and Ocean Research Institute, The University of Tokyo
GCF_009791395.1	2019/12/22	2020/01/02	CSIR-IMTECH
GCF_003219795.1	2018/06/12	2018/06/16	Institute of Microbiology, Chinese Academy of Sciences
GCF_002836945.1	2017/12/12	2017/12/14	National Institute for Communicable Disease Control and Prevention, China CDC
GCF_900102145.1	2016/10/21	2017/07/29	DOE - JOINT GENOME INSTITUTE
GCF_001402945.1	2015/10/22	2015/10/23	RIPCM
GCF_000383975.1	2013/04/26	2014/07/11	JGI
GCF_000337795.1	2013/02/04	2013/02/07	University of California, Davis
GCF_000337815.1	2013/02/04	2013/02/07	University of California, Davis
GCF_900176435.1	2017/04/08	2021/06/08	DOE - JOINT GENOME INSTITUTE
GCF_900111075.1	2016/10/31	2016/12/22	DOE - JOINT GENOME INSTITUTE
GCF_900108085.1	2016/10/24	2018/02/10	DOE - JOINT GENOME INSTITUTE
GCF_000368025.1	2013/04/19	2013/05/09	Broad Institute
GCF_900175965.1	2017/04/07	2017/04/17	DOE - JOINT GENOME INSTITUTE
GCF_000423905.1	2013/07/11	2014/07/12	DOE Joint Genome Institute
GCF_000337915.1	2013/02/04	2013/02/07	University of California, Davis
GCF_000337555.1	2013/02/04	2013/02/07	University of California, Davis
GCF_000298075.1	2012/09/21	2014/07/12	URMITE
GCF_000688455.1	2014/04/08	2014/07/11	DOE Joint Genome Institute
GCF_007997305.1	2019/08/14	2019/08/17	University of Tasmania
GCF_004217335.1	2019/02/20	2019/02/22	DOE Joint Genome Institute
GCF_008369445.1	2019/09/10	2019/09/16	Chung-Ang University
GCF_004216575.1	2019/02/20	2019/02/22	DOE Joint Genome Institute
GCF_001571405.1	2016/01/19	2016/08/18	Bioproduction Research Institute, National Institute of Advanced Industrial Science and Technology (AIST)
GCF_003350545.1	2018/07/31	2018/08/02	DOE Joint Genome Institute
GCF_003148585.1	2018/05/22	2018/05/26	DOE Joint Genome Institute

GCF_900116805.1	2016/11/02	2016/12/23	DOE - JOINT GENOME INSTITUTE
GCF_008245225.1	2019/09/03	2019/09/16	NCMR- NCCS
GCF_007988865.1	2019/08/01	2019/08/16	National Institute of Technology and Evaluation
GCF_000934755.1	2015/02/26	2015/06/09	DOE Joint Genome Institute
GCF_001950255.1	2016/12/09	2017/01/16	Laboratory of Marine Microbiology, Graduate School of Agriculture, Kyoto University
GCF_000504205.1	2013/12/05	2013/12/10	JGI
GCF_000744825.1	2014/08/28	2014/12/23	DOE Joint Genome Institute
GCF_000364845.1	2013/04/15	2013/05/09	First Institute of Oceanography, State Oceanic Administration of China
GCF_000220175.1	2011/07/07	2016/11/30	Korea Research Institute of Bioscience and Biotechnology
GCF_000183545.2	2012/11/05	2016/04/19	US DOE Joint Genome Institute (JGI-PGF)
GCF_000258515.1	2012/04/11	2014/07/12	Korea Research Institute of Bioscience and Biotechnology
GCF_000153225.1	2006/05/04	2016/11/29	The Gordon and Betty Moore Foundation Marine Microbiology Initiative
GCF_000378345.1	2013/04/23	2014/07/11	DOE Joint Genome Institute
GCF_000419685.1	2013/07/09	2014/07/11	DOE Joint Genome Institute
GCF_000379085.1	2013/04/23	2014/07/11	JGI
GCF_000336755.1	2013/02/04	2013/02/07	University of California, Davis
GCF_000347775.1	2013/03/21	2013/03/29	University of Manchester
GCF_000373205.1	2013/04/23	2014/07/11	DOE Joint Genome Institute
GCF_000425505.1	2013/07/11	2014/07/11	DOE Joint Genome Institute
GCF_000336615.1	2013/02/04	2013/02/07	University of California, Davis
GCF_000421625.1	2013/07/09	2014/07/12	DOE Joint Genome Institute
GCF_000337675.1	2013/02/04	2013/02/07	University of California, Davis
GCF_000518605.1	2014/01/13	2014/07/11	DOE Joint Genome Institute
GCF_000966265.1	2015/03/30	2015/06/26	DOE Joint Genome Institute
GCF_000336995.1	2013/02/04	2013/02/07	University of California, Davis
GCF_000472905.1	2013/09/30	2014/07/11	DOE Joint Genome Institute
GCF_000471625.1	2013/09/24	2013/09/26	Centre for Cellular and Molecular Biology
GCF_001598235.1	2016/03/11	2016/08/21	National Institute of Technology and Evaluation
GCF_000337735.1	2013/02/04	2013/02/07	University of California, Davis

GCF_000276805.1	2012/07/09	2014/07/11	Korea Polar Research Institute
GCF_000337575.1	2013/02/04	2013/02/07	University of California, Davis
GCF_000425485.1	2013/07/11	2014/07/11	DOE Joint Genome Institute
GCF_000423105.1	2013/07/11	2014/07/11	DOE Joint Genome Institute
GCF_000337375.1	2013/02/04	2013/02/07	University of California, Davis
GCF_000430585.1	2013/07/16	2014/07/11	DOE Joint Genome Institute
GCF_000378465.1	2013/04/23	2014/07/11	DOE Joint Genome Institute
GCF_000428725.1	2013/07/15	2014/07/11	DOE Joint Genome Institute
GCF_001552655.1	2016/02/06	2016/08/19	National Institute of Technology and Evaluation
GCF_000337135.1	2013/02/04	2013/02/07	University of California, Davis
GCF_000337475.1	2013/02/04	2013/02/07	University of California, Davis
GCF_000336935.1	2013/02/04	2013/02/07	University of California, Davis
GCF_000518805.1	2014/01/13	2014/07/11	DOE Joint Genome Institute
GCF_000337035.1	2013/02/04	2013/02/07	University of California, Davis
GCF_000337695.1	2013/02/04	2013/02/07	University of California, Davis
GCF_000376225.1	2013/04/20	2014/07/11	DOE Joint Genome Institute
GCF_000381745.1	2013/04/24	2014/07/11	DOE Joint Genome Institute
GCF_001552255.1	2016/02/03	2016/08/19	National Institute of Technology and Evaluation
GCF_000482725.1	2013/10/25	2014/07/11	DOE Joint Genome Institute
GCF_000429525.1	2013/07/15	2014/07/11	DOE Joint Genome Institute
GCF_000376265.1	2013/04/20	2014/07/12	DOE Joint Genome Institute
GCF_001544355.1	2016/01/29	2016/08/18	National Institute of Technology and Evaluation
GCF_000429345.1	2013/09/05	2014/07/11	DOE Joint Genome Institute
GCF_000430045.1	2013/07/15	2014/07/11	JGI
GCF_001552675.1	2016/02/06	2016/03/28	National Institute of Technology and Evaluation
GCF_000423425.1	2013/07/11	2014/07/11	DOE Joint Genome Institute
GCF_000243455.1	2012/01/23	2016/11/30	JGI
GCF_000420245.1	2013/07/09	2014/07/11	DOE Joint Genome Institute
GCF_004365915.1	2019/03/22	2019/03/27	DOE Joint Genome Institute
GCF_001953745.1	2017/01/18	2017/01/20	National Chung Hsing University

GCF_001542905.1	2016/01/29	2016/08/18	Silla university
GCF_000955725.1	2015/03/17	2015/03/19	DOE Joint Genome Institute
GCF_900142385.1	2016/12/03	2016/12/18	DOE - JOINT GENOME INSTITUTE
GCF_017873855.1	2021/04/08	2021/04/10	DOE Joint Genome Institute
GCF_009729545.1	2019/12/04	2019/12/09	North Carolina State University
GCF_000955745.1	2015/03/17	2015/03/19	DOE Joint Genome Institute
GCF_000744885.1	2014/08/28	2014/12/25	DOE Joint Genome Institute
GCF_003634755.1	2018/10/11	2018/10/18	DOE Joint Genome Institute
GCF_001462395.1	2015/12/08	2015/12/10	Oak Ridge National Laboratory
GCF_900095845.1	2016/09/08	2017/01/11	URMITE
GCF_900114435.1	2016/11/02	2017/07/29	DOE - JOINT GENOME INSTITUTE
GCF_000955735.1	2015/03/17	2015/03/19	DOE Joint Genome Institute
GCF_900116205.1	2016/11/02	2017/07/29	DOE - JOINT GENOME INSTITUTE
GCF_900104065.1	2016/10/21	2017/07/29	DOE - JOINT GENOME INSTITUTE
GCF_007671655.1	2019/07/30	2019/08/06	Third Institute of State Oceanic Administration
GCF_007671675.1	2019/07/30	2019/08/06	Third Institute of State Oceanic Administration
GCF_900112175.1	2016/11/02	2017/07/29	DOE - JOINT GENOME INSTITUTE
GCF_003226325.1	2018/06/13	2018/06/16	Chinese Academy of Agricultural Sciences
GCF_900103715.1	2016/10/21	2017/07/29	DOE - JOINT GENOME INSTITUTE
GCF_900108095.1	2016/10/27	2017/07/29	DOE - JOINT GENOME INSTITUTE
GCF_900215575.1	2017/09/28	2017/10/12	DOE - JOINT GENOME INSTITUTE
GCF_003688495.1	2018/10/25	2018/10/27	DOE Joint Genome Institute
GCF_014647455.2	2020/09/11	2020/10/31	WFCC-MIRCEN World Data Centre for Microorganisms (WDCM)
GCF_900110865.1	2016/10/31	2017/07/29	DOE - JOINT GENOME INSTITUTE
GCF_017874375.1	2021/04/08	2021/04/10	DOE Joint Genome Institute
GCF_007004735.1	2019/07/12	2019/07/18	Jiangsu Universiry
GCF_001748385.1	2016/07/28	2016/09/29	Korea Basic Science Institute
GCF_000455345.1	2013/08/20	2015/08/02	ebi
GCF_004102725.1	2019/01/22	2019/01/26	National Chung Hsing University
GCF_900166965.1	2017/03/17	2017/03/24	UVEG

GCF_001602375.1	2016/01/19	2016/08/18	Bioproduction Research Institute, National Institute of Advanced Industrial Science and Technology (AIST)
GCF_001375655.1	2015/04/22	2017/07/29	URMITE
GCF_002806945.1	2017/12/04	2017/12/06	Shandong Uniwersity at Weihai
GCF_014647115.1	2020/09/11	2020/09/20	WFCC-MIRCEN World Data Centre for Microorganisms (WDCM)
GCF_002153915.1	2017/05/22	2017/05/24	National Center for Biotechnology Information
GCF_015223105.1	2020/11/03	2020/11/05	Guangdong Academy of Sciences
GCF_002954225.1	2018/02/27	2018/03/04	Institute of Microbiology, Chinese Academy of Sciences
GCF_003966225.1	2018/12/23	2018/12/28	Third Institute of Oceanography, State of Oceanic Administration
GCF_014647155.1	2020/09/11	2020/09/20	WFCC-MIRCEN World Data Centre for Microorganisms (WDCM)
GCF_900129575.1	2016/12/03	2016/12/18	DOE - JOINT GENOME INSTITUTE
GCF_001758465.1	2016/10/12	2016/10/13	Second Institute of Oceanography, State Oceanic Administration
GCF_017873335.1	2021/04/08	2021/04/10	DOE Joint Genome Institute
GCF_900114835.1	2016/11/02	2017/07/29	DOE - JOINT GENOME INSTITUTE
GCF_004765815.2	2020/01/27	2020/02/07	Jiangsu University
GCF_003719725.1	2018/11/10	2018/11/14	Northwest Institute of Eco-Environment and Resources,CAS
GCF_900106945.1	2016/10/22	2017/07/29	DOE - JOINT GENOME INSTITUTE
GCF_000711215.1	2014/06/24	2015/02/16	DOE Joint Genome Institute
GCF_003217155.1	2018/06/12	2018/06/16	DOE Joint Genome Institute
GCF_003966265.1	2018/12/23	2018/12/28	Third Institute of Oceanography, State of Oceanic Administration
GCF_900110375.1	2016/10/31	2017/07/29	DOE - JOINT GENOME INSTITUTE
GCF_004137855.1	2019/02/05	2019/02/16	UBO
GCF_000766865.1	2014/10/10	2014/12/24	Cornell University
GCF_008122205.1	2019/08/28	2019/09/03	NCMR- NCCS
GCF_013760845.1	2020/07/27	2020/08/03	DOE Joint Genome Institute
GCF_000687875.1	2014/04/08	2014/07/11	DOE Joint Genome Institute
GCF_001029285.1	2015/06/17	2015/06/18	Goettingen Genomics Laboratory
GCF_900107635.1	2016/10/22	2017/07/29	DOE - JOINT GENOME INSTITUTE
GCF_900113635.1	2016/11/02	2017/07/29	DOE - JOINT GENOME INSTITUTE
GCF_900142415.1	2016/12/03	2016/12/18	DOE - JOINT GENOME INSTITUTE

GCF_000934745.1	2015/02/26	2015/03/19	DOE Joint Genome Institute
GCF_900182635.1	2019/07/11	2019/07/20	DOE - JOINT GENOME INSTITUTE
GCF_000686525.1	2014/05/08	2014/07/11	DOE Joint Genome Institute
GCF_002909375.1	2018/01/30	2018/02/01	Institute of Microbiology, Chinese Academy of Sciences
GCF_900142885.1	2016/12/03	2016/12/18	DOE - JOINT GENOME INSTITUTE
GCF_003026475.1	2018/03/31	2018/04/05	FDA/CFSAN
GCF_000753795.1	2014/09/10	2014/12/24	85303
GCF_002094855.1	2017/04/17	2017/04/21	The Third Institute of State Oceanic Administration (SOA)
GCF_900108015.1	2016/10/24	2018/02/11	DOE - JOINT GENOME INSTITUTE
GCF_001017125.1	2015/06/01	2015/06/26	National Chung Hsing University
GCF_004745425.1	2019/04/08	2019/04/11	National Chung Hsing University
GCF_017873625.1	2021/04/08	2021/04/10	DOE Joint Genome Institute
GCF_001640115.1	2016/05/06	2016/08/19	Korea University
GCF_900129585.1	2016/12/03	2016/12/18	DOE - JOINT GENOME INSTITUTE
GCF_900106645.1	2016/10/22	2017/07/29	DOE - JOINT GENOME INSTITUTE
GCF_002954245.1	2018/02/27	2018/03/04	Institute of Microbiology, Chinese Academy of Sciences
GCF_900156755.1	2017/01/14	2017/01/21	DOE - JOINT GENOME INSTITUTE
GCF_014201585.1	2020/08/14	2020/08/17	DOE Joint Genome Institute
GCF_001594015.1	2016/03/18	2016/03/28	Goettingen Genomics Laboratory
GCF_900129115.1	2016/12/03	2016/12/18	DOE - JOINT GENOME INSTITUTE
GCF_003336745.1	2018/07/24	2018/07/26	SUN YAT-SEN UNIVERSITY
GCF_900104215.1	2016/10/21	2017/07/29	DOE - JOINT GENOME INSTITUTE
GCF_001029445.1	2015/06/18	2015/06/19	Technical University of Denmark
GCF_014201825.1	2020/08/14	2020/08/17	DOE Joint Genome Institute
GCF_001939735.1	2017/01/09	2017/01/12	Yantai Institute of Coastal Zone Research,Chinese Academy of Sciences
GCF_003841505.1	2018/11/26	2018/11/30	Winogradsky Institute of Microbiology
GCF_900099915.1	2016/10/21	2017/07/29	DOE - JOINT GENOME INSTITUTE
GCF_900112975.1	2016/11/02	2017/07/29	DOE - JOINT GENOME INSTITUTE
GCF_004366795.1	2019/03/22	2019/03/27	DOE Joint Genome Institute
GCF_900111555.1	2016/10/31	2017/07/29	DOE - JOINT GENOME INSTITUTE

GCF_900188065.1	2017/07/16	2017/07/21	DOE - JOINT GENOME INSTITUTE
GCF_900110465.1	2016/11/17	2017/07/29	DOE - JOINT GENOME INSTITUTE
GCF_001719065.1	2016/09/02	2016/09/07	IRNAS-CSIC
GCF_003219815.1	2018/06/12	2018/06/16	Institute of Microbiology, Chinese Academy of Sciences
GCF_900112455.1	2016/11/02	2017/07/29	DOE - JOINT GENOME INSTITUTE
GCF_900128885.1	2016/12/03	2016/12/18	DOE - JOINT GENOME INSTITUTE
GCF_000614975.1	2014/03/31	2014/07/11	The University of Tokyo
GCF_003317055.1	2018/07/12	2018/07/18	DOE Joint Genome Institute
GCF_007197645.1	2019/07/20	2019/08/01	Third Institute of Oceanography, State of Oceanic Administration
GCF_003025615.1	2018/03/31	2018/04/05	FDA/CFSAN
GCF_003862495.1	2018/12/04	2018/12/08	institute of microbial technology
GCF_003173335.1	2018/05/28	2018/05/31	Aalborg University
GCF_000525995.1	2014/01/31	2014/12/24	Cornell University
GCF_001399675.1	2015/10/19	2015/10/21	California Institute of Technology
GCF_900142255.1	2016/12/03	2016/12/18	DOE - JOINT GENOME INSTITUTE
GCF_003731635.1	2018/11/15	2018/11/21	Zhejiang Sci-Tech University
GCF_900156265.1	2017/01/14	2021/06/08	DOE - JOINT GENOME INSTITUTE
GCF_000620065.1	2014/04/08	2014/07/11	DOE Joint Genome Institute
GCF_000711975.1	2014/06/25	2014/08/05	DOE Joint Genome Institute
GCF_002995805.1	2018/03/14	2018/03/16	Goettingen Genomics Laboratory
GCF_016107535.1	2020/12/17	2020/12/19	INRAE
GCF_002995755.1	2018/03/14	2018/03/16	Goettingen Genomics Laboratory
GCF_004923255.1	2019/04/29	2019/05/03	GEOMAR - Helmholtz Centre for Ocean Research Kiel
GCF_003368535.1	2018/08/09	2018/08/15	Winogradsky Institute of Microbiology
GCF_000525875.1	2014/01/31	2014/12/24	Cornell University
GCF_000615945.1	2014/03/31	2014/07/11	The University of Tokyo
GCF_900142695.1	2016/12/03	2016/12/18	DOE - JOINT GENOME INSTITUTE
GCF_002243045.1	2017/08/07	2017/08/09	National Chung Hsing University
GCF_900111865.1	2016/11/02	2017/07/29	DOE - JOINT GENOME INSTITUTE
GCF_000784355.1	2014/11/17	2015/06/26	Leibniz Institute DSMZ Braunschweig

GCF_001484195.1	2016/01/04	2016/01/06	Kyungpook National University, Shin's Lab.
GCF_900128965.1	2016/12/03	2016/12/18	DOE - JOINT GENOME INSTITUTE
GCF_002964845.1	2018/02/28	2018/03/12	SUN YAT-SEN UNIVERSITY
GCF_900111795.1	2016/11/02	2017/07/29	DOE - JOINT GENOME INSTITUTE
GCF_900109085.1	2016/10/31	2017/07/29	DOE - JOINT GENOME INSTITUTE
GCF_000949295.1	2015/03/06	2015/08/19	Georg-August-University Goettingen, Genomic and Applied Microbiology, Goettingen Genomics Laboratory
GCF_003574095.1	2018/09/18	2018/09/20	Biocant
GCF_900101165.1	2016/10/21	2017/07/29	DOE - JOINT GENOME INSTITUTE
GCF_002287235.1	2017/09/06	2017/09/08	University of California Santa Barbara
GCF_003574085.1	2018/09/18	2018/09/20	Biocant
GCF_902143385.2	2019/09/17	2019/09/21	RADBOUD UNIVERSITY NIJMEGEN
GCF_009617755.1	2019/10/25	2019/11/12	National Institute of Technology and Evaluation (NITE)
GCF_001447355.1	2015/11/23	2015/11/25	Yunnan normal university
GCF_000243315.1	2012/01/23	2016/11/30	JGI
GCF_000745065.1	2014/08/28	2014/12/25	DOE Joint Genome Institute
GCF_904846075.1	2020/11/21	2021/01/22	MAX PLANCK INSTITUTE FOR DEVELOPMENTAL BIOLOGY
GCF_003350005.1	2018/07/31	2018/08/02	University of Helsinki
GCF_000709345.1	2014/06/17	2014/07/11	China Center for Type Culture Collection
GCF_000632495.1	2014/04/15	2015/06/26	INDEAR
GCF_001950325.1	2016/12/09	2017/01/16	Laboratory of Marine Microbiology, Graduate School of Agriculture, Kyoto University
GCF_001444505.1	2015/11/17	2015/11/19	Third Institute of Oceanography, State Oceanic Administration
GCF_900162825.1	2017/02/10	2017/02/20	INSTITUT PASTEUR
GCF_001027105.1	2015/06/03	2015/06/22	Okinawa Institute of Advanced Sciences
GCF_009759685.1	2019/12/17	2019/12/19	Monash University
GCF_000742895.1	2014/08/21	2015/01/21	Los Alamos National Laboratory
GCF_006094395.1	2019/06/06	2019/06/08	Zhejiang Tianke High Technology Development Co.Ltd.
GCF_000982825.1	2015/04/30	2015/05/06	Food-borne Pathogen Omics Research Center
GCF_900636385.1	2018/12/20	2018/12/28	SC

GCF_000021485.1	2008/12/19	2016/11/29	TIGR
GCF_009183365.2	2021/03/24	2021/03/26	Karolinska Institutet
GCF_000010125.1	2005/08/12	2016/11/29	Kitasato Institute for Life Sciences, Japan
GCF_000026225.1	2008/12/18	2016/11/29	Genoscope
GCF_000022485.1	2009/04/29	2016/11/29	US DOE Joint Genome Institute (JGI-PGF)
GCF_900079115.1	2016/04/11	2016/04/29	Institute of Biology
GCF_006757745.1	2019/06/14	2019/07/12	National Institute of Technology and Evaluation
GCF_016406325.1	2020/12/27	2020/12/28	Ocean University of China
GCF_000012285.1	2005/07/05	2016/11/29	Danish Archaea Centre
GCF_001295365.1	2015/09/22	2016/01/27	Institute of Microbiology, Chinese Academy of Sciences
GCF_900475595.1	2018/06/17	2018/06/25	SC
GCF_000007305.1	2002/02/27	2016/11/29	Utah Genome Center
GCF_000970205.1	2015/04/07	2015/06/25	University of Illinois at Urbana-Champaign
GCF_900187025.1	2017/08/15	2017/08/28	SC
GCF_000008545.1	2001/01/09	2020/03/06	TIGR
GCF_000175575.2	2014/05/27	2022/02/12	Center for Bioinformatics and Genome Biology
GCF_000145615.1	2010/08/13	2016/11/29	US DOE Joint Genome Institute (JGI-PGF)
GCF_002900365.1	2018/01/26	2018/01/31	Institute of Microbiology Chinese Academy of Sciences
GCF_001267405.1	2015/08/13	2015/08/22	Goettingen Genomics Laboratory
GCF_000306765.2	2014/05/27	2015/03/09	Institute of Microbiology, Chinese Academy of Sciences
GCF_001543205.1	2016/02/01	2016/02/02	Slagelse Hospital
GCF_900636325.1	2018/12/20	2018/12/28	SC
GCF_000816145.1	2015/01/07	2016/05/29	University of Waterloo
GCF_000008665.1	1999/12/22	2016/11/29	TIGR
GCF_000008645.1	1999/12/22	2016/11/29	Genome Therapeutics Corporation
GCF_004799665.1	2019/04/18	2019/04/24	Wuhan University
GCF_003967175.1	2018/08/01	2018/12/28	Laboratory of Extremophiles, Environmental Engineering for Symbiosis, Graduate school Soka University
GCF_002214545.1	2017/07/05	2017/07/07	Ecole Normale Superieure de Lyon
GCF_000819445.1	2015/01/16	2015/01/24	CeBiTec, Bielefeld University

GCF_000020585.3	2013/09/13	2017/07/10	The Gordon and Betty Moore Foundation Marine Microbiology Initiative
GCF_000013905.1	2006/04/18	2016/11/29	DOE Joint Genome Institute
GCF_000060285.1	2006/10/27	2017/02/26	Universitat Giessen
GCF_000446015.1	2013/08/13	2015/08/20	Baltic Federal University
GCF_003019275.1	2018/03/26	2018/03/29	Illinois Institute of Technology
GCF_002214605.1	2017/07/05	2017/07/07	Ecole Normale Superieure de Lyon
GCF_013343115.1	2020/06/15	2020/06/15	US Food and Drug Administration
GCF_016406305.1	2020/12/27	2020/12/28	Ocean University of China
GCF_001445575.1	2015/11/18	2016/03/28	CSIR-Institute of Himalayan Bioresource Technology
GCF_001294625.1	2015/09/18	2016/03/28	University of Malaya
GCF_000219875.1	2011/07/05	2016/11/30	Key Laboratory of Genome Science and Information, Beijing Institute of Genomics, Chinese Academy of Sciences
GCF_001644665.1	2016/05/16	2016/05/17	University of Manitoba
GCF_000012745.1	2005/09/01	2016/11/29	DOE Joint Genome Institute
GCF_001679725.1	2016/07/05	2016/08/18	Korea Polar Research Institute
GCF_002355395.1	2016/01/15	2017/09/27	Graduate School of Agriculture, Kindai University
GCF_001880325.1	2016/11/17	2016/11/18	Hamburg University of Technology
GCF_000828575.1	2012/12/03	2015/06/26	National Institute of Genetics
GCF_019263745.1	2021/07/16	2021/07/18	University of Minnesota
GCF_000025665.1	2010/02/24	2016/11/29	US DOE Joint Genome Institute
GCF_002966495.1	2018/03/01	2018/03/07	Wuhan University
GCF_004634385.1	2019/04/04	2019/04/06	Chungbuk national university
GCF_001719125.1	2016/09/06	2016/09/07	Institute of Microbiology, Chinese Academy of Sciences
GCF_000195575.1	2011/04/08	2016/11/29	Georg-August-University Goettingen
GCF_017310505.1	2021/03/09	2021/03/13	Korea Institute of Geoscience and Mineral Resources
GCF_000151205.2	2011/09/08	2018/04/04	Moore Foundation
GCF_000211475.1	2011/04/29	2016/11/29	Korea Ocean Research and Development Institute
GCF_013282665.1	2020/06/06	2020/06/07	PulseNet Next Generation Subtyping Methods Unit
GCF_002201895.1	2017/06/20	2019/09/01	Zhejiang University of Technology
GCF_000148995.1	2010/11/05	2016/11/29	Baylor College of Medicine

GCF_001544255.1	2016/01/28	2016/02/03	National Institute of Technology and Evaluation
GCF_000507185.2	2015/07/20	2015/08/14	KRIBB
GCF_001792775.2	2018/06/30	2018/07/13	University of Tennessee
GCF_002901845.1	2018/01/27	2018/01/31	Modernising Medical Microbiology
GCF_002901825.1	2018/01/27	2018/01/31	Modernising Medical Microbiology
GCF_002732165.1	2017/10/26	2017/10/28	Masaryk University
GCF_008124605.1	2019/08/29	2019/09/03	DOE Joint Genome Institute
GCF_002902325.1	2018/01/27	2018/01/31	Modernising Medical Microbiology
GCF_000759775.1	2014/09/25	2015/02/09	National Institute of Technology and Evaluation (NITE)
GCF_002901705.1	2018/01/27	2018/01/31	Modernising Medical Microbiology
GCF_002217405.1	2017/07/12	2017/07/14	FDA
GCF_003026815.1	2018/03/31	2018/04/05	FDA/CFSAN
GCF_003970495.1	2018/12/24	2021/07/17	Modernising Medical Microbiology
GCF_900456975.1	2018/08/01	2018/08/07	SC
GCF_000369065.1	2013/04/19	2013/05/09	Broad Institute
GCF_900458565.1	2018/08/01	2018/08/04	SC
GCF_014647135.1	2020/09/12	2020/09/20	WFCC-MIRCEN World Data Centre for Microorganisms (WDCM)
GCF_004345675.1	2019/03/11	2019/03/16	DOE Joint Genome Institute
GCF_000875895.1	2015/02/13	2015/02/17	Zhejiang University
GCF_002901865.1	2018/01/27	2018/01/31	Modernising Medical Microbiology
GCF_002902755.1	2018/01/27	2018/01/31	Modernising Medical Microbiology
GCF_002902345.1	2018/01/27	2018/01/31	Modernising Medical Microbiology
GCF_900105965.1	2016/10/22	2016/12/22	DOE - JOINT GENOME INSTITUTE
GCF_002902285.1	2018/01/27	2018/01/31	Modernising Medical Microbiology
GCF_002902235.1	2018/01/27	2018/01/31	Modernising Medical Microbiology
GCF_002902725.1	2018/01/27	2018/01/31	Modernising Medical Microbiology
GCF_002901945.1	2018/01/27	2018/01/31	Modernising Medical Microbiology
GCF_001280255.1	2015/09/08	2015/09/11	University of Gdansk
GCF_002072215.1	2017/03/24	2017/03/28	Goettingen Genomics Laboratory
GCF_000381045.1	2013/04/24	2014/07/12	DOE Joint Genome Institute

GCF_000368825.1	2013/04/19	2013/05/09	Broad Institute
GCF_000444055.1	2013/08/05	2014/07/17	Agricultural Research Organisation
GCF_014635045.1	2020/09/11	2020/09/19	WFCC-MIRCEN World Data Centre for Microorganisms (WDCM)
GCF_001500315.1	2016/01/06	2016/01/08	Harbin Medical University
GCF_000766145.1	2014/10/10	2014/12/24	Cornell University
GCF_003970575.1	2018/12/24	2021/07/17	Modernising Medical Microbiology
GCF_003797125.1	2018/11/19	2018/11/26	Korea Polar Research Institute
GCF_002902685.1	2018/01/27	2018/01/31	Modernising Medical Microbiology
GCF_000337775.1	2013/02/04	2013/02/07	University of California, Davis
GCF_014646555.1	2020/09/11	2020/09/20	WFCC-MIRCEN World Data Centre for Microorganisms (WDCM)
GCF_000632715.1	2014/04/11	2015/02/09	National Institute of Technology and Evaluation
GCF_000188295.1	2011/02/10	2016/11/29	Baylor College of Medicine
GCF_014200405.1	2020/08/14	2020/08/17	DOE Joint Genome Institute
GCF_003025595.1	2018/03/31	2018/04/05	FDA/CFSAN
GCF_004402405.1	2019/03/26	2019/03/29	Institute of Microbiology, Chinese Academy of Sciences
GCF_900156405.1	2017/01/14	2017/01/21	DOE - JOINT GENOME INSTITUTE
GCF_900109325.1	2016/10/31	2017/07/29	DOE - JOINT GENOME INSTITUTE
GCF_002836975.1	2017/12/12	2017/12/14	National Institute for Communicable Disease Control and Prevention, China CDC
GCF_000833605.1	2015/02/09	2015/02/10	Universiti Teknologi Malaysia
GCF_900109825.1	2016/10/31	2017/07/29	DOE - JOINT GENOME INSTITUTE
GCF_000820505.1	2015/01/16	2015/01/21	University of Minnesota
GCF_001310975.1	2015/10/01	2015/10/15	The University of Tokyo
GCF_001639275.1	2016/04/29	2016/05/13	Goettingen Genomics Laboratory
GCF_003574035.1	2018/09/18	2018/09/20	Biocant
GCF_002902265.1	2018/01/27	2018/01/31	Modernising Medical Microbiology
GCF_000188055.2	2011/11/18	2016/11/30	JCVI
GCF_000513855.1	2014/01/07	2015/02/14	DOE Joint Genome Institute
GCF_001006765.1	2015/05/14	2015/05/15	Jiamusi University
GCF_000425165.1	2013/07/11	2014/07/11	DOE Joint Genome Institute

GCF_000373145.1	2013/04/23	2014/07/12	DOE Joint Genome Institute
GCF_000421725.1	2013/07/09	2014/07/11	DOE Joint Genome Institute
GCF_001570745.1	2016/02/10	2016/08/18	National Institute of Technology and Evaluation
GCF_000525975.1	2014/01/31	2014/12/24	Cornell University
GCF_000344175.1	2013/03/04	2013/03/29	Cornell University
GCF_900101115.1	2016/10/21	2017/07/29	DOE - JOINT GENOME INSTITUTE
GCF_000934465.1	2015/02/25	2015/03/04	Zhejiang University
GCF_000518705.1	2014/01/13	2014/07/11	DOE Joint Genome Institute
GCF_000382145.1	2013/04/24	2014/07/11	DOE Joint Genome Institute
GCF_002902385.1	2018/01/27	2018/01/31	Modernising Medical Microbiology
GCF_003026395.1	2018/03/31	2018/04/05	FDA/CFSAN
GCF_002777975.1	2017/11/16	2017/11/22	National Institute for Communicable Disease Control and Prevention, China CDC
GCF_900103805.1	2016/10/21	2017/07/29	DOE - JOINT GENOME INSTITUTE
GCF_002909445.1	2018/01/30	2018/02/01	Institute of Microbiology, Chinese Academy of Sciences
GCF_003605125.1	2018/10/01	2018/10/04	National Institute for Communicable Disease Control and Prevention, China CDC
GCF_003026355.1	2018/03/31	2018/04/05	FDA/CFSAN
GCF_014651955.1	2020/09/12	2020/09/21	WFCC-MIRCEN World Data Centre for Microorganisms (WDCM)
GCF_001077885.1	2015/07/14	2015/07/16	CIAD, A.C.
GCF_900110125.1	2016/10/31	2017/07/29	DOE - JOINT GENOME INSTITUTE
GCF_000525795.1	2014/01/31	2014/12/24	Cornell University
GCF_002954455.1	2018/02/27	2018/03/04	Atmosphere and Ocean Research Institute, The University of Tokyo
GCF_002849605.1	2018/01/02	2018/01/11	Technische Universitaet Muenchen
GCF_900166975.1	2017/03/17	2017/03/24	UVEG
GCF_002902305.1	2018/01/27	2018/01/31	Modernising Medical Microbiology
GCF_002902405.1	2018/01/27	2018/01/31	Modernising Medical Microbiology
GCF_002902625.1	2018/01/27	2018/01/31	Modernising Medical Microbiology
GCF_000338275.1	2013/02/07	2014/07/12	Zhejiang University
GCF_018854495.1	2021/06/11	2021/06/13	Fundacion Ciencia y Vida

GCF_015276835.1	2020/11/06	2020/11/10	Cornell University
GCF_002994445.1	2018/03/12	2018/03/16	Helsinki University
GCF_003043455.1	2018/04/06	2018/04/18	University of Calgary
GCF_000513315.1	2013/12/19	2015/02/16	URMITE
GCF_007991715.1	2019/07/31	2019/08/16	National Institute of Technology and Evaluation Biological Resource Center
GCF_001280565.1	2015/09/09	2015/09/21	Fundacion Ciencia & Vida
GCF_003026285.1	2018/03/31	2018/04/05	FDA/CFSAN
GCF_003399645.1	2018/08/16	2018/08/21	Korea Polar Research Institute
GCF_003399625.1	2018/08/16	2018/08/21	Korea Polar Research Institute
GCF_003264935.1	2018/06/25	2018/07/12	Agharkar Research Institute
GCF_003399635.1	2018/08/16	2018/08/21	Korea Polar Research Institute
GCF_900095385.1	2016/12/16	2016/12/22	CEBITEC
GCF_000744175.1	2014/08/28	2014/12/25	DOE Joint Genome Institute
GCF_000252125.1	2012/03/08	2016/11/30	University of Nevada Las Vegas
GCF_904846175.1	2020/11/21	2021/01/22	MAX PLANCK INSTITUTE FOR DEVELOPMENTAL BIOLOGY
GCF_904846235.1	2020/11/21	2021/01/22	MAX PLANCK INSTITUTE FOR DEVELOPMENTAL BIOLOGY
GCF_900215215.1	2017/09/21	2017/09/29	DOE - JOINT GENOME INSTITUTE
GCF_904846285.1	2020/11/21	2021/01/22	MAX PLANCK INSTITUTE FOR DEVELOPMENTAL BIOLOGY
GCF_900111485.1	2016/11/17	2017/07/29	DOE - JOINT GENOME INSTITUTE
GCF_904846225.1	2020/11/21	2021/01/22	MAX PLANCK INSTITUTE FOR DEVELOPMENTAL BIOLOGY
GCF_904846185.1	2020/11/21	2021/01/22	MAX PLANCK INSTITUTE FOR DEVELOPMENTAL BIOLOGY
GCF_000702485.1	2014/06/11	2014/07/11	DOE Joint Genome Institute
GCF_002572525.1	2017/10/17	2017/10/20	World Institute of Kimchi
GCF_902712915.1	2020/03/05	2020/06/14	Friedrich Schiller University Jena
GCF_018860765.1	2021/06/12	2021/06/14	Massachusetts Institute of Technology
GCF_003841465.1	2018/11/26	2018/11/30	Winogradsky Institute of Microbiology
GCF_018861005.1	2021/06/12	2021/06/14	Massachusetts Institute of Technology
GCF_003722075.1	2018/11/12	2018/11/14	King Abdullah University of Science and Technology (KAUST)
GCF_003097355.1	2018/05/07	2018/08/29	Florida A&M University
GCF_904846695.1	2020/11/21	2021/01/22	MAX PLANCK INSTITUTE FOR DEVELOPMENTAL BIOLOGY

GCF_904846215.1	2020/11/21	2021/01/22	MAX PLANCK INSTITUTE FOR DEVELOPMENTAL BIOLOGY
GCF_904846675.1	2020/11/21	2021/01/22	MAX PLANCK INSTITUTE FOR DEVELOPMENTAL BIOLOGY
GCF_904846415.1	2020/11/21	2021/01/22	MAX PLANCK INSTITUTE FOR DEVELOPMENTAL BIOLOGY
GCF_003035445.1	2018/04/05	2018/04/12	University of Calgary
GCF_001707885.1	2016/08/18	2016/08/21	Institute of Bioengineering, Research Center of Biotechnology of RAS
GCF_009765975.1	2019/12/18	2020/01/12	Institute of Microbiology of the AS CR
GCF_004421185.1	2019/03/27	2019/03/29	University of Bergen
GCF_009765685.1	2019/12/18	2020/01/12	Institute of Microbiology of the AS CR
GCF_009914725.1	2020/01/22	2020/03/08	National Institute of Advanced Industrial Science and Technology
GCF_002355995.1	2016/11/26	2020/12/30	Kyushu University
GCF_001708385.1	2016/08/23	2016/12/20	BioInformatics Center
GCF_000508305.1	2013/12/16	2016/12/30	Center for Genomic Sciences
GCF_000016605.1	2007/04/25	2021/04/14	US DOE Joint Genome Institute
GCF_000953475.1	2014/10/06	2020/12/16	LEIDEN UNIVERSITY MEDICAL CENTER
GCF_003722315.1	2018/11/13	2021/04/14	University of Exeter
GCF_001562115.1	2016/02/17	2021/04/14	University Miguel Hernandez
GCF_000027065.2	2011/02/17	2020/12/16	Technische Universitaet Muenchen
GCF_000831005.1	2014/02/05	2020/12/30	Swinburne University of Technology
GCF_000789255.1	2014/12/04	2021/04/05	Centre Bioengineering RAS
GCF_000967895.1	2013/05/31	2020/12/16	Max Planck Institute for Molecular Genetics
GCF_000237975.1	2011/12/14	2015/08/25	DOE Joint Genome Institute
GCF_000306725.1	2012/10/18	2019/10/03	Beijing Institute of Genomics
GCF_000247545.1	2012/02/10	2019/01/25	UCSC-Lowelab
GCF_000145985.1	2010/08/24	2015/08/25	US DOE Joint Genome Institute (JGI-PGF)
GCF_017298655.1	2021/03/08	2022/01/27	Institute of Microbiology
GCF_000953735.1	2014/09/30	2020/12/16	University of Tromso
GCF_005954745.1	2019/06/04	2021/04/14	Jiangsu University
GCA_001189275.1	2015/07/30	2015/07/30	Montana State University
GCF_900095815.1	2016/12/16	2020/09/30	CEBITEC
GCF_000200715.1	2006/11/20	2018/07/13	DOE Joint Genome Institute

GCF_000785785.1	2014/11/21	2022/12/22	CIAD, A.C.
GCF_018853935.1	2021/06/11	2021/09/02	Fundacion Ciencia y Vida
GCF_000482765.1	2013/10/25	2021/04/14	DOE Joint Genome Institute
GCF_010604095.1	2020/02/14	2022/05/10	Ares Genetics
GCF_015163485.1	2020/11/01	2021/04/05	The third Institute of Oceanography, Ministry of natural resources
GCF_000745905.1	2014/08/28	2022/05/10	DOE Joint Genome Institute
GCF_000336895.1	2013/02/04	2021/04/14	University of California, Davis
GCF_904846145.1	2020/11/21	2022/02/16	MAX PLANCK INSTITUTE FOR DEVELOPMENTAL BIOLOGY
GCA_904846265.1	2020/11/21	2020/11/23	MAX PLANCK INSTITUTE FOR DEVELOPMENTAL BIOLOGY
GCA_002506535.1	2017/10/10	2017/10/10	University of Queensland
GCA_001509375.1	2016/01/08	2016/01/08	Lawrence Berkeley National Lab
GCA_002502785.1	2017/10/10	2017/10/10	University of Queensland
GCA_002503885.1	2017/10/10	2017/10/10	University of Queensland
GCF_001411745.2	2015/10/23	2021/04/14	Universidad de Santiago de Chile
GCF_003353085.1	2018/08/01	2022/04/25	UFRJ
GCF_000690595.1	2016/05/26	2019/11/20	National Chung Hsing University, Taichung City, Taiwan R.O.C.
GCA_002279355.1	2017/08/16	2017/08/30	None
GCA_003023725.1	2018/03/27	2018/03/27	UC Berkeley
GCA_003023695.1	2018/03/27	2018/03/27	UC Berkeley
GCA_011049865.1	2020/03/02	2022/06/27	The University of Hong Kong
GCA_001516585.1	2016/01/15	2016/01/15	Montana State University
