# Supplementary material for: Environment and taxonomy shape the genomic signature of prokaryotic extremophiles
Source: Sci Rep. 2023 Sep 26;13:16105. doi: 10.1038/s41598-023-42518-y (PMC10522608; doi:10.1038/s41598-023-42518-y)
Supplement: Supplementary file 7 — Supplementary Information 7. [file 41598_2023_42518_MOESM7_ESM.pdf]

Supplementary Table S3A. **Accuracy summary for an alternative random sampling with pseudo-labels.** Additional computational experiments for supervised training with random labeling in the restriction-free scenario (*overlapping genera*), where the artificial pseudo-labels are sampled according to the distribution of the true environmental labels.

**Dataset**	**k -value**	**Classification Model Accuracy (%)**					
		**RBF SVM**	**Random Forrest**	**ANN**	**MLDSP 1**	**MLDSP 2**	**MLDSP 3**
Temperature	k = 1	22.26	29.42	31.77	22.58	32.27	29.60
	k = 2	23.25	28.10	27.09	33.61	31.61	33.61
	k = 3	21.57	30.93	32.29	33.44	28.93	33.78
	k = 4	23.08	24.73	27.60	28.43	28.93	26.09
	k = 5	23.42	28.59	28.93	24.08	28.60	26.59
	k = 6	21.23	30.10	31.77	26.09	27.59	29.10
pH	k = 1	51.20	46.61	50.53	47.85	44.09	46.77
	k = 2	51.14	52.72	51.67	50.00	41.94	50.54
	k = 3	44.71	50.09	52.25	56.99	52.15	56.99
	k = 4	44.68	51.99	50.61	51.61	51.61	47.85
	k = 5	42.95	51.02	53.74	48.39	51.61	56.45
	k = 6	50.14	54.72	51.63	47.85	53.23	55.91

Supplementary Table S3B. Additional computational experiments for supervised training with random labeling in the restricted scenario (non-*overlapping genera*), where the artificial pseudo-labels are sampled according to the distribution of the true environmental labels.

**Dataset**	**k -value**	**Classification Model Accuracy (%)**					
		**RBF SVM**	**Random Forrest**	**ANN**	**MLDSP 1**	**MLDSP 2**	**MLDSP 3**
Temperature	k = 1	23.91	32.93	30.15	26.30	32.40	28.10
	k = 2	22.59	27.56	32.80	34.60	33.30	34.40
	k = 3	26.90	29.09	32.60	30.80	30.60	30.60
	k = 4	26.24	28.23	29.94	28.40	29.40	25.40
	k = 5	21.42	28.30	28.31	27.30	29.40	24.90
	k = 6	25.07	29.23	27.58	26.60	30.60	27.90
pH	k = 1	41.78	55.89	48.60	47.80	46.20	46.80
	k = 2	51.52	54.14	45.79	49.50	44.60	48.90
	k = 3	45.33	50.11	47.06	57.00	54.80	55.90
	k = 4	52.29	50.66	50.25	53.80	54.80	48.40
	k = 5	47.35	52.33	54.80	44.60	54.80	49.50
	k = 6	54.08	47.78	46.11	44.10	56.50	51.10
