# Supplementary material for: Environment and taxonomy shape the genomic signature of prokaryotic extremophiles
Source: Sci Rep. 2023 Sep 26;13:16105. doi: 10.1038/s41598-023-42518-y (PMC10522608; doi:10.1038/s41598-023-42518-y)
Supplement: Supplementary file 8 — Supplementary Information 8. [file 41598_2023_42518_MOESM8_ESM.pdf]

Supplementary Table S4. Referenced articles supporting consistencies in *k*-mer associations for the temperature dataset. The following table describes the articles referenced in Table 5, which correlate to literature supporting the observed associated *k*-mers for each of the temperature-adapted extremophilic classes in the context of codon bias or amino acid compositional patterns.

Reference Link	Observation
https://doi.org/10.1186%2F1471-2164-15-1120	Increased valine, alanine, glycine, proline abundance in psychrophilic proteins
https://doi.org/10.1186/1471-2164-9-210	Decreased methionine, arginine, cysteine, histidine, increased asparagine in psychrophilic proteins
https://doi.org/10.1110%2Fps.072947007	Increase in alanine, cysteine, valine, glycine, and methionine in psychrophilic proteins, and a decrease in Asn, Lys, Tyr, Phe, and Glu
https://doi.org/10.1101/gr.1180903	Increase in glutamine and threonine, and decrease in leucine in psychrophilic proteins. Also, decreased Gln/Thr/His/Ser at higher OGT (thermophilic range) and increased Glu (glutamic acid)
https://doi.org/10.1074/jbc.m802158200	Increase glutamate for cold stability in psychrophilic proteins
https://doi.org/10.1093/femsec/fiy023	Increase in glycine/serine, decrease in aspartic/glutamic acids
https://doi.org/10.1016/S0969-2126(00)00133-7	Low serine, lower isoleucine abundance in mesophiles
https://doi.org/10.1038/s41598-020-58825-7	Gln and Met have increased abundance in mesophiles
https://doi.org/10.1093/protein/13.3.179	Increased proline abundance in mesophiles relative to thermophiles
https://doi.org/10.1371/journal.pcbi.0030005	Support for the IVYWREL adaptation set for increase OGT
https://doi.org/10.1002/prot.25866	Increased proline (codon=CCN) due to increased GC% in thermophiles
https://doi.org/10.1186/gb-2002-3-8-preprint0006	Gly, Lys, Tyr, and Ile are preferred in thermophilic organisms
https://doi.org/10.1002/prot.25866	Threonine and serine are rare in thermophiles and frequent in hyperthermophiles, while prolines are rare in hyperthermophiles
https://doi.org/10.1186/gb-2002-3-8-preprint0006	More charged (Glu, Arg, Lys) and fewer uncharged polar residues (Ser, Thr, Asn, Gln, His, Cys), increased residue hydrophobicity (Ile, Val), and increased residue volume (Tyr)
