# Supplementary material for: Environment and taxonomy shape the genomic signature of prokaryotic extremophiles
Source: Sci Rep. 2023 Sep 26;13:16105. doi: 10.1038/s41598-023-42518-y (PMC10522608; doi:10.1038/s41598-023-42518-y)
Supplement: Supplementary file 10 — Supplementary Information 10. [file 41598_2023_42518_MOESM10_ESM.pdf]

Supplementary Table S6. Characterizing phenotypic traits of candidate species and their isolating environment. The following table describes various phenotypic traits attributed to each of the candidate species, and the geochemical information associated with the environments the microbes were initially isolated from. Coordinates are described in the Longitude/Latitude format.

**Phenotypic Trait**	***Pyrococcus furiosus***	***Thermococcus litoralis***	***Pyrococcus chitonophagus***	***Thermocrinis ruber***
References	•Fiala, G. & Stetter, K. O. (1986) DOI: 10.1007/BF00413027 •Capaccioni, B., Tassi, F. & Vaselli, O. (2001) DOI: 10.1016/S0377-0273(00)00284-5	•Neuner, A., Jannasch, H. W., Belkin, S. & Stetter, K. O. (1990) DOI: 10.1007/BF00247822 •Orlando, V., Franco, T., Dario, T., Robert, P. J. & Antonio, C. (2011) DOI: 10.1016/j.proeps.2011. 11.007	•Huber, R. et al. (1995) DOI: 10.1007/BF02529959 •Bazylinski, D. A., Farrington, J. W. & Jannasch, H. W. (1988) DOI: 10.1016/0146-6380(88) 90146-5	•Clifton, C., Walters, C. & Simoneit, B. (1990) DOI: 10.1016/0883-2927(90)90047-9 •Huber, R. et al. (1998) DOI: 10.1128/AEM.64.10.3576-3583.1998
Domain	Archaea	Archaea	Archaea	Bacteria
DSM Strain ID	3638	5473	10152	12173
pH Range	pH 5.0 to 9.0 (OGpH=7.0)	pH 4.0 to 8.0 (OGpH=6)	pH 3.5 to 9 (OGpH=6.7)	pH 7 to 8.5
Temperature Range	70 to 103ºC (OGT=100ºC)	55 to 98ºC (OGT=85ºC)	60 to 93ªC (OGT=75ºC)	44 to 89ºC (OGT=80ºC)
Salinity	0.5 to 5% NaCl	1.8 to 6.5% NaCl (optimum 2.5%)	1.8 to 6.5% NaCl (optimum 2.5%)	0 to 0.4% NaCl
Cell Shape	Slightly irregular cocci	Round to irregular cocci	Round to irregular cocci	Rod-shaped cells
Gram Stain	Negative	Negative	Negative	Negative
Motility	Monopolar polytrichous flagella	Non-flagellated, non-motile	Monopolar polytrichous flagella	Monopolar polytrichous flagella
S-Layer Protein Composition	Hexagonal lattice	Hexagonal lattice	Hexagonal lattice	No evidence of a regularly arrayed SLP
Oxygen Tolerance	Anaerobic	Anaerobic	Anaerobic	Aerobic
Genome Size	1.89 Mb	1.82 Mb	1.95 Mb	1.52 Mb
Intergenic Sequence Composition	Pseudogenes and IS elements present	Pseudogenes and IS elements present	Pseudogenes and IS elements present	Pseudogenes present
Geographic Source	Submarine solfataric field in the bay of Porto di Levante, Vulcano Island, Italy.	Shallow submarine solfataras near the beach of Lucrino, Bay of Naples, Italy	Smoker site, Guaymas Basin, Gulf of California, Mexico	Octopus Spring, Yellowstone National Park, Wyoming, USA
Coordinates of Isolating Environment	38.41341, 14.9620271	40.822111, 14.090833	20.8333, -109.1	44.543652, -110.79792
Hydrocarbon Emissions	Evidence for gas discharges containing light hydrocarbons	Evidence for gas discharges containing light hydrocarbons	Evidence for gas discharges containing petroleum-like substances	Evidence for hydrothermal petroleum-like substances in other hot springs atop the caldera
